# New Highly Oxygenated Germacranolides from *Carpesium divaricatum* and their Cytotoxic Activity

**DOI:** 10.1038/srep27237

**Published:** 2016-06-06

**Authors:** Tao Zhang, Jin-Guang Si, Qiu-Bo Zhang, Gang Ding, Zhong-Mei Zou

**Affiliations:** 1 Institute of Medicinal Plant Development, Chinese Academy of Medical Sciences and Peking Union Medical College, Beijing 100193, P.R. China; 2 School of Pharmacy, Henan University of Traditional Chinese Medicine, Zhengzhou 450046, P.R. China

## Abstract

Eight highly oxygenated germacranolides (**1**–**8**) including four new ones (**2**–**5**) were isolated from the whole plant of *Carpesium divaricatum*. The planar structures and relative configurations of the new compounds were determined by NMR experiment and HRESIMS data. The absolute configuration of **1** was established by circular dichroism (CD) method and X-ray diffraction, and the stereochemistry of the new compounds **2**–**5** were determined by similar CD spectra with **1**. Compound **2** is the first hydroperoxyl germacrane from the genus *Carpesium*. The ^13^C NMR data of **1**, NMR data of **6**–**7**, and their absolute configurations were reported for the first time. Two new compounds (**2** and **4**) and two known compounds (**6** and **8**) exhibited potent cytotoxicity against human cervical cancer (HeLa) cells, superior to that of the positive control doxorubicin.

*Carpesium divaricatum* Sieb.et Zucc, belonging to the genus *Carpesium* (Asteraceae), is widely distributed in China, traditionally used for the treatment of fevers, colds, bruises, and inflammatory diseases^1^,^2^,^3^,^4^,^5^. The constituents of this plant have been previously investigated and shown to contain a number of sesquiterpenoids^6^,^7^,^8^,^9^. Previous investigations indicate that sesquiterpene lactones possessing an α-methylene-γ-lactone moiety in the structure have cytotoxic activity to human cancer cells^9^,^10^,^11^,^12^,^13^,^14^. Recently, *Carpesium* plants have attracted much attention due to eleven isolated sesquiterpene lactone dimers with novel skeletons displaying significantly cytotoxic activity^15^,^16^,^17^,^18^. The parent nucleus of the germacranes contains a special ten member ring with different post-modification to produce diverse structural features. A survey of the literature has shown that a large number of germacranolides are isolated from the genus *Carpesium*, but their absolute configurations have rarely been reported^6^,^7^,^8^,^12^,^13^,^19^,^20^,^21^,^22^,^23^.

In our ongoing search for new/novel and bioactive products from the medicinal plants in China, four new (**2**–**5**) and four known (**1** and **6**–**8**) germacranolides were isolated from the whole plant of *C. divaricatum*. In this paper, the structural elucidation including absolute configuration and bioactive evaluation of these compounds were present.

## Results and Discussion

**Figure 1:**
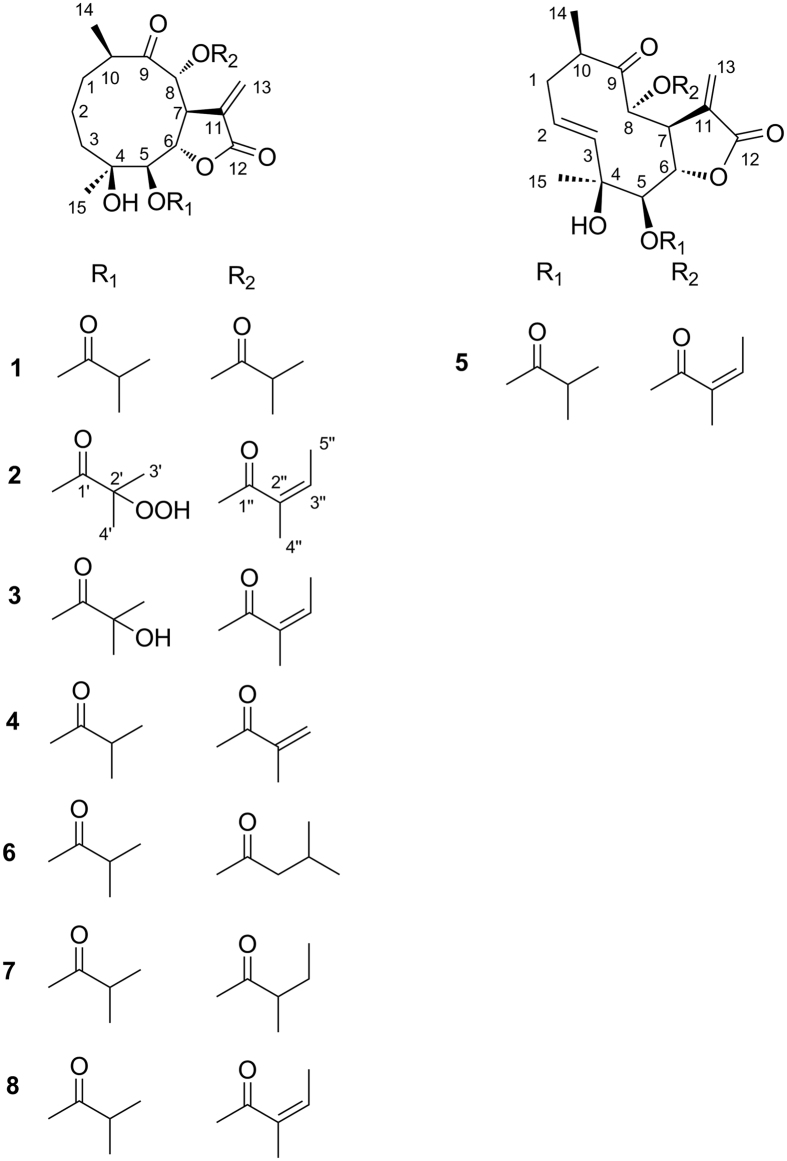
chemical structures of compounds **1–8**.

**Figure 2:**
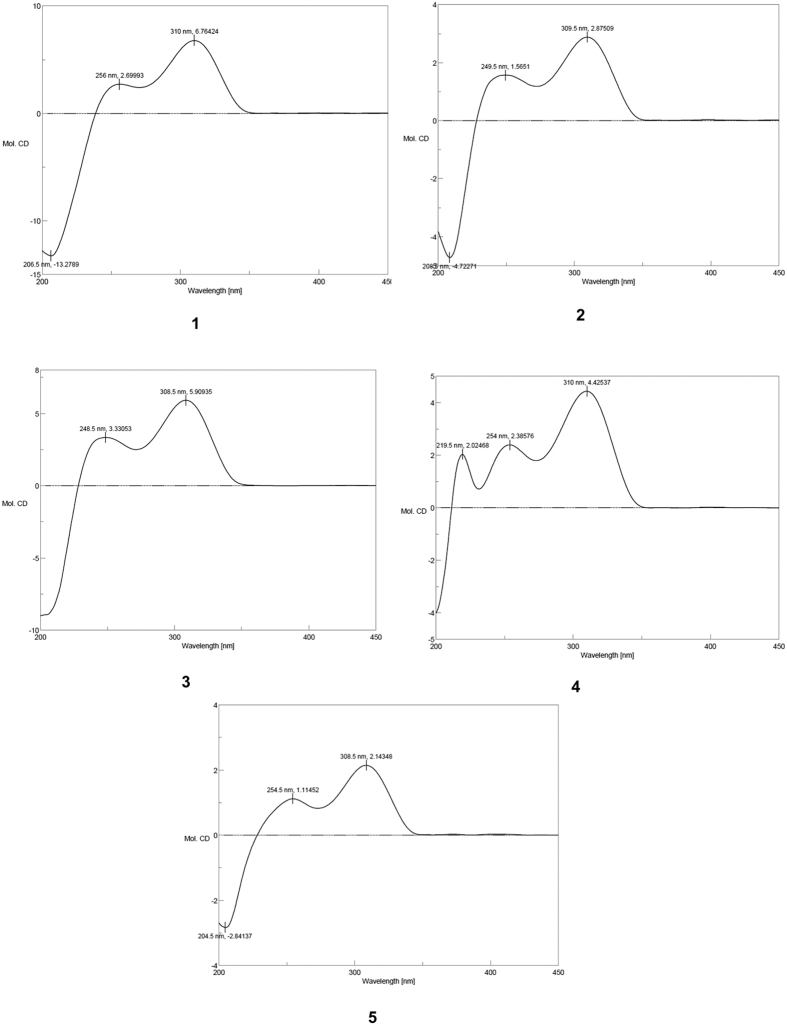
CD spectra of compounds **1**–**5**.

**Figure 3:**
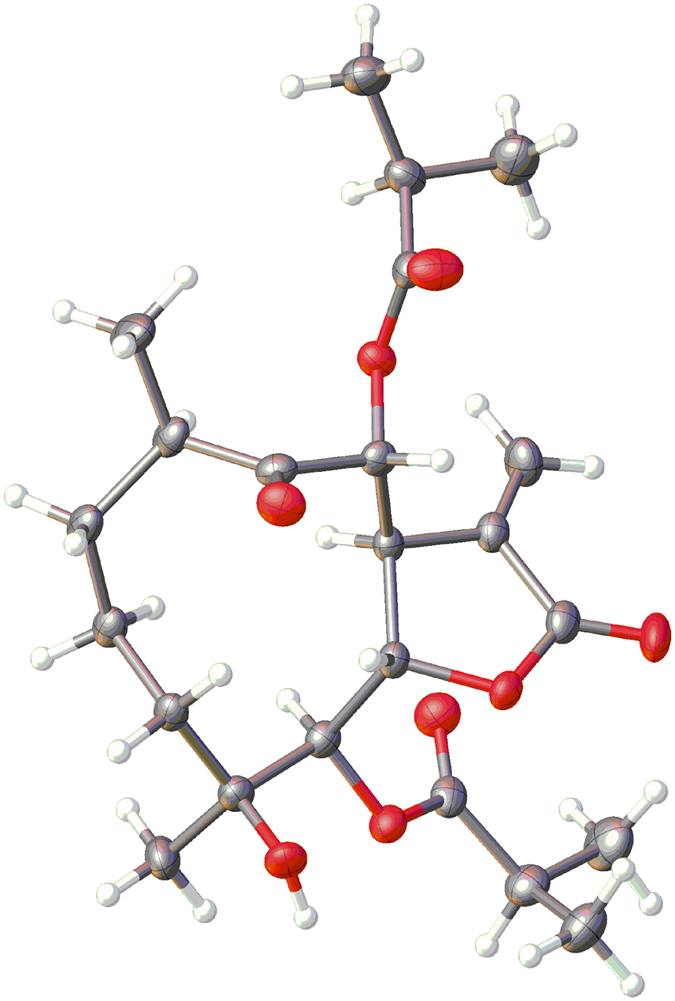
X-ray ORTEP drawing of 1.

Compound **1** (Fig. 1) was identified as incaspitolide A (**1**)^24^, by comparison of its MS, ^1^H NMR data, as well as optical rotation data with reported data. However, its ^13^C NMR data have not been reported and absolute configuration has not been determined. The^13^C NMR data was assigned by ^1^H-^1^H COSY and HSQC spectral data. The CD spectrum (Fig. 2) of **1** exhibited two positive Cotton effects at near 252 nm (α-methylene-γ-lactone region) and 294 nm (ketone n, π* region), supporting 6*S*, 7*S* configuration^12^. Fortunately, a suitable crystal was obtained for X-ray diffraction to confirm the absolute configuration. The X-ray crystallographic analysis [flack parameter: −0.02 (10)] established unambiguously the absolute configuration of **1** to be 4*S*, 5*R*, 6*S*, 7*S*, 8*R* and 10*R* (Fig. 3). Herein, the ^13^C NMR data and absolute configuration of **1** were reported for the first time.

**Figure 4:**
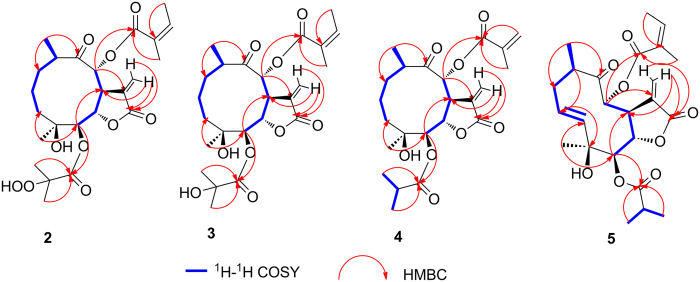
Key ^1^H-^1^H COSY and HMBC correlations of compounds **2**–**5**.

Compound **2** was obtained as white needles. The molecular formula was assigned as C_24_H_34_O_10_ on the basis of the positive-ion HRESIMS peak at *m/z* 505.2036 [M + Na]^+^, together with its ^1^H and ^13^C NMR data (Tables 1 and 2). Its IR spectrum showed hydroxyl (3400 cm^−1^) and carbonyl (1775 and 1729 cm^−1^) absorptions. The ^1^H and ^13^C NMR spectra of **2** showed an α-methylene-γ-lactone at *δ*_H_ 6.27 (1H, d, *J* = 2.0 Hz, Ha-13) and 5.96 (1H, d, *J* = 2.0 Hz, Hb-13), *δ*_C_ 134.7 (C-11), 127.4 (C-13) and 170.6 (C-12); three carbonyl carbons at *δ*_C_ 213.5 (C-9), 175.0 (C-1′) and 167.1 (C-1″); an oxygenated quaternary carbons at *δ*_C_ 73.9 (C-4); five methines including three oxygenated ones at *δ*_H_ 4.75 (1H, d, *J* = 6.5 Hz, H-5), 4.63 (1H, dd, *J* = 6.5, 2.0 Hz, H-6), 3.87 (1H, dd, *J* = 11.5, 2.0 Hz, H-7), 4.92 (1H, d, *J* = 11.0 Hz, H-8) and 3.27 (1H, m, H-10), *δ*_C_ 79.4 (C-5), 73.3 (C-6), 46.3 (C-7), 79.8 (C-8), and 42.7 (C-10); and two methyl groups at *δ*_H_ 1.01 (3H, d, *J* = 7.0 Hz, CH_3_-14), 1.19 (3H, s, CH_3_-15). These signals (^1^H and ^13^C NMR data) of **2** were similar to those of **8**^12^ (Table S8.2, Supplementary Information), except for the ester residue at C-5. The singlet signals of 3′-Me and 4′-Me together with chemical shift difference of C-2′ (*δ* 34.9 → 84.8) implied that C-2′ was an oxygenated quaternary carbon compared with **8**. Considering the chemical shift value of C-2′ (*δ* 84.8) and molecular formula of **2** confirmed that a hydroperoxyl moiety was attached at C-2′ 25,26,27, which was further confirmed by the HRESIMS with fragment peaks at *m/z* 345.1645 [M + 1-H_2_O-HOiBu-OOH]^+^ and 245.1102 [M + 1-H_2_O-HOiBu-OOH-HOAng]^+^. The HMBC correlations from both of H_3_-3′ (*δ*_H_ 1.46, 3H, s) and H_3_-4′ (*δ*_H_ 1.42, 3H, s) to C-1′ (*δ*c 175.0) and C-2′ (*δ*c 84.8) allowed a reasonable connection of the hydroperoxyl moiety to C-2′. The ^1^H-^1^H COSY spectrum (Fig. 4) showed two partial structure sequences for **2**: CH_2_(3)CH_2_(2)CH_2_(1)CH(10)CH_3_(14) and CH(5)CH(6)CH(7)CH(8). The C–C interconnectivity of all fragments was established from the HMBC spectrum (Fig. 4) as correlations of H-15 with C-3 and C-5, H-14 with C-1 and C-9, H-13 with C-7 and C-12, H-8 with C-1″ (ester carbonyl of angeloyloxy group), and H-5 with C-1′ (ester carbonyl of 2′-hydroperoxyl-isobutyryloxy group). On the basis of these data, the planar structure of **2** was established.

The relative configuration of **2** was determined by analysis of ROESY data. The key NOE correlations of H-8/H-6, H-7/H-10, H-7/H-5 and H-5/H_3_-15 indicated that **2** had the same relative configuration as **1**. The CD spectrum of **2** showed two positive Cotton effects at near 252 and 294 nm, which closely resembled those of **1**. Similar ROESY and CD data of **2** and **1** assigned the absolute configuration of **2** as 4*S*, 5*R*, 6*S*, 7*S*, 8*R* and 10*R*. Thus, the structure of compound **2** was defined as shown, named divarolide A.

Compounds **3**–**4** possessed molecular formulas of C_24_H_34_O_9_ and C_23_H_32_O_8_, from their HRESIMS at *m/z* 489.2108 [M + Na]^+^, and *m/z* 459.1971 [M + Na]^+^, respectively. The ^1^H and ^13^C NMR data of **3–4** were similar to those of incaspitolide A (**1**)^24^, except that the 2′-hydroxy-isobutyryloxy group at C-5 and the angeloyloxy group at C-8 in **3** were observed in place of two isobutyryloxy groups in **1**, and an isobutyryloxy group at C-8 in **1** was replaced by the 2-methylacryloyl group in **4**, respectively. These observations were confirmed by analyses of relevant ^1^H-^1^H COSY, HSQC and HMBC data (Fig. 4). The relative configurations of **3–4** were determined to be the same as that of **1** by comparison of ROESY data for relevant protons. Similar CD data of **3–4** and **1** revealed the same absolute configurations of **3–4** as that of **1**. Thus, the structures of compounds **3–4** were established as shown, named divarolide B and divarolide C, respectively.

The molecular formula of compound **5** was assigned as C_24_H_32_O_8_ by HRESIMS (471.1988 [M + Na]^+^). A comparison of the NMR data of **5** with those of **8** suggested that both of them had the same substituted groups at C-5 and C-8, but that the two mutually coupled methylene units (C-2–C-3) in **8** were oxidized to an olefin moiety in **5**. The C-2/C-3 double bond was assigned *E*-geometry on the basis of the large coupling constant observed for olefinic protons (17.0 Hz). The H-^1^H COSY, HSQC and HMBC spectra (Fig. 4) of **5** confirmed this observation, leading to the assignment of its planar structure. The relative and absolute configurations of **5** were deduced to be the same as those of **1**, on the basis of similar ROESY and CD data. Thus, the structure of compound **5** was elucidated as shown, named divarolide D.

Compounds **6**–**7** shared the same molecular formula C_24_H_36_O_8_, established from their HRESIMS at *m/z* 475.2317 [M + Na]^+^ and *m/z* 475.2305 [M + Na]^+^. The ^1^H and ^13^C NMR data of **6–7** showed a great similarity with those of **1**, except for the ester residues at C-8. The isobutyryloxy group at C-8 in **1** was placed by a 3-methylbutyry- loxy group in **6** and the 2-methylbutyryloxy group of **7**, respectively. Compounds **6**–**7** have been reported as a mixture from *Inula cuspidata*^24^. Actually, the exact linkage sites of the substituted groups have not been determined in the previous report and the authors speculate the mixture may contain two pairs of mixtures (incaspitolide B and C). Although the isolation of **6**–**7** is a huge challenge as they are highly oxygenated and similar, both of them were separated successfully in the present paper. Similarly, their relative and absolute configurations were determined as same as those of **1** by comparison of the ROESY and CD data. Thus, the structures of compounds **6–7** were established as shown, named incaspitolide B_1_ and incaspitolide B_2_, respectively.

Compound **8** was a known analogue of **1–7**, identified as (4*S*, 5*R*, 6*S*, 7*S*, 8*R*, 10*R*)-8-angeloyloxy-4-hydroxy-5-isobutyryloxy-9-oxo-germacran-7, 12-olide, by comparison of its MS, NMR and optical rotation data with reported data^12^.

Compounds **1**, **2**, **4** and **6**–**8** were obtained in sufficient amounts to be evaluated for their cytotoxic activity against human cervical cancer (HeLa), hepatocellular cancer (Hep G2), stomach cancer (MGC-803), and lung cancer (A549) cell lines. All evaluated compounds exhibited strong cytotoxicity against HeLa (IC_50_ values of 4.36, 0.83, 1.18, 0.57, 3.58 and 1.70 μM), Hep G2 (IC_50_ values of 6.41, 8.40, 14.20, 18.10, 9.55 and 8.28 μM), and MGC-803 (IC_50_ values of 4.63, 4.48, 2.93, 3.49, 4.63 and 2.70 μM) cell lines, but only compounds **2**, **4**, **6** and **8** had IC_50_ values of 0.83, 1.18, 0.57 and 1.70 μM against HeLa cell lines, superior to that of the positive control doxorubicin (IC_50_ value 2.21 μM). Besides, new compound **2** also displayed strong cytotoxicity against A549 with IC_50_ value of 8.93 μM (the positive control doxorubicin showed IC_50_ value of 4.18 μM).

In conclusion, eight highly oxygenated germacranolides including four new ones (**2**–**5**) were isolated from the whole plant of *C. divaricatum*. To the best of our knowledge, this is the first report of hydroperoxyl germacrane from the genus *Carpesium*. New compounds **2** and **4**, as well as known compounds **6** and **8**, exhibited potent cytotoxicity against HeLa cell lines, superior to that of the positive control doxorubicin. These findings are an important addition to the present knowledge on the structurally diverse and biologically important germacranolide family.

## Methods

### General Experimental Procedures

Optical rotations were measured on a Perkin-Elmer 241 polarimeter (Perkin-Elmer, Waltham, MA, USA) and UV spectra were recorded on Shimadzu UV-2501 PC (Shimadzu, Kyoto, Japan). IR data were recorded using a Shimadzu FTIR-8400S spectrophotometer (Shimadzu, Kyoto, Japan). ^1^H and ^13^C-NMR data were acquired with Bruker 500 instruments (Bruker, Rheinstetten, Germany) using the solvent signals (CD_3_OD: *δ*_H_ 4.87/*δ*_C_ 49.0 ppm;) as references. HRESIMS data were acquired using Q-TOF analyzer in SYNAPT HDMS system (Waters, Milford, MA, USA). CD spectra were recorded on a JASCO J-815 Spectropolarimeter (Jasco, Tokyo, Japan).

X-ray diffraction data were collected on the Agilent GEMINI^TM^E instrument (CrysAlisPro software, Version 1.171.35.11; Agilent, Santa Clara, CA, USA). HPLC was performed using Waters 2535 system (Waters, Milford, MA, USA) with the following components: preparative column, a Daisogel-C_18_-100A (10 μm, 30 × 250 mm, ChuangXinTongHeng Sci.&Tech., Beijing, China) and a YMC-Pack ODS-A column (5 μm, 10 × 250 mm, YMC, Kyoto, Japan); and detector, Waters 2489 UV. Sephadex LH-20 (40–70 μm, Pharmacia Biotech AB, Uppsala, Sweden), silica gel (60–100, 100–200, and 200–300 mesh) and silica gel GF254 sheets (0.20–0.25 mm) (Qingdao Marine Chemical Plant, Qingdao, China) were used for column chromatography and TLC, respectively. TLC spots were visualized under UV light and by dipping into 5% H_2_SO_4_ in EtOH followed by heating.

### Plant Material

The whole plant of *C. divaricatum* were collected from EnShi, Sichuan province of China, in August, 2013. They were identified by Prof. Ben-Gang Zhang of Institute of Medicinal Plant Development. A voucher specimen (No. 20130828) was deposited in the Institute of Medicinal Plant Development, Chinese Academy of Medical Sciences and Peking Union Medical College (CAMS & PUMC), China.

### Extraction and Isolation

The air-dried plants (9 kg) were extracted three times (7 days each time) with EtOH–H_2_O (95:5) at room temperature. The combined extract was concentrated under reduced pressure to furnish a dark brown residue (570 g), which was suspended in H_2_O and partitioned in turn with petroleum ether (bp 60–90 °C), EtOAc, and *n*-BuOH. The EtOAc extract (207 g) was separated chromatographically on silica gel column (60–100 mesh, 16 × 20 cm) with a gradient mixture of CH_2_Cl_2_–MeOH (100:1, 60:1, 30:1, 15:1, and 6:1) as eluent. Five fractions (fraction A–E) were collected according to thin layer chromatography (TLC) analysis. Fraction A (CH_2_Cl_2_–MeOH, 100:1, 140 g) was separated by silica gel column chromatography (CC) (100–200 mesh, 16 × 20 cm) with petroleum ether–aceton (50:1, 25:1, 20:1, 15:1, 12:1, 10:1, 7:1, 5:1, 3:1 and 1:1) as eluent to give fraction A_1_–A_11_. Fraction A_7_ (petroleum ether–aceton, 7:1, 8 g) was separated by Sephadex LH-20 CC (5 × 200 cm, MeOH) to give Fr.A_7_S_1_–Fr.A_7_S_3._ Fraction A_7_S_2_ (MeOH–H_2_O, 5 g) was purified using preparative HPLC (Daisogel–C_18_–100A, 10 μm; 250 × 30 mm; 20 mL/min, 70% MeOH in H_2_O) to yield **8** (3.9 g) and a mixture of **1**–**7** (800 mg). The mixture of **1**–**7** (800 mg) was further purified using semipreparative HPLC (60–90% MeOH in H_2_O for 40 min; 40–80% MeCN in H_2_O for 40 min) to yield **1** (100 mg), **2** (5 mg), **3** (3.5 mg), **4** (8 mg), **5** (2.6 mg), **6** (30 mg) and **7** (25 mg).

Incaspitolide A (**1**): white needles (CH_3_OH), 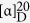 +57.7 (*c* 0.25, CHCl_3_); UV (MeOH) λ_max_(logε): 207 (4.35) nm, IR (neat) ν_max_: 3544, 1776, 1746, 1720, 1666 cm^−1^; CD (MeOH) 256 (Δε +0.09), 310 (Δε +0.22), 207 (Δε −0.44) nm; HRESIMS (pos.): *m/z* 461.2154 [M + Na]^+^ (calcd for C_23_H_34_O_8_Na, 461.2151); ^1^H NMR data see Table 1, ^13^C NMR data see Table 2.

Divarolide A (**2**): white needles (CH_3_OH), 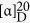 +35.7 (*c* 0.2, CHCl_3_); UV (MeOH) λ_max_(logε): 214 (3.99) nm, IR (neat) ν_max_: 3400, 1775, 1729, 1645 cm^−1^; CD (MeOH) 250 (Δε +0.06), 310 (Δε +0.11), 209 (Δε −0.17) nm; HRESIMS (pos.): *m/z* 505.2036 [M + Na]^+^ (calcd for C_24_H_34_O_10_Na, 505.2050); ^1^H NMR data see Table 1, ^13^C NMR data see Table 2.

Divarolide B (**3**): white needles (CH_3_OH), 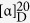 +121.0 (*c* 0.11, CHCl_3_); UV (MeOH) λ_max_(logε): 213 (4.22) nm, IR (neat) ν_max_: 3500, 1768, 1726, 1643 cm^−1^; CD (MeOH) 249 (Δε +0.12), 309 (Δε +0.21); HRESIMS (pos.): *m/z* 489.2108 [M + Na]^+^ (calcd for C_24_H_34_O_9_Na , 489.2101); ^1^H NMR data see Table 1, ^13^C NMR data see Table 2.

Divarolide C (**4**): white needles (CH_3_OH), 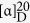 +48.7 (*c* 0.10, CHCl_3_); UV (MeOH) λ_max_(logε): 208 (3.93) nm, IR (neat) ν_max_: 3508, 1776, 1728, 1665, 1636 cm^−1^; CD (MeOH) 220 (Δε +0.07), 254 (Δε +0.08), 310 (Δε +0.15) nm; HRESIMS (pos.): *m/z* 459.1971 [M + Na]^+^ (calcd for C_23_H_32_O_8_Na, 459.1995); ^1^H NMR data see Table 1, ^13^C NMR data see Table 2.

Divarolide D (**5**): white needles (CH_3_OH), 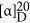 +18.9 (*c* 0.25, CHCl_3_); UV (MeOH) λ_max_(logε): 210 (4.03) nm, IR (neat) ν_max_: 3514, 1771, 1728, 1665, 1646 cm^−1^; CD (MeOH) 255 (Δε +0.04), 309 (Δε +0.07), 205 (Δε −0.10) nm; HRESIMS (pos.): *m/z* 471.1988 [M + Na]^+^ (calcd for C_24_H_32_O_8_Na, 471.1995); ^1^H NMR data see Table 1, ^13^C NMR data see Table 2.

Incaspitolide B_1_ (**6**): white needles (CH_3_OH), 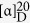 +123.1 (*c* 0.15, CHCl_3_); UV (MeOH) λ_max_(logε): 210 (3.83) nm, IR (neat) ν_max_: 3529, 1777, 1746, 1721, 1665 cm^−1^; CD (MeOH) 256 (Δε +0.08), 310 (Δε +0.19), 207 (Δε −0.39) nm; HRESIMS (pos.): *m/z* 475.2317 [M + Na]^+^ (calcd for C_24_H_36_O_8_Na, 475.2308); ^1^H NMR data see Table 1, ^13^C NMR data see Table 2.

Incaspitolide B_2_ (**7**): white needles (CH_3_OH), 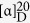 +31.2 (*c* 0.18, CHCl_3_); UV (MeOH) λ_max_(logε): 202 (3.77) nm, IR (neat) ν_max_: 3532, 1781, 1746, 1719, 1667 cm^−1^; CD (MeOH) 257 (Δε +0.06), 310 (Δε +0.17), 206 (Δε −0.34) nm; HRESIMS (pos.): *m/z* 475.2305 [M + Na]^+^ (calcd for C_24_H_36_O_8_Na, 475.2308); ^1^H NMR data see Table 1, ^13^C NMR data see Table 2.

### X-ray crystal structure analysis

X-ray diffraction data were collected on the Agilent GEMINI^TM^E instrument (CrysAlisPro software, Version 1.171.35.11), with enhanced Cu Kα radiation (λ = 1.54184 Å). The structure was solved by direct methods and refined by full-matrix least-squares techniques (SHELXL-97). All non-hydrogen atoms were refined with anisotropic thermal parameters. Hydrogen atoms were located by geometrical calculations and from positions in the electron density maps. Crystallographic data (excluding structure factors) for **1** in this paper has been deposited with the Cambridge Crystallographic Data Centre (deposition number CCDC 1441395). Copies of the data can be obtained, free of charge, on application to CCDC, 12 Union Road, Cambridge CB2 1EZ, UK (fax: +44 12 23336033 or e-mail: deposit@ccdc.cam.ac.uk).

A colorless triclinic crystal (0.50 × 0.50 × 0.40 mm) of **1** was obtained from CH_2_Cl_2_ –MeOH (3:1). Crystal data: 3C_23_H_34_O_8_·2H_2_O (*M* = 459.86), *T* = 105.5 K, triclinic, space group *P*_1_, a = 9.7574(3) Å, b = 10.9450(5) Å, c = 18.3652(8) Å, *α* = 102.359 (4)°, *β* = 99.183(3)°, *γ* = 101.095(3)°, *V* = 1838.35(13) Å^3^, *Z* = 3, *ρ* = 1.246 mg/mm^3^, *μ*(Cu Kα) = 0.781 mm^−1^, measured reflections = 24294, unique reflections = 12313 (R_int_ = 0.0202), largest difference peak/hole = 1.010/−0.331 e Å^−3^, and flack parameter = −0.02(10). The final R indexes [*I* > 2*σ (I*)] were R_1_ = 0.0435, and wR_2_ = 0.1176. The final R indexes (all data) were R_1_ = 0.0448, and wR_2_ = 0.1191. The goodness of fit on F^2^ was 1.046.

### Cytotoxicity assays

The assay was run in triplicate. In a 96-well plate, each well was plated with 2 × 10^4^ cells. After cell attachment overnight, the medium was removed, and each well was treated with 100 μL of medium containing 0.1% DMSO or different concentrations of the test compounds and the positive control doxorubicin. The plate was incubated for 4 days at 37 °C in a humidified, 5% CO_2_ atmosphere. Cytotoxicity was determined using a modified 3-(4,5-dimethylthiazol-2-yl)-2,5-diphenyltetrazolium bromide (MTT) colorimetric assay^28^. After addition of 10 μL MTT solution (5 mg/mL), cells were incubated at 37 °C for 4 h. After adding 150 μL DMSO, cells were shaken to mix thoroughly. The absorbance of each well was measured at 490 nm in a Multiscan photometer. The IC_50_ values were calculated by SPSS software and listed in Table 3.

## Additional Information

**How to cite this article**: Zhang, T. *et al*. New Highly Oxygenated Germacranolides from *Carpesium divaricatum* and their Cytotoxic Activity. *Sci. Rep.* **6**, 27237; doi: 10.1038/srep27237 (2016).

## Supplementary Material

Supplementary Information

## Figures and Tables

**Table 1:** ^1^H NMR spectral data for compounds **1**–**7** (*J* in Hz within parentheses).

No.	1^a^	2^a^	3^a^	4^a^	5^a^	6^a^	7^a^
1	1.68 o^b^, 1.24 m	1.69 o, 1.24 m	1.69 o, 1.24 m	1.72 m, 1.24 m	2.37 m, 2.18 m	1.69 o, 1.24 m	1.71 o, 1.23 m
2	1.54 m, 1.44 m	1.57 m, 1.46 m	1.56 m, 1.45 m	1.58 m, 1.45 m	5.98 m	1.55 m, 1.45 m	1.54 m, 1.45 m
3	1.68 o, 1.68 o	1.69 o, 1.69 o	1.69 o, 1.69 o	1.69 o, 1.69 o	5.63 br d (17.0)	1.69 o, 1.69 o	1.71 o, 1.71 o
5	4.67 d (6.0)	4.75 d (6.5)	4.70 d (6.5)	4.69 d (6.0)	4.68 d (8.5)	4.68 d (6.0)	4.69 d (6.0)
6	4.58 dd (6.5, 2.0)	4.63 dd (6.5, 2.0)	4.62 dd (6.5, 1.5)	4.62 dd (6.0, 2.0)	4.40 br d (8.5)	4.59 dd (6.5, 2.0)	4.60 dd (6.0, 1.5)
7	3.79 dd (11.5, 2.0)	3.87 dd (11.5, 2.0)	3.87 dd (11.5, 1.5)	3.88 dd (11.5, 2.0)	3.64 dd (10.0, 1.5)	3.78 dd (11.5, 2.0)	3.81 dd (11.5, 1.5)
8	4.76 d (11.5)	4.92 d (11.0)	4.91 d (11.5)	4.92 d (11.0)	4.74 d (10.5)	4.84 d (11.5)	4.80 d (11.5)
10	3.24 m	3.27 m	3.27 m	3.29 m	3.43 m	3.24 m	3.25 m
13a	6.30 d (1.5)	6.27 d (2.0)	6.27 d (2.0)	6.27 d (1.5)	6.27 d (1.5)	6.29 d (2.0)	6.32 d (2.0)
13b	6.03 d (1.5)	5.96 d (2.0)	5.97 d (2.0)	5.98 d (1.5)	5.88 d (1.5)	6.03 d (2.0)	6.05 d (2.0)
14	1.00 d (6.5)	1.01 d (7.0)	1.01 d (7.0)	1.00 d (6.5)	1.07 d (6.5)	1.03 d (6.5)	1.04 d (7.0)
15	1.13 s	1.19 s	1.15 s	1.15 s	1.24 s	1.14 s	1.15 s
2′	2.69 o			2.68 m	2.67 m	2.67 m	2.68 m
3′	1.18 d (7.0)	1.46 s	1.45 s	1.19 d (7.0)	1.20 d (7.0)	1.19 d (7.0)	1.19 d (7.0)
4′	1.17 d (7.0)	1.42 s	1.41 s	1.17 d (7.0)	1.17 d (7.0)	1.17 d (7.0)	1.17 d (7.0)
2″	2.69 o					2.36 o, 2.36 o	2.53 m
3″	1.22 d (7.0)	6.30 q (6.5)	6.31 q (6.5)	6.31 dq (3.0, 1.0), 5.83 dq (3.0,1.0)	6.28 q (6.5)	2.09 m	1.71 m, 1.54 o
4″	1.15 d (7.0)	2.00 br s	2.00 br s	1.99 br s	1.95 br s	0.98 d (6.5)	1.19 d (7.0)
5″		1.98 dq (6.5, 1.5)	1.99 dq (6.5, 1.5)		1.97 dq (6.5, 1.5)	0.97 d (7.0)	0.94 t (9.0)

^a^Measured at 500 MHz in CD_3_OD.

^b^Overlapped with other signals.

**Table 2:** ^13^C NMR spectral data for compounds **1**–**7.**

No.	1^a^	2^a^	3^a^	4^a^	5^a^	6^a^	7^a^
1	22.8	22.8	22.8	22.9	36.5	22.9	22.7
2	37.8	37.6	37.5	37.7	131.0	37.7	37.8
3	34.5	34.5	34.5	34.4	130.9	34.5	34.7
4	73.8	73.9	74.0	73.8	75.5	73.8	73.8
5	78.6	79.4	79.3	78.7	78.5	78.6	78.4
6	73.2	73.3	73.7	73.1	73.4	73.2	73.3
7	46.2	46.3	46.2	46.3	46.0	46.2	46.2
8	80.0	79.8	79.8	80.3	79.9	80.0	79.8
9	213.5	213.5	213.5	213.3	210.7	213.5	213.5
10	42.4	42.7	42.7	42.7	44.4	42.5	42.3
11	134.7	134.7	134.7	134.9	134.9	134.8	134.6
12	170.7	170.6	170.6	170.7	170.8	170.7	170.7
13	127.6	127.4	127.4	127.4	127.4	127.6	127.7
14	20.9	20.9	20.9	20.8	18.6	21.0	21.0
15	24.8	24.8	24.8	24.8	25.9	24.8	24.9
1′	178.7	175.0	177.2	178.7	178.6	178.7	178.7
2′	34.9	84.8	73.2	34.9	35.1	34.9	34.9
3′	19.4	23.1	27.2	19.2	19.3	19.2	19.2
4′	19.2	22.7	27.7	19.2	19.4	19.2	19.2
1″	177.0	167.1	167.1	166.9	167.4	173.0	176.6
2″	35.1	127.6	127.6	136.8	127.6	43.6	42.2
3″	19.2	143.1	143.1	128.2	142.8	26.5	27.7
4″	19.1	20.7	20.7	18.4	20.6	22.7	16.8
5″		16.1	16.1		16.1	22.7	12.0

^a^Measured at 125MHz in CD_3_OD.

**Table 3:** Cytotoxicity of Compounds **1**, **2**, **4** and **6**–**8**.

compounds	IC_50_(μM)			
	HeLa	Hep G2	MGC-803	A549
**1**	4.36 ± 0.12	6.41 ± 0.23	4.63 ± 0.25	21.7 ± 1.41
**2**	0.83 ± 0.09	8.40 ± 0.84	4.48 ± 1.01	8.93 ± 1.73
**4**	1.18 ± 0.14	14.20 ± 0.21	2.93 ± 0.57	27.8 ± 2.34
**6**	0.57 ± 0.06	18.10 ± 0.72	3.49 ± 0.40	20.7 ± 0.84
**7**	3.58 ± 0.21	9.55 ± 0.27	4.63 ± 0.59	22.7 ± 0.94
**8**	1.70 ± 0.24	8.28 ± 0.64	2.70 ± 0.65	16.3 ± 1.41
doxorubicin	2.21 ± 0.18	5.40 ± 0.80	0.74 ± 0.05	4.18 ± 0.52

Values were mean ± SD. Doxorubicin, positive control. Cell lines: HeLa: cervical cancer, Hep G2: hepatocellular cancer, MGC-803: stomach cancer, and A549: lung cancer.
