# Supplementary material for: New Highly Oxygenated Germacranolides from Carpesium divaricatum and their Cytotoxic Activity
Source: Sci Rep. 2016 Jun 6;6:27237. doi: 10.1038/srep27237 (PMC4893730; doi:10.1038/srep27237)
Supplement: Supplementary Information [file srep27237-s1.pdf]

Supplementary Information

New Highly Oxygenated Germacranolides from *Carpesium divaricatum* and their Cytotoxic Activity

Tao Zhang,[1] Jin-Guang Si,[1,2] Qiu-Bo Zhang ,[1] Gang Ding,[1] and Zhong-Mei Zou[1*]

[1] Institute of Medicinal Plant Development, Chinese Academy of Medical Sciences and Peking Union Medical College, Beijing 100193, P. R. China.

[2] School of Pharmacy, Henan University of Traditional Chinese Medicine, Zhengzhou 450046, P.R. China.

*Correspondence and requests for materials should be addressed to Zhong-Mei Zou (Email: zmzou@implad.ac.cn; Tel: +86-010-57833290; Fax: +86-010-57833290)

Contents of Supplementary Information

	contents
Fig. S1.1	^{1}H NMR spectrum (500 MHz) of incaspitolide A (**1**) in CD_3OD
Fig. S1.2	^{13}C NMR spectrum (125 MHz) of incaspitolide A (**1**) in CD_3OD
Fig. S1.3	^{1}H-^{1}H COSY spectrum (500 MHz) of incaspitolide A (**1**) in CD_3OD
Fig. S1.4	HSQC spectrum (500 MHz) of incaspitolide A (**1**) in CD_3OD
Fig. S1.5	HMBC spectrum (500 MHz) of incaspitolide A (**1**) in CD_3OD
Fig. S1.6	ROESY spectrum (500 MHz) of incaspitolide A (**1**) in CD_3OD
Fig. S1.7	UV spectrum of incaspitolide A (**1**)
Fig. S1.8	IR spectrum of incaspitolide A (**1**)
Fig. S1.9	HRESIMS spectrum of incaspitolide A (**1**)
Fig. S1.10	CD spectrum of incaspitolide A (**1**)
Fig. S1.11	X-ray ORTEP drawing of incaspitolide A (**1**)
Fig. S2.1	^{1}H NMR spectrum (500 MHz) of divarolide A (**2**) in CD_3OD
Fig. S2.2	^{13}C NMR spectrum (500 MHz) of divarolide A (**2**) in CD_3OD
Fig. S2.3	^{1}H-^{1}H COSY spectrum (500 MHz) of divarolide A (**2**) in CD_3OD
Fig. S2.4	HSQC spectrum (500 MHz) of divarolide A (**2**) in CD_3OD
Fig. S2.5	HMBC spectrum (500 MHz) of divarolide A (**2**) in CD_3OD
Fig. S2.6	ROESY spectrum (500 MHz) of divarolide A (**2**) in CD_3OD
Fig. S2.7	UV spectrum of divarolide A (**2**)
Fig. S2.8	IR spectrum of divarolide A (**2**)
Fig. S2.9	HRESIMS spectrum of divarolide A (**2**)
Fig. S2.10	CD spectrum of divarolide A (**2**)
Fig. S3.1	^{1}H NMR spectrum (500 MHz) of divarolide B (**3**) in CD_3OD
Fig. S3.2	^{13}C NMR spectrum (500 MHz) of divarolide B (**3**) in CD_3OD
Fig. S3.3	^{1}H-^{1}H COSY spectrum (500 MHz) of divarolide B (**3**) in CD_3OD
Fig. S3.4	HSQC spectrum (500 MHz) of divarolide B (**3**) in CD_3OD
Fig. S3.5	HMBC spectrum (500 MHz) of divarolide B (**3**) in CD_3OD
Fig. S3.6	ROESY spectrum (500 MHz) of divarolide B (**3**) in CD_3OD
Fig. S3.7	UV spectrum of divarolide B (**3**)
Fig. S3.8	IR spectrum of divarolide B (**3**)
Fig. S3.9	HRESIMS spectrum of divarolide B (**3**)
Fig. S3.10	CD spectrum of divarolide B (**3**)
Fig. S4.1	^{1}H NMR spectrum (500 MHz) of divarolide C (**4**) in CD_3OD
Fig. S4.2	^{13}C NMR spectrum (500 MHz) of divarolide C (**4**) in CD_3OD
Fig. S4.3	^{1}H-^{1}H COSY spectrum (500 MHz) of divarolide C (**4**) in CD_3OD
Fig. S4.4	HSQC spectrum (500 MHz) of divarolide C (**4**) in CD_3OD
Fig. S4.5	HMBC spectrum (500 MHz) of divarolide C (**4**) in CD_3OD
Fig. S4.6	ROESY spectrum (500 MHz) of divarolide C (**4**) in CD_3OD
Fig. S4.7	UV spectrum of divarolide C (**4**)
Fig. S4.8	IR spectrum of divarolide C (**4**)
Fig. S4.9	HRESIMS spectrum of divarolide C (**4**)

Fig. S4.10	CD spectrum of divarolide C (**4**)
Fig. S5.1	^{1}H NMR spectrum (500 MHz) of divarolide D (**5**) in CD_3OD
Fig. S5.2	^{13}C NMR spectrum (500 MHz) of divarolide D (**5**) in CD_3OD
Fig. S5.3	^{1}H-^{1}H COSY spectrum (500 MHz) of divarolide D (**5**) in CD_3OD
Fig. S5.4	HSQC spectrum (500 MHz) of divarolide D (**5**) in CD_3OD
Fig. S5.5	HMBC spectrum (500 MHz) of divarolide D (**5**) in CD_3OD
Fig. S5.6	ROESY spectrum (500 MHz) of divarolide D (**5**) in CD_3OD
Fig. S5.7	UV spectrum of divarolide D (**5**)
Fig. S5.8	IR spectrum of divarolide D (**5**)
Fig. S5.9	HRESIMS spectrum of divarolide D (**5**)
Fig. S5.10	CD spectrum of divarolide D (**5**)
Fig. S6.1	^{1}H NMR spectrum (500 MHz) of incaspitolide B_1 (**6**) in CD_3OD
Fig. S6.2	^{13}C NMR spectrum (500 MHz) of incaspitolide B_1 (**6**) in CD_3OD
Fig. S6.3	^{1}H-^{1}H COSY spectrum (500 MHz) of incaspitolide B_1 (**6**) in CD_3OD
Fig. S6.4	HSQC spectrum (500 MHz) of incaspitolide B_1 (**6**) in CD_3OD
Fig. S6.5	HMBC spectrum (500 MHz) of incaspitolide B_1 (**6**) in CD_3OD
Fig. S6.6	ROESY spectrum (500 MHz) of incaspitolide B_1 (**6**) in CD_3OD
Fig. S6.7	UV spectrum of incaspitolide B_1 (**6**)
Fig. S6.8	IR spectrum of incaspitolide B_1 (**6**)
Fig. S6.9	HRESIMS spectrum of incaspitolide B_1 (**6**)
Fig. S6.10	CD spectrum of incaspitolide B_1 (**6**)
Fig. S7.1	^{1}H NMR spectrum (500 MHz) of incaspitolide B_2 (**7**) in CD_3OD
Fig. S7.2	^{13}C NMR spectrum (500 MHz) of incaspitolide B_2 (**7**) in CD_3OD
Fig. S7.3	^{1}H-^{1}H COSY spectrum (500 MHz) of incaspitolide B_2 (**7**) in CD_3OD
Fig. S7.4	HSQC spectrum (500 MHz) of incaspitolide B_2 (**7**) in CD_3OD
Fig. S7.5	HMBC spectrum (500 MHz) of incaspitolide B_2 (**7**) in CD_3OD
Fig. S7.6	ROESY spectrum (500 MHz) of incaspitolide B_2 (**7**) in CD_3OD
Fig. S7.7	UV spectrum of incaspitolide B_2 (**7**)
Fig. S7.8	IR spectrum of incaspitolide B_2 (**7**)
Fig. S7.9	HRESIMS spectrum of incaspitolide B_2 (**7**)
Fig. S7.10	CD spectrum of incaspitolide B_2 (**7**)
Fig. S8.1	CD spectrum of **8**
Table S8.2	^{1}H and ^{13}C NMR spectroscopic data of **8** in CD_3OD
Fig. S9	The concentration-response curves for cytotoxicity data

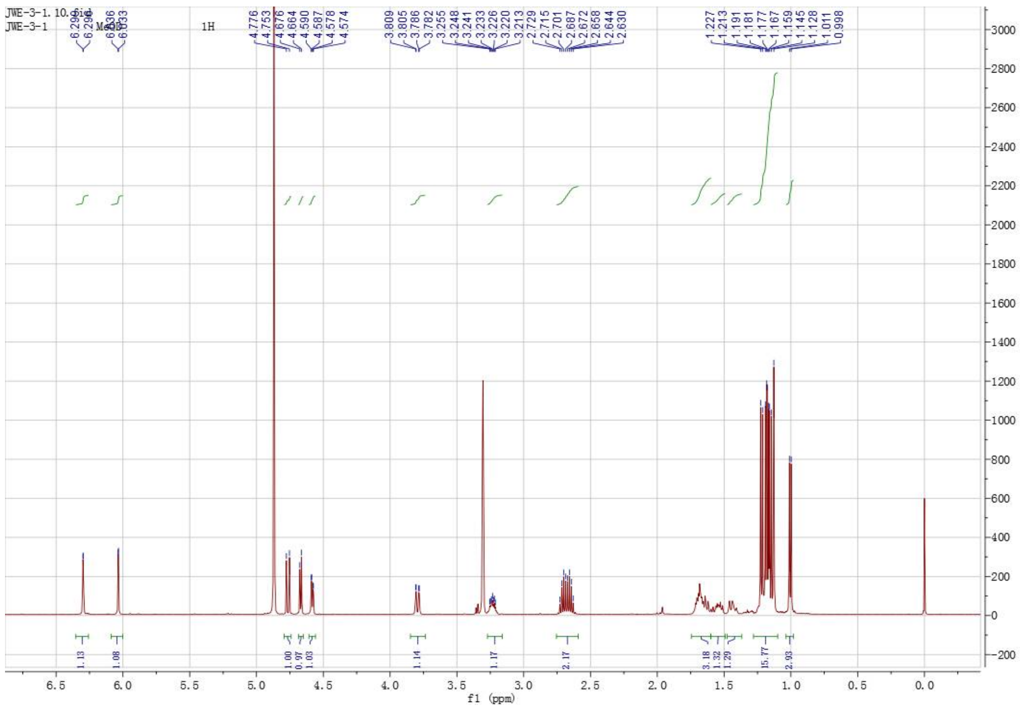

Fig. S1.1 ^{1}H NMR spectrum (500 MHz) of incaspitolide A (**1**) in CD_3OD

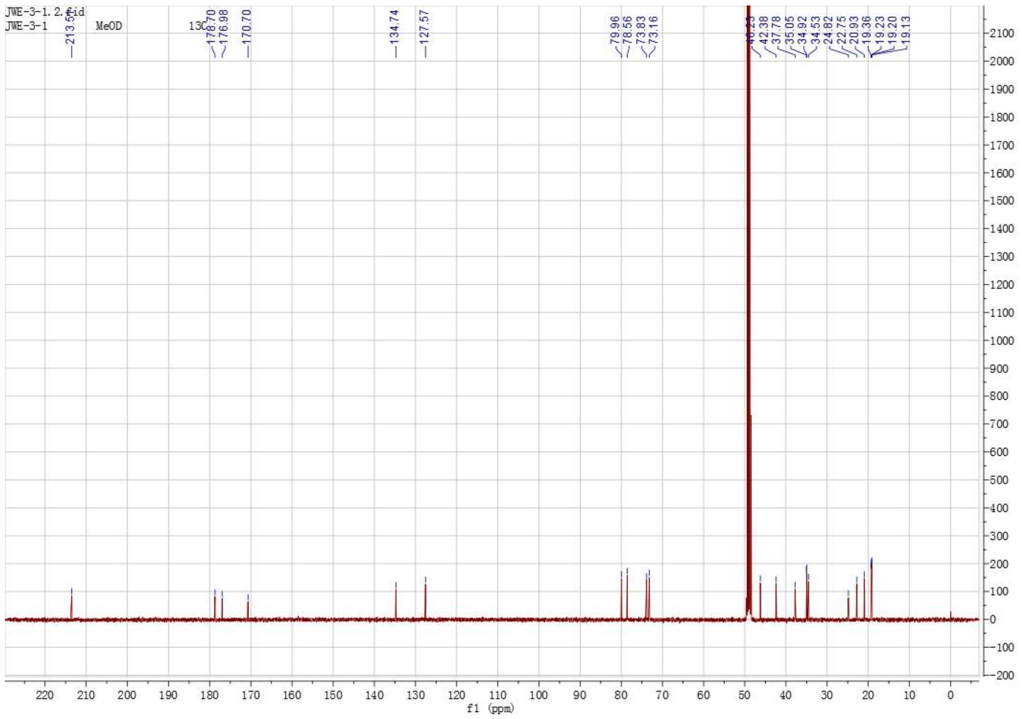

Fig. S1.2 ^{13}C NMR spectrum (500 MHz) of incaspitolide A (**1**) in CD_3OD

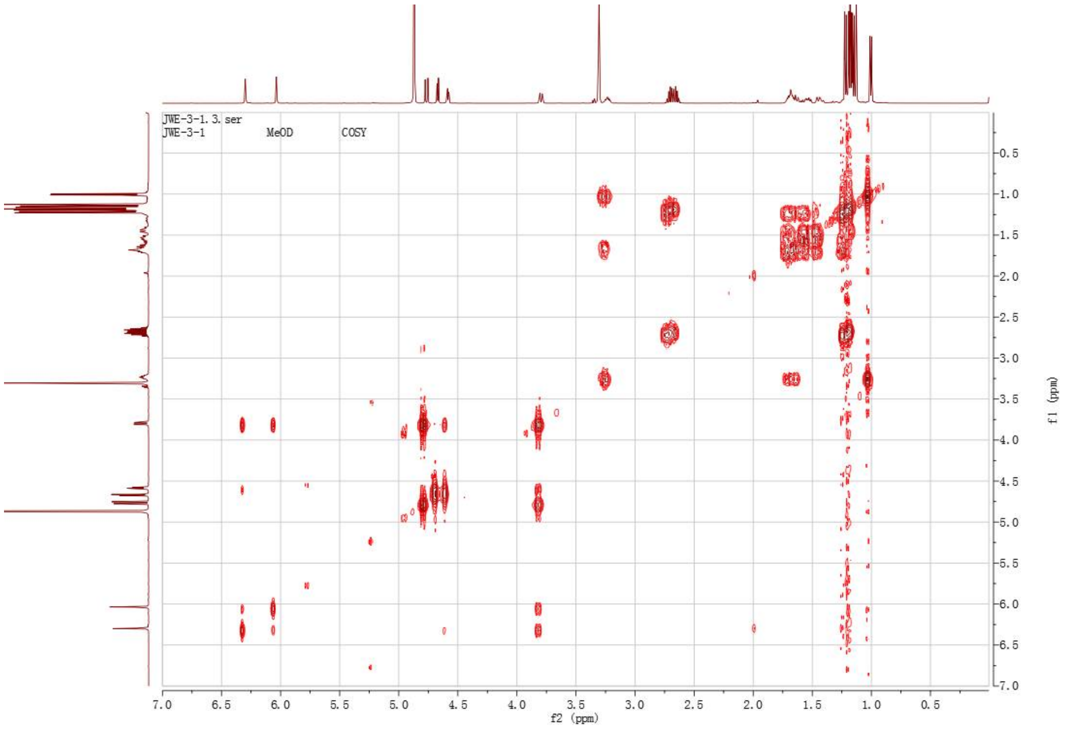

Fig. S1.3 ^{1}H-^{1}H COSY spectrum (500 MHz) of incaspitolide A (**1**) in CD_3OD

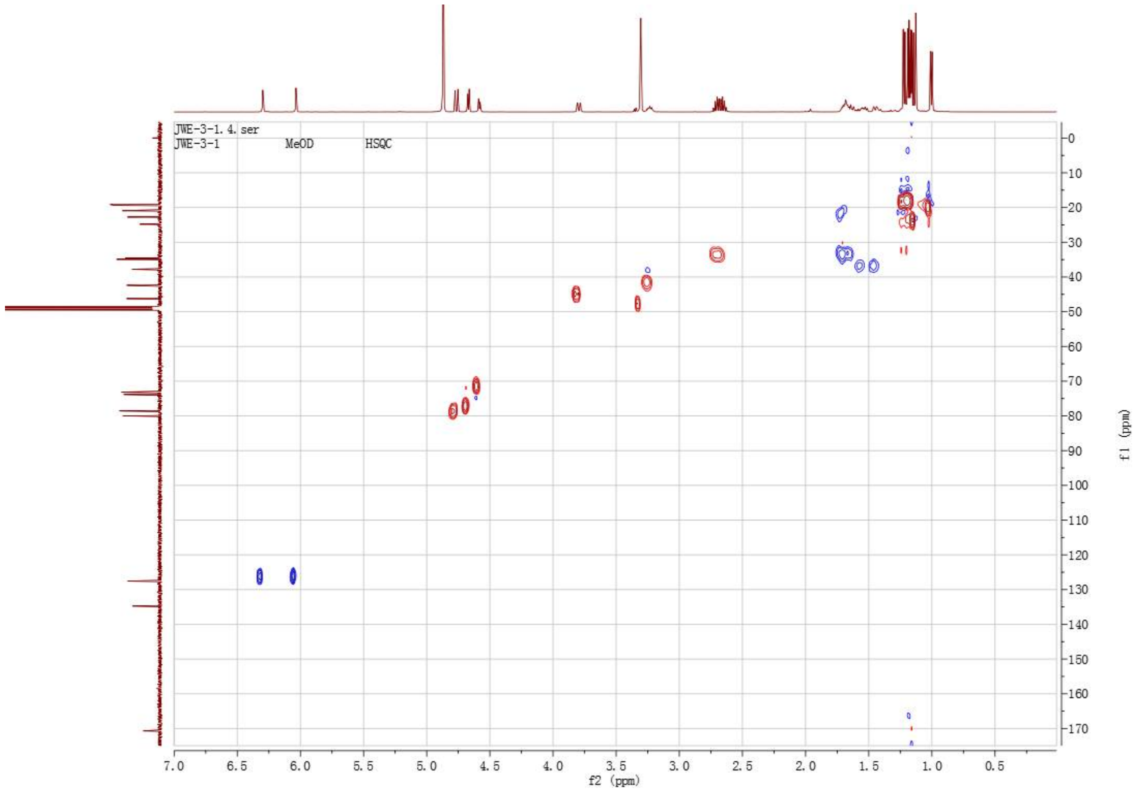

Fig. S1.4 HSQC spectrum (500 MHz) of incaspitolide A (**1**) in CD_3OD

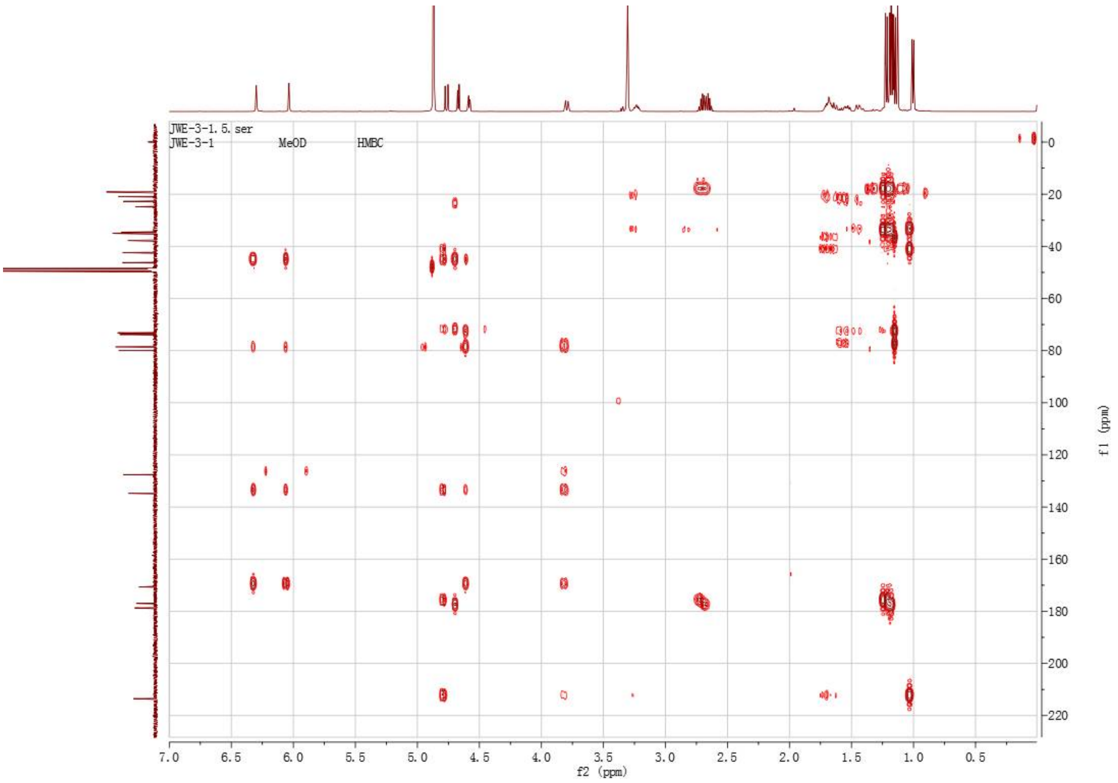

Fig. S1.5 HMBC spectrum (500 MHz) of incaspitolide A (**1**) in CD_3OD

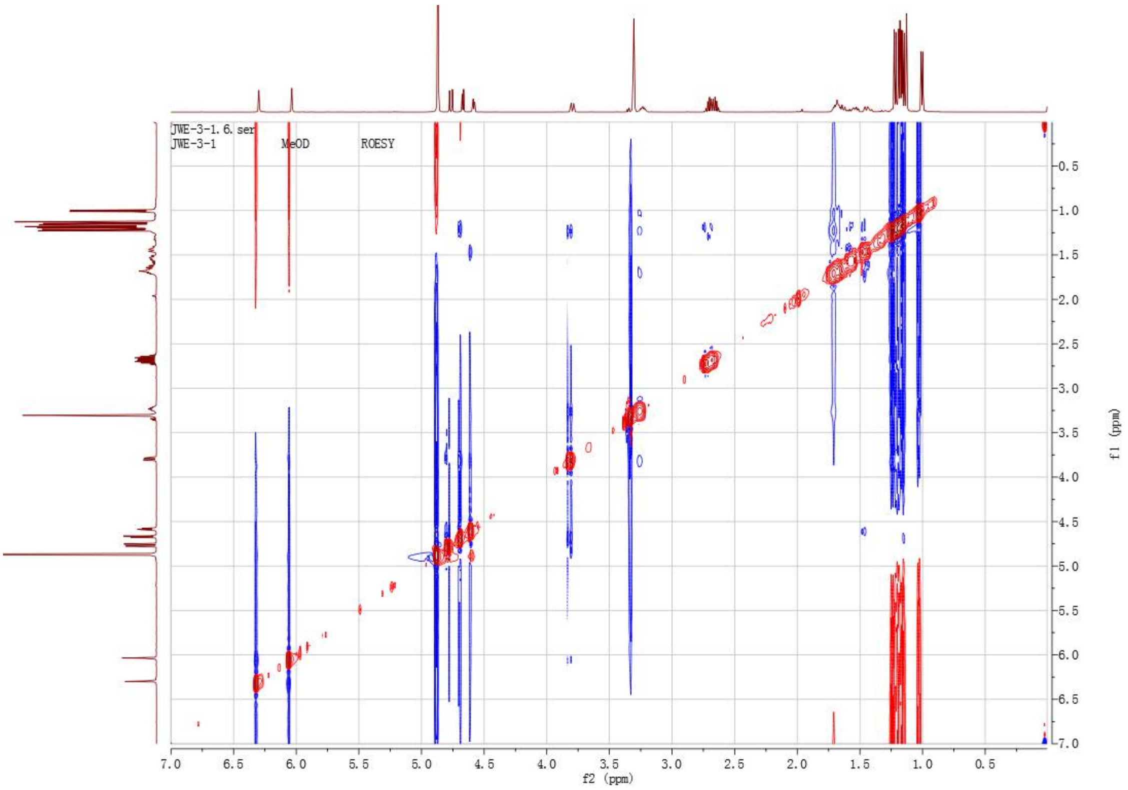

Fig. S1.6 ROESY spectrum (500 MHz) of incaspitolide A (**1**) in CD_3OD

Auto Print Report 1 2015-12-16 15:06:34

Data Set: Storage 150604 - RawData - D:\2015\File_151216_150604.spc
WJWE-3-1

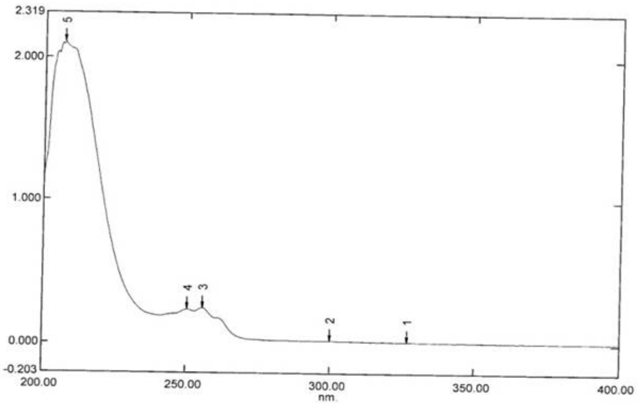

No.	P/V	Wavelength	Abs.	Description
1		327.00	0.015	
2		300.00	0.019	
3		255.50	0.243	
4		250.50	0.232	
5		207.00	2.109	

Fig. S1.7 UV spectrum of incaspitolide A (**1**)

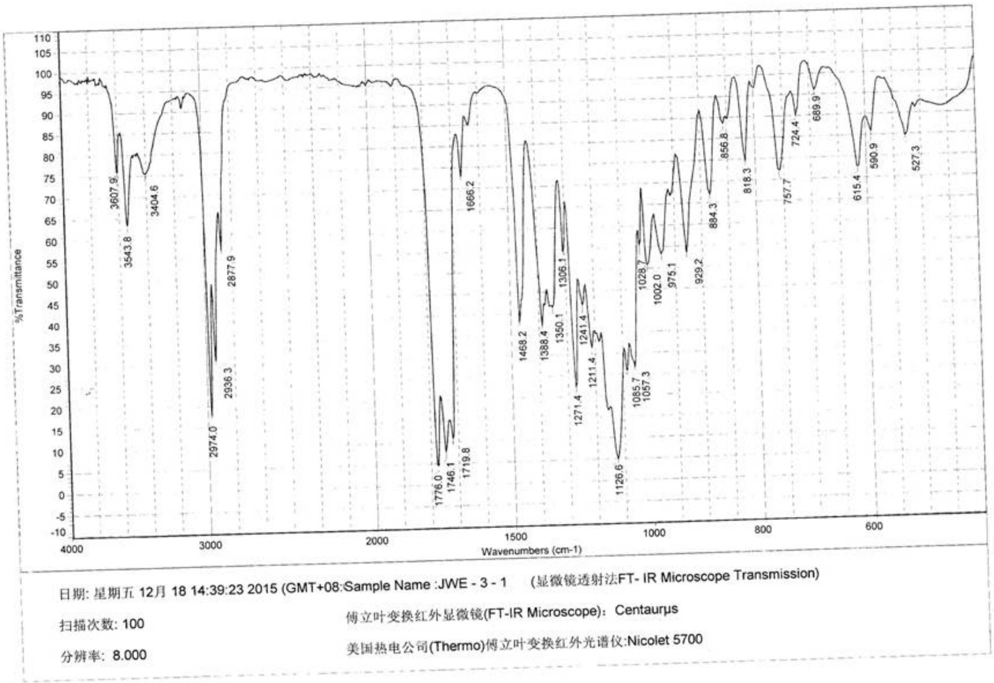

Fig. S1.8 IR spectrum of incaspitolide A (**1**)

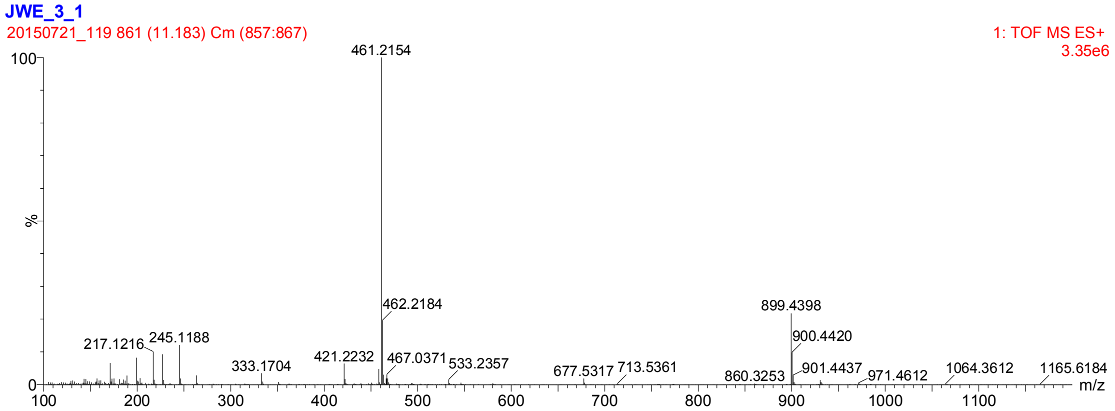

Fig. S1.9 HRESIMS spectrum of incaspitolide A (**1**)

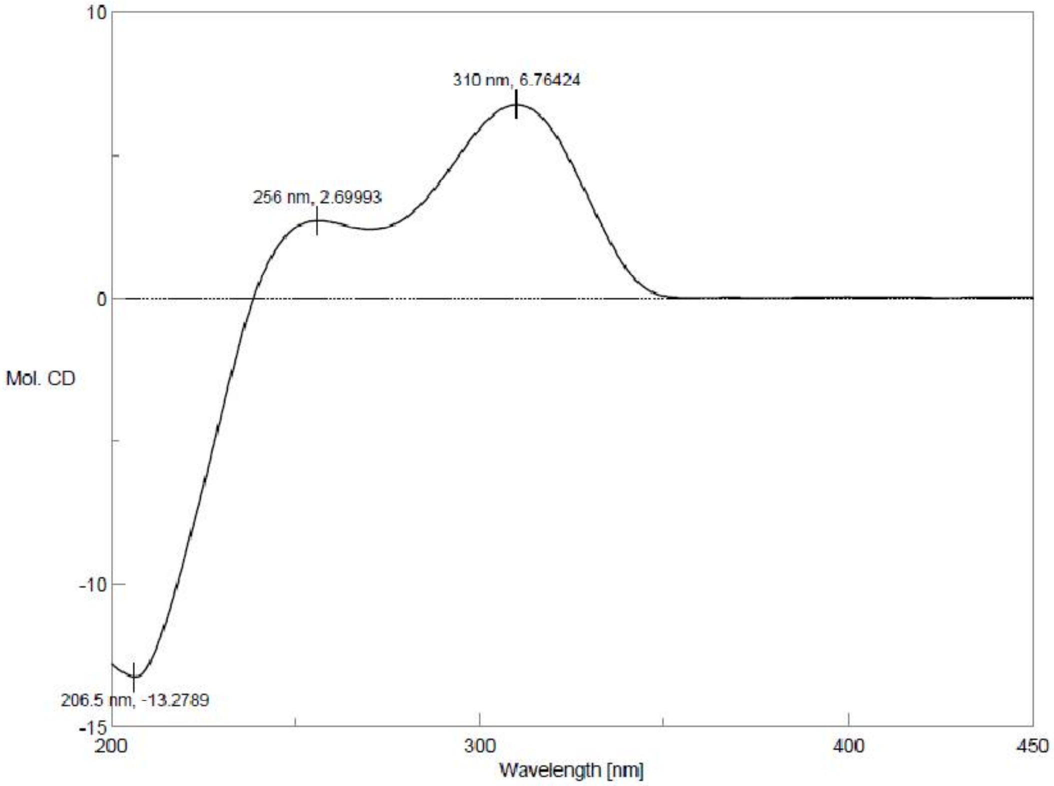

Fig. S1.10 CD spectrum of incaspitolide A (**1**)

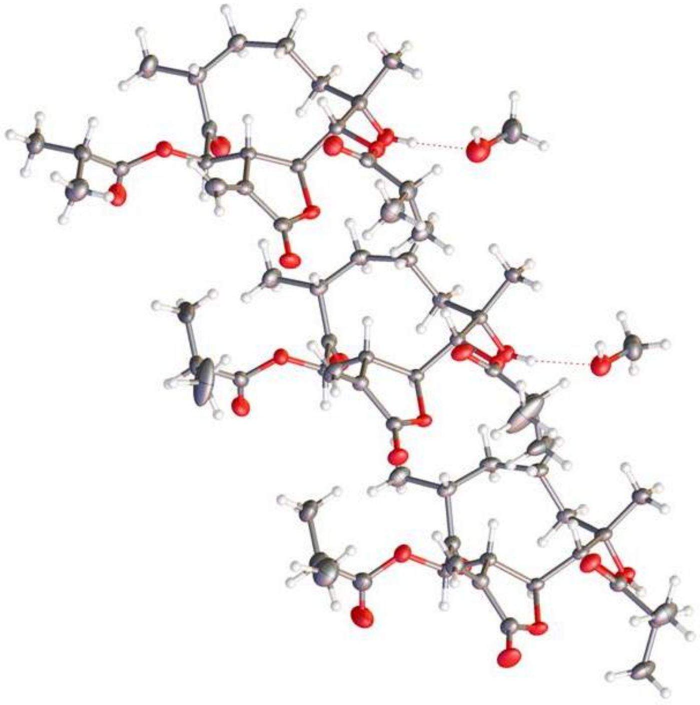

Fig. S1.11 X-ray ORTEP drawing of incaspitolide A (**1**)

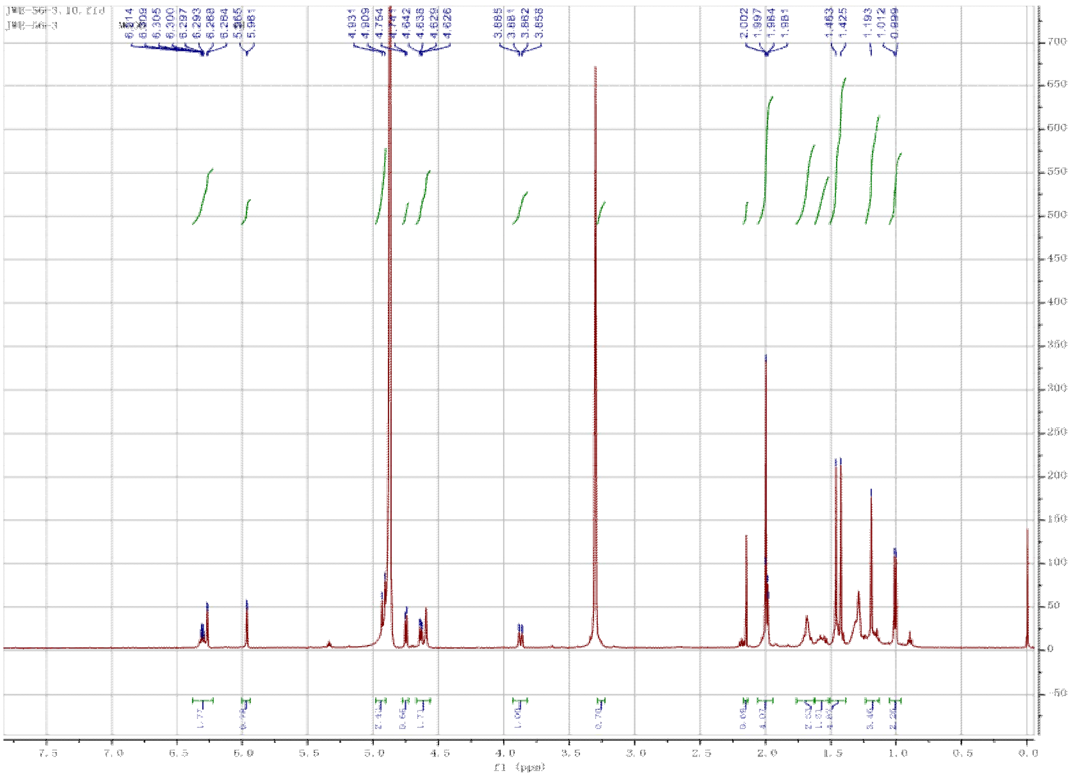

Fig. S2.1 ^{1}H NMR spectrum (500 MHz) of divarolide A (**2**) in CD_3OD

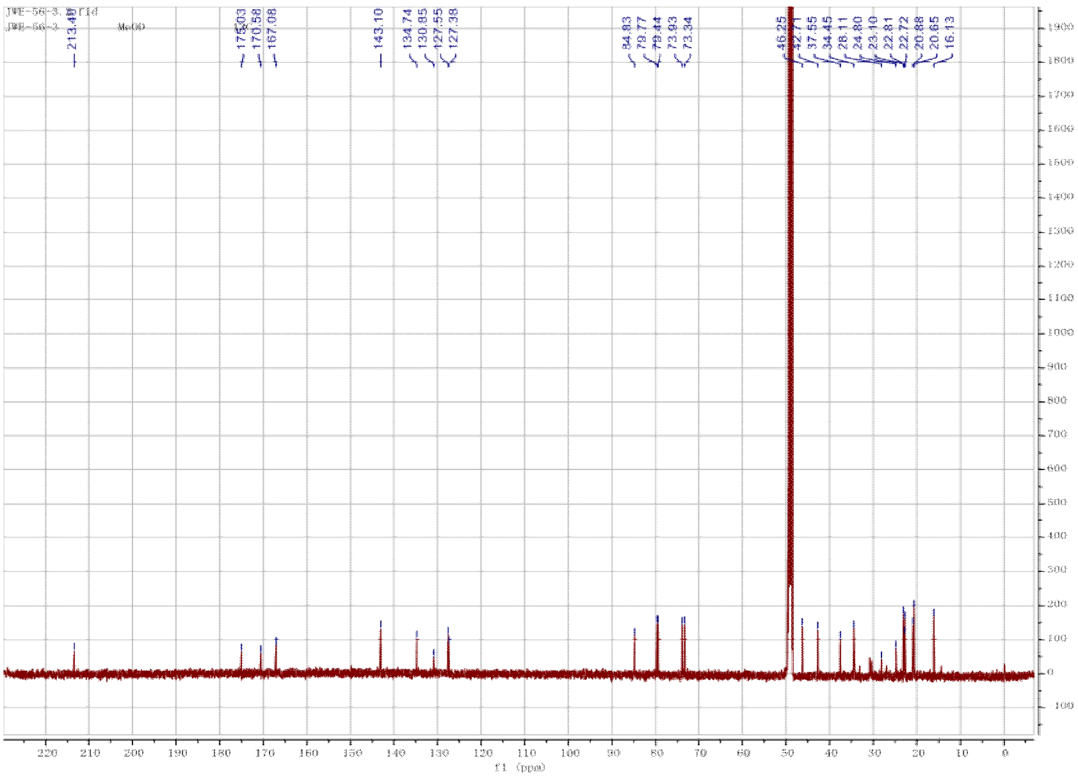

Fig. S2.2 ^{13}C NMR spectrum (500 MHz) of divarolide A (**2**) in CD_3OD

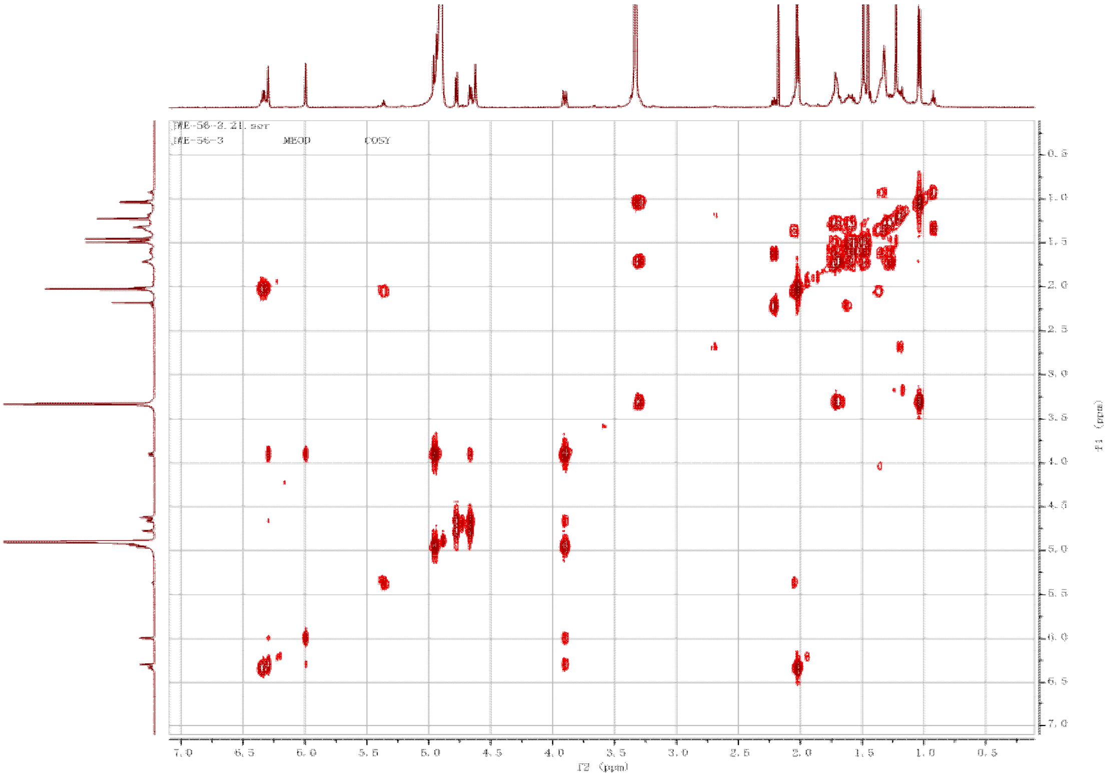

Fig. S2.3 ^{1}H-^{1}H COSY spectrum (500 MHz) of divarolide A (**2**) in CD_3OD

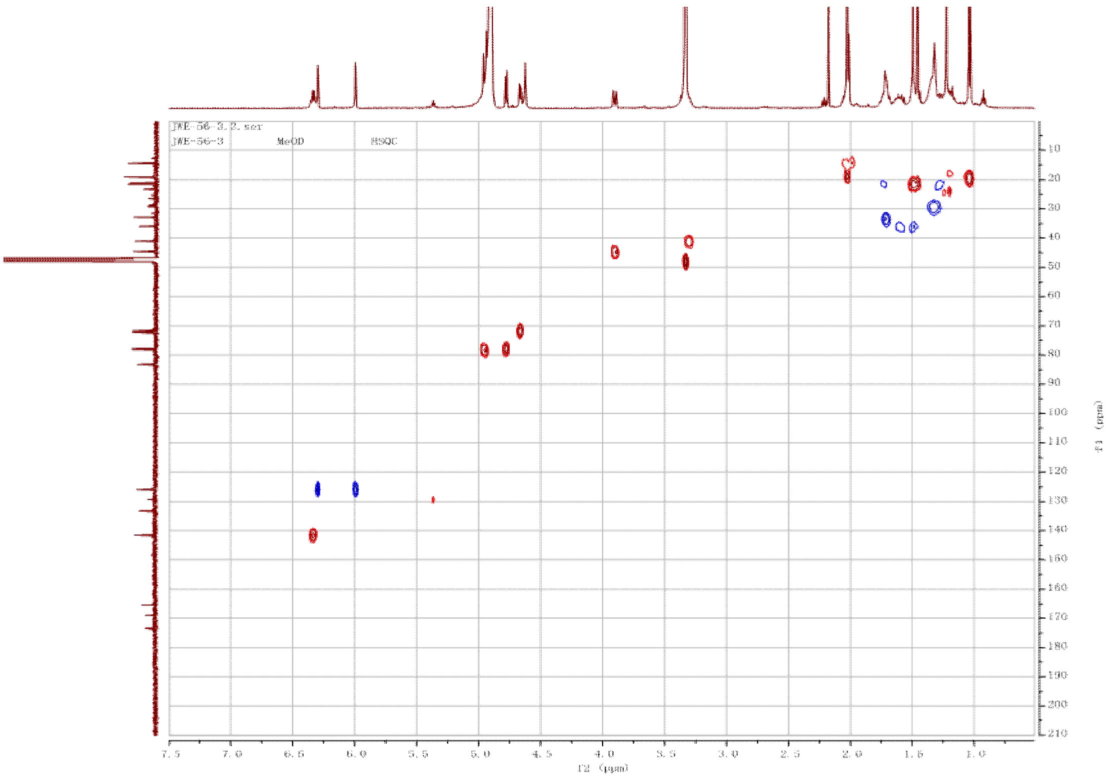

Fig. S2.4 HSQC spectrum (500 MHz) of divarolide A (**2**) in CD_3OD

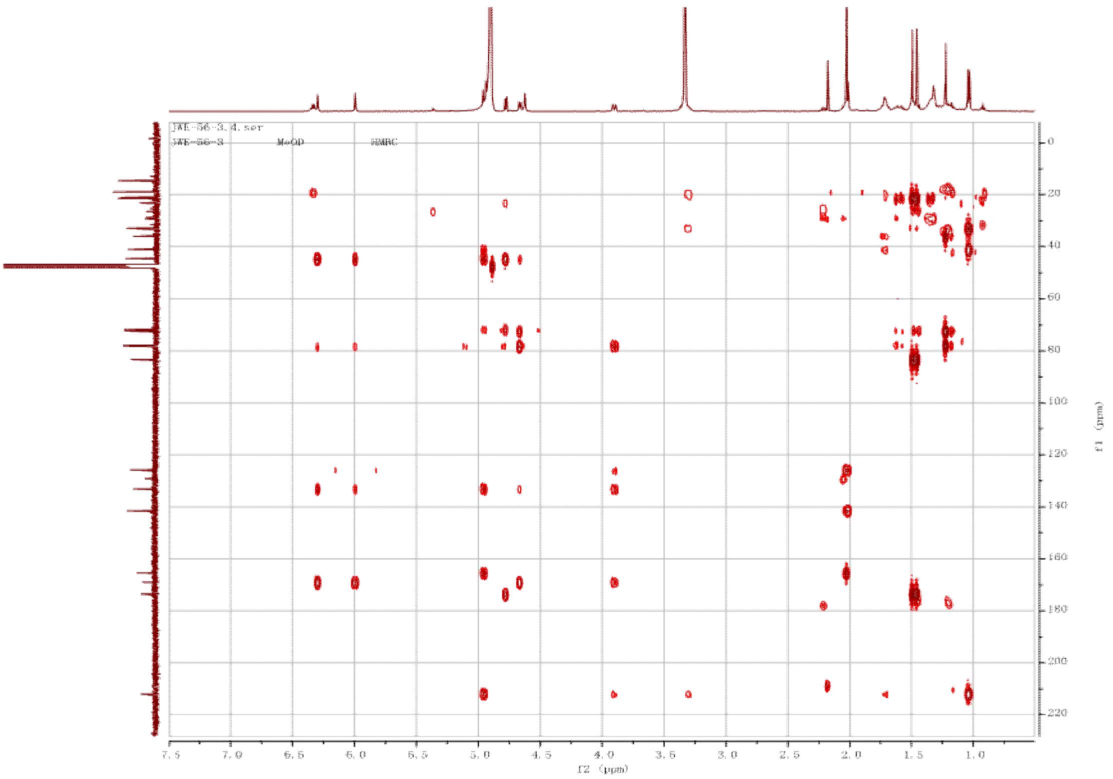

Fig. S2.5 HMBC spectrum (500 MHz) of divarolide A (**2**) in CD_3OD

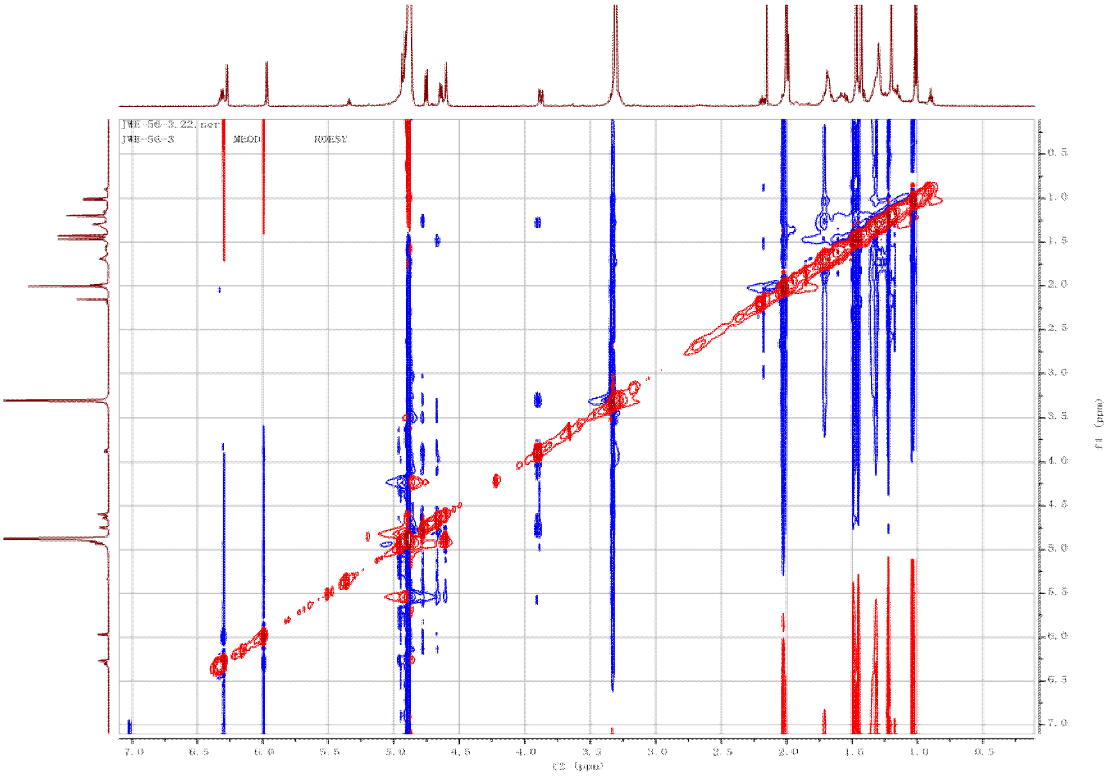

Fig. S2.6 ROESY spectrum (500 MHz) of divarolide A (**2**) in CD_3OD

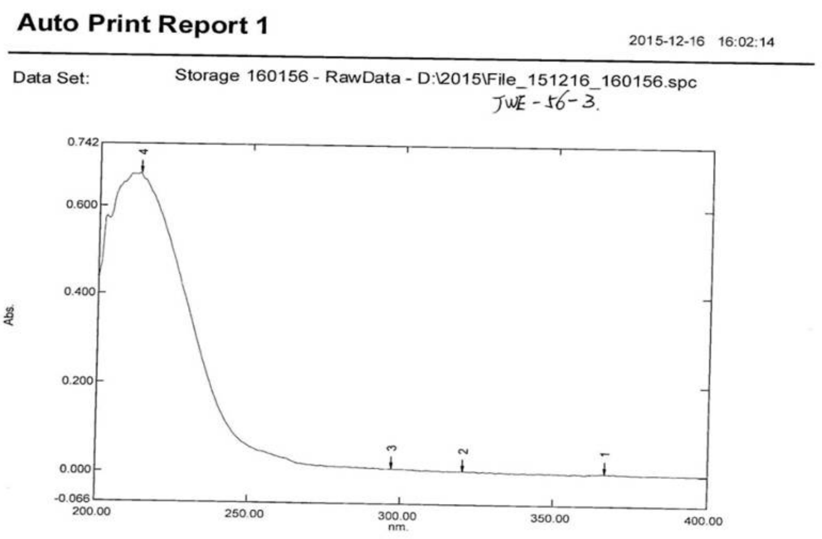

No.	P/V	Wavelength	Abs.	Description
1	⊕	366.50	0.004	
2	⊕	320.50	0.007	
3	⊕	297.00	0.011	
4	⊕	213.50	0.674	

Fig. S2.7 UV spectrum of divarolide A (**2**)

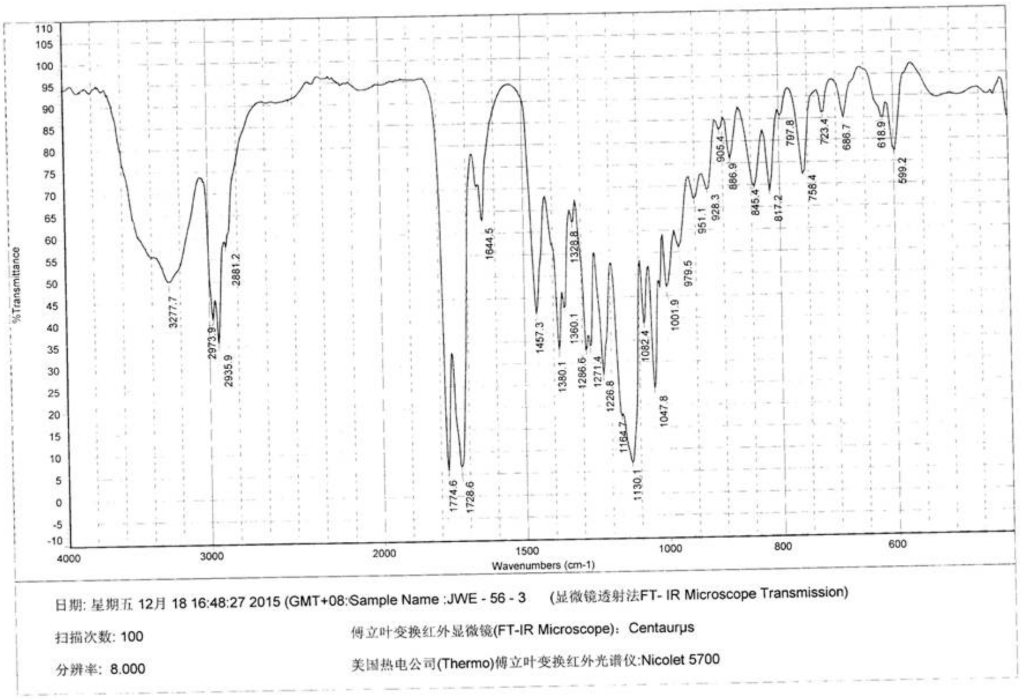

Fig. S2.8 IR spectrum of divarolide A (**2**)

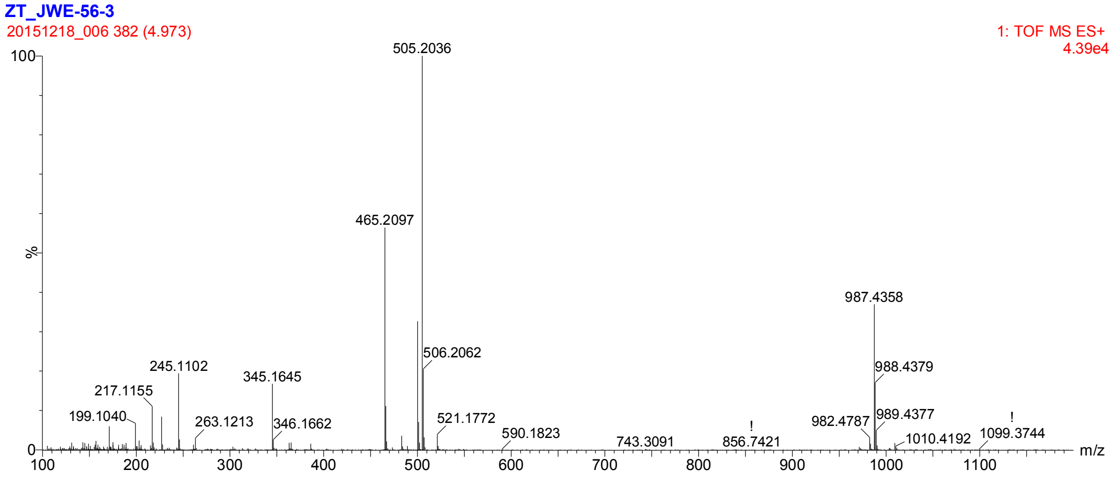

Fig. S2.9 HRESIMS spectrum of divarolide A (**2**)

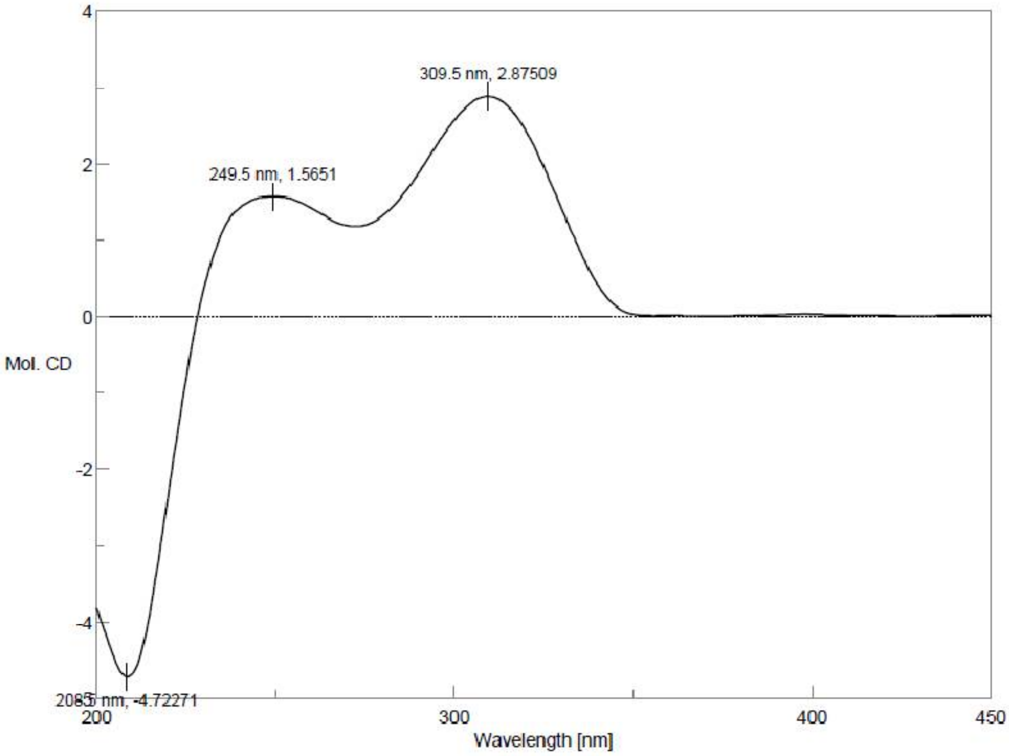

Fig. S2.10 CD spectrum of divarolide A (**2**)

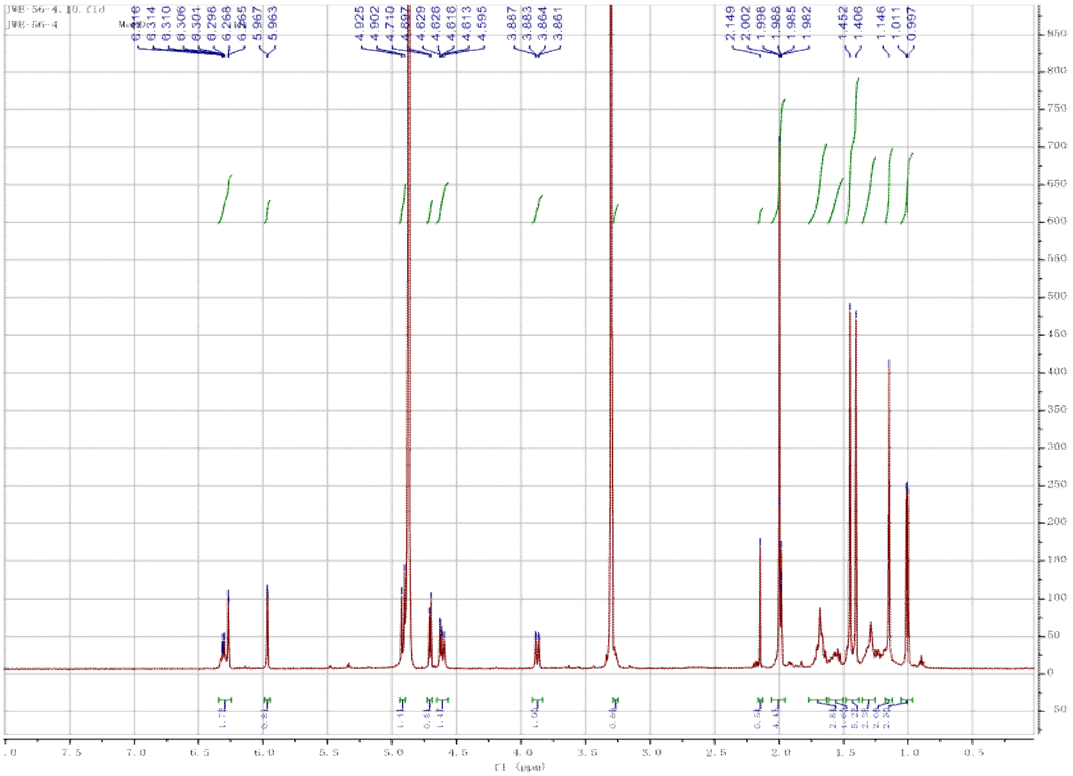

Fig. S3.1 ^{1}H NMR spectrum (500 MHz) of divarolide B (**3**) in CD_3OD

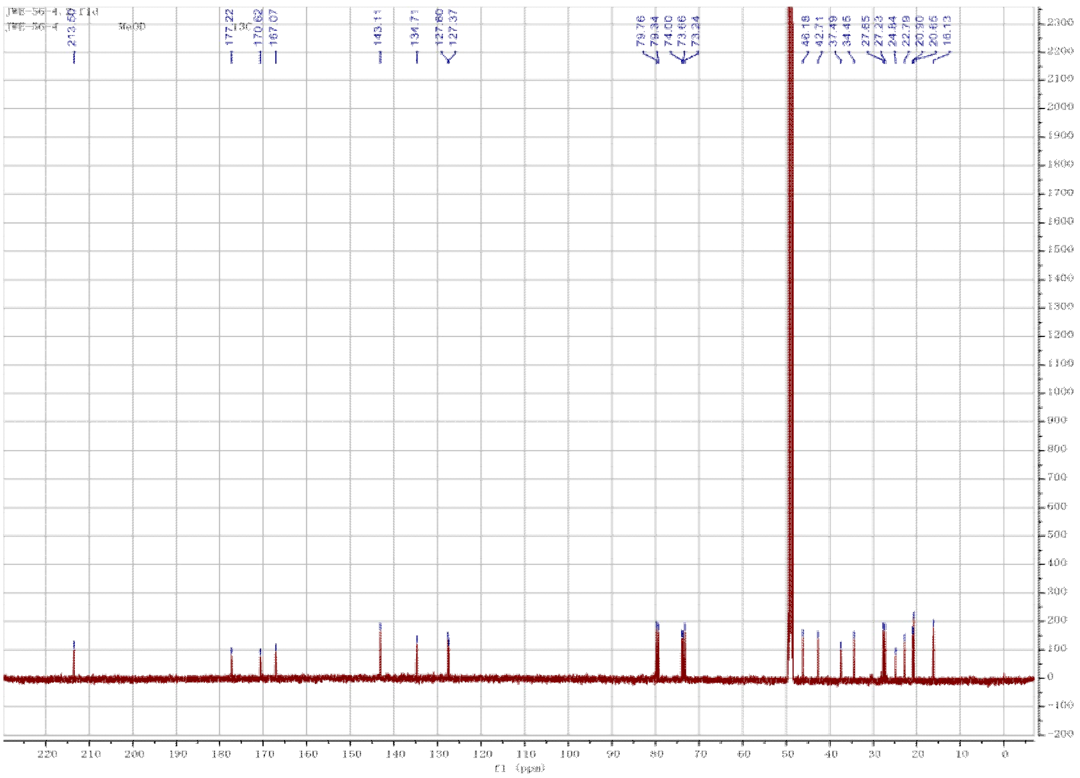

Fig. S3.2 ^{13}C NMR spectrum (500 MHz) of divarolide B (**3**) in CD_3OD

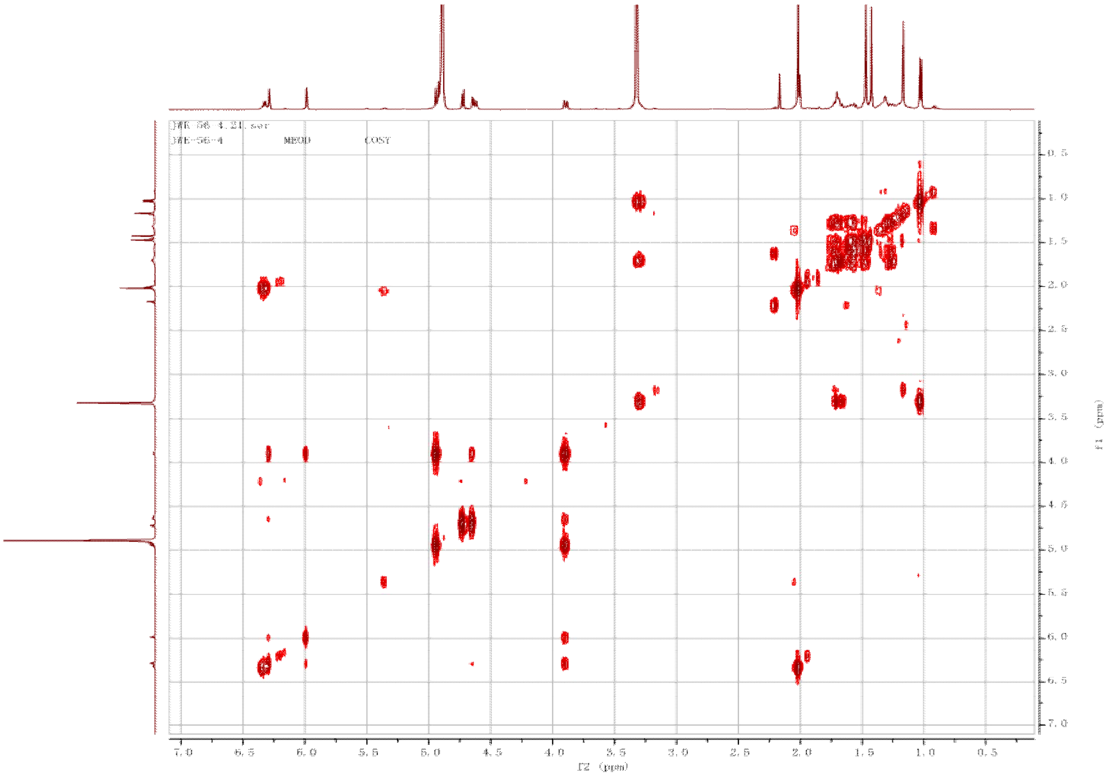

Fig. S3.3 ^{1}H-^{1}H COSY spectrum (500 MHz) of divarolide B (**3**) in CD_3OD

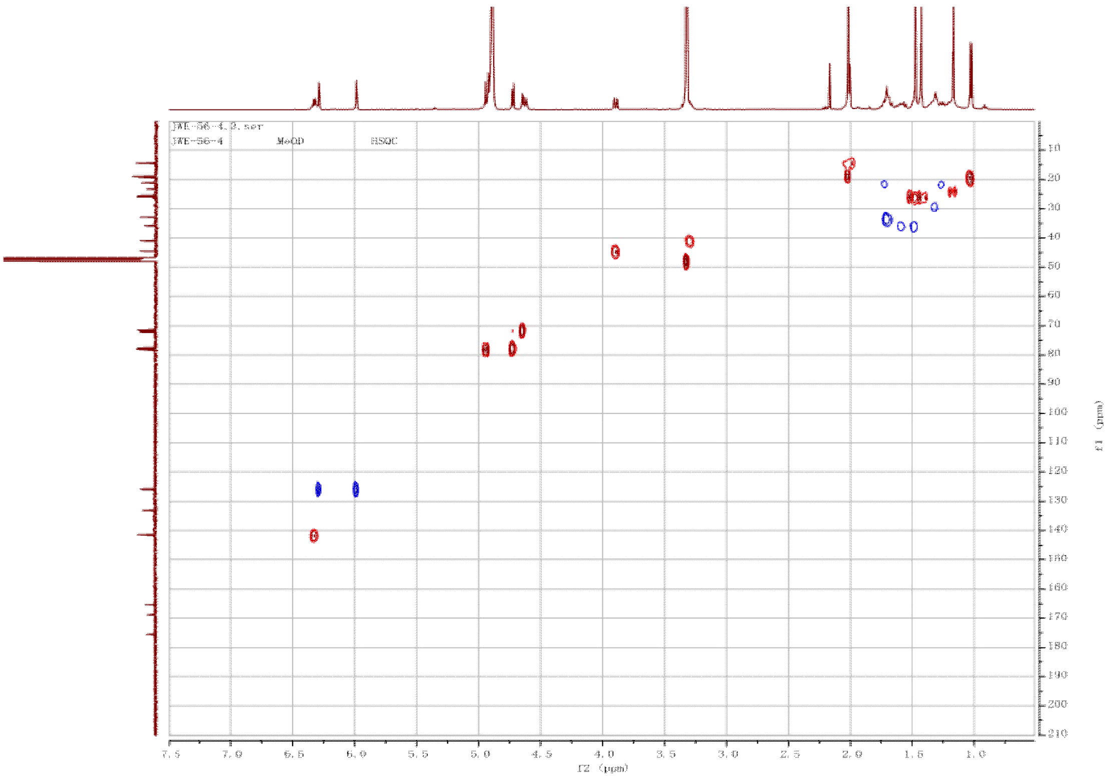

Fig. S3.4 HSQC spectrum (500 MHz) of divarolide B (**3**) in CD_3OD

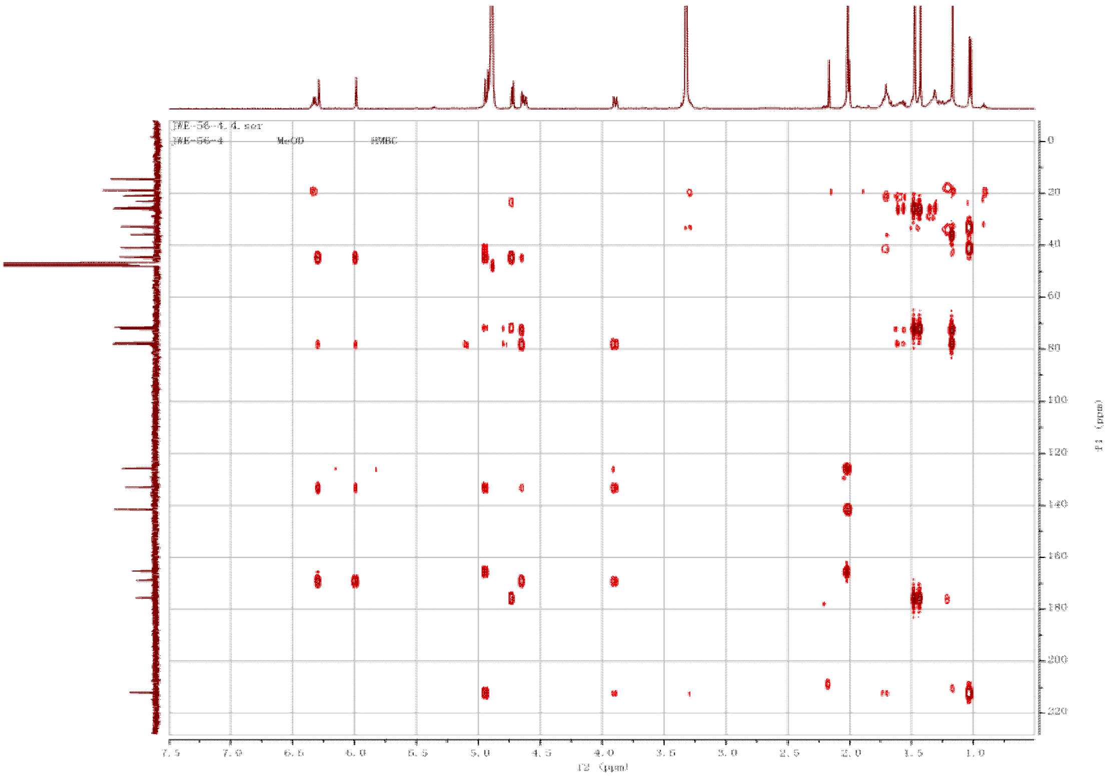

Fig. S3.5 HMBC spectrum (500 MHz) of divarolide B (**3**) in CD_3OD

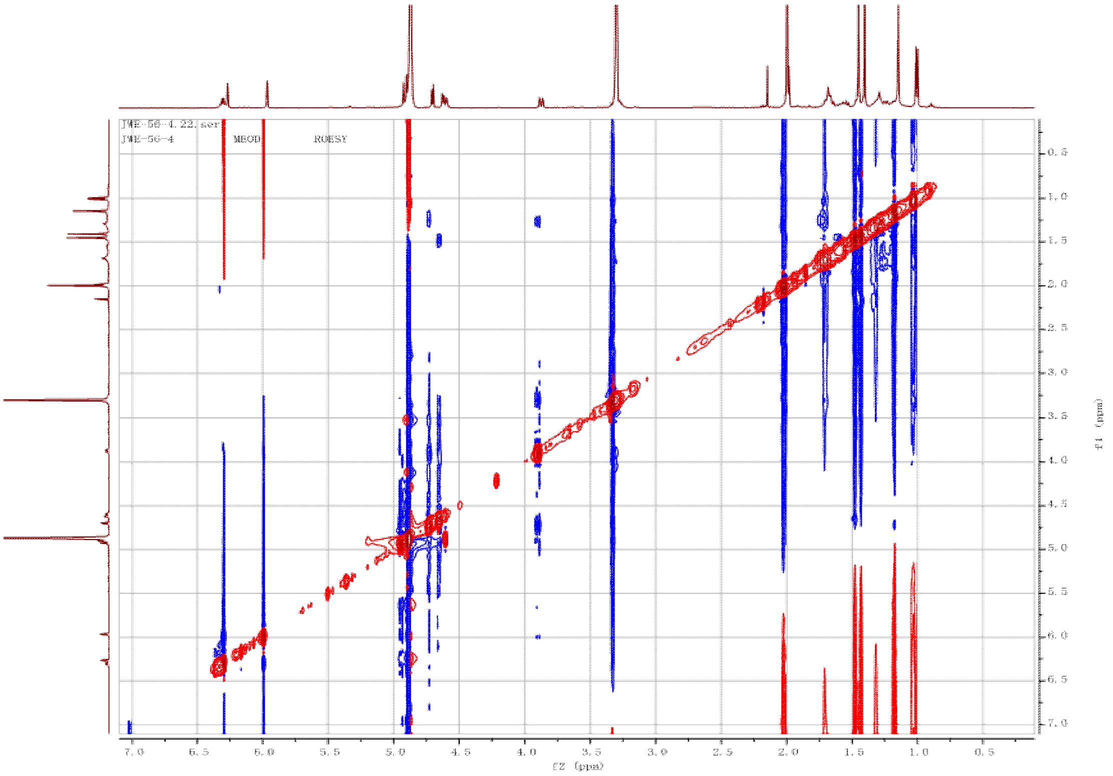

Fig. S3.6 ROESY spectrum (500 MHz) of divarolide B (**3**) in CD_3OD

Auto Print Report 1 2015-12-16 16:18:18

Data Set: Storage 161515 - RawData - D:\2015\JWE-56-4.spc

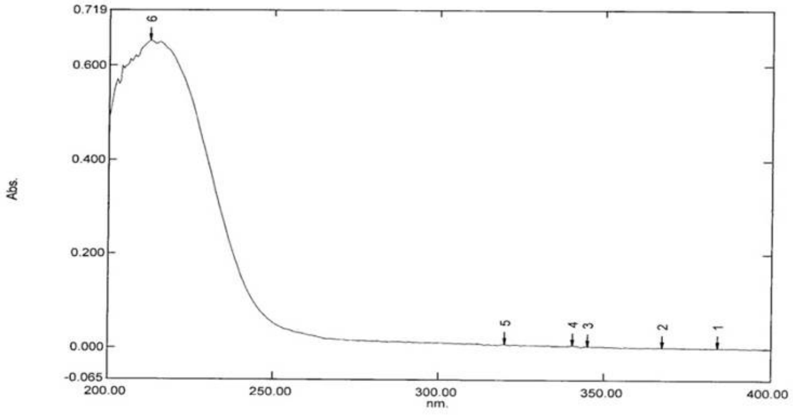

No.	P/V	Wavelength	Abs.	Description
1	⊕	384.00	0.002	
2	⊕	367.50	0.003	
3	⊕	345.00	0.005	
4	⊕	340.50	0.006	
5	⊕	320.00	0.009	
6	⊕	212.50	0.654	

Fig. S3.7 UV spectrum of divarolide B (**3**)

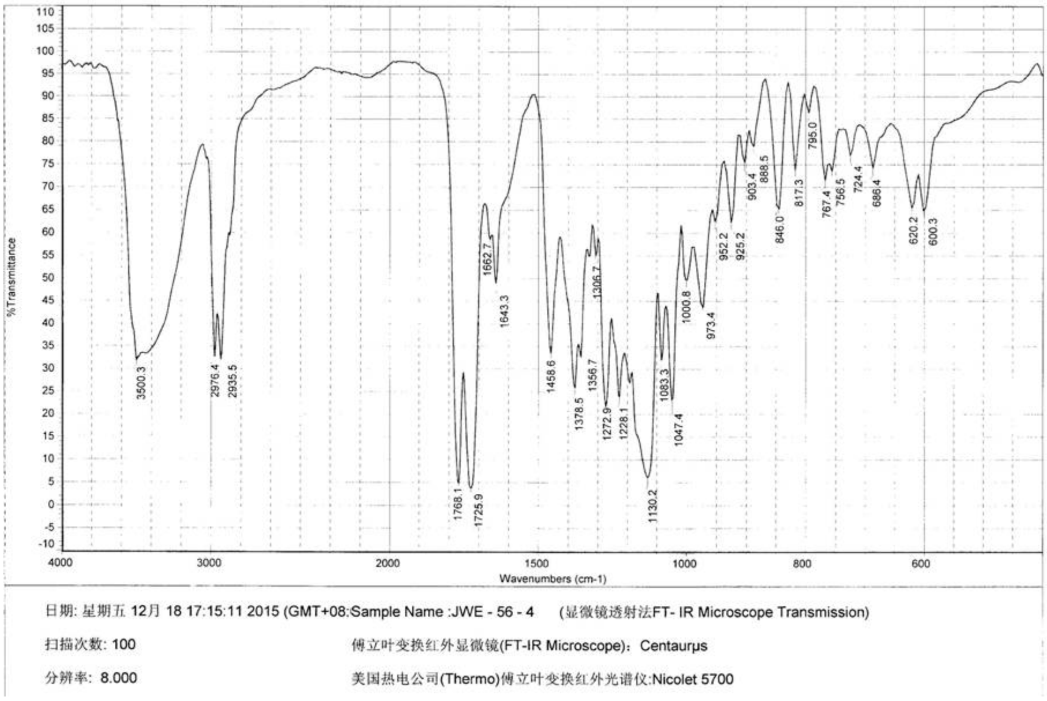

Fig. S3.8 IR spectrum of divarolide B (**3**)

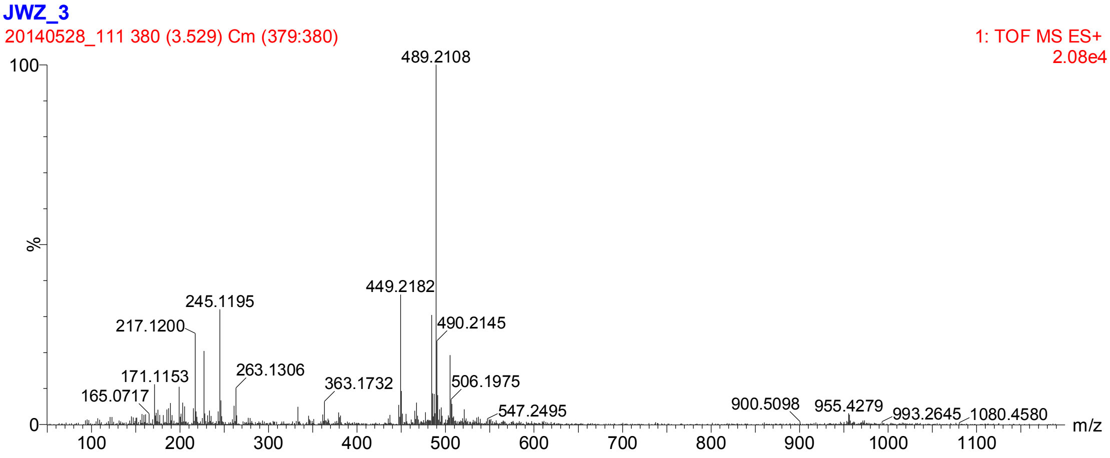

Fig. S3.9 HRESIMS spectrum of divarolide B (**3**)

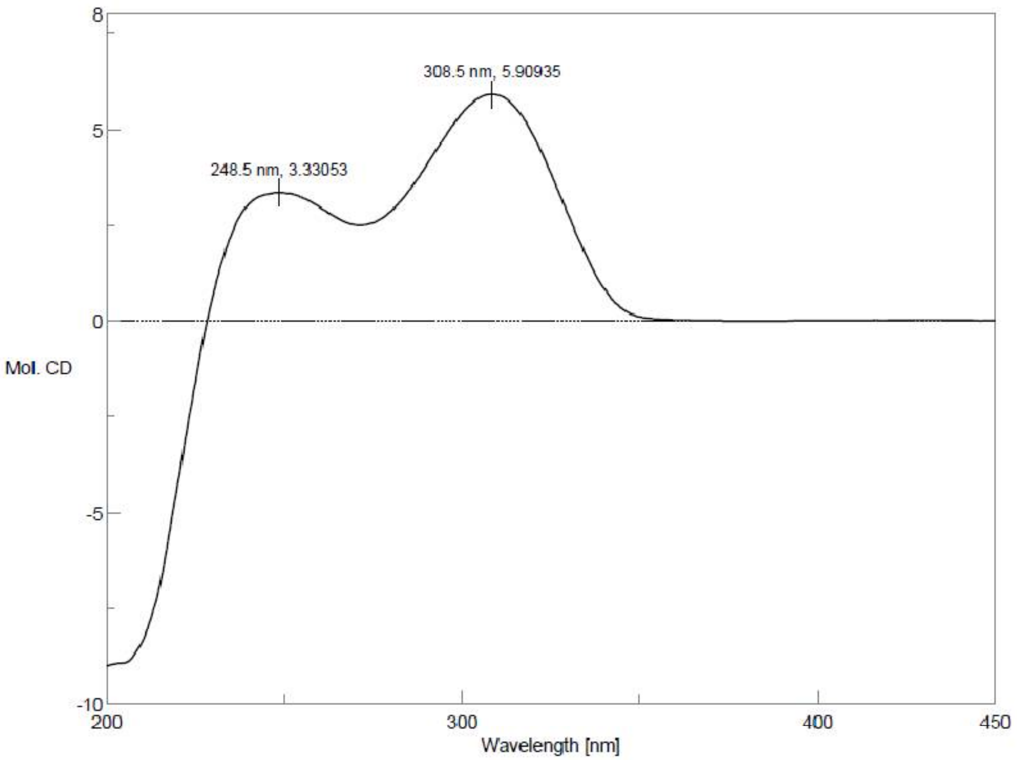

Fig. S3.10 CD spectrum of divarolide B (**3**)

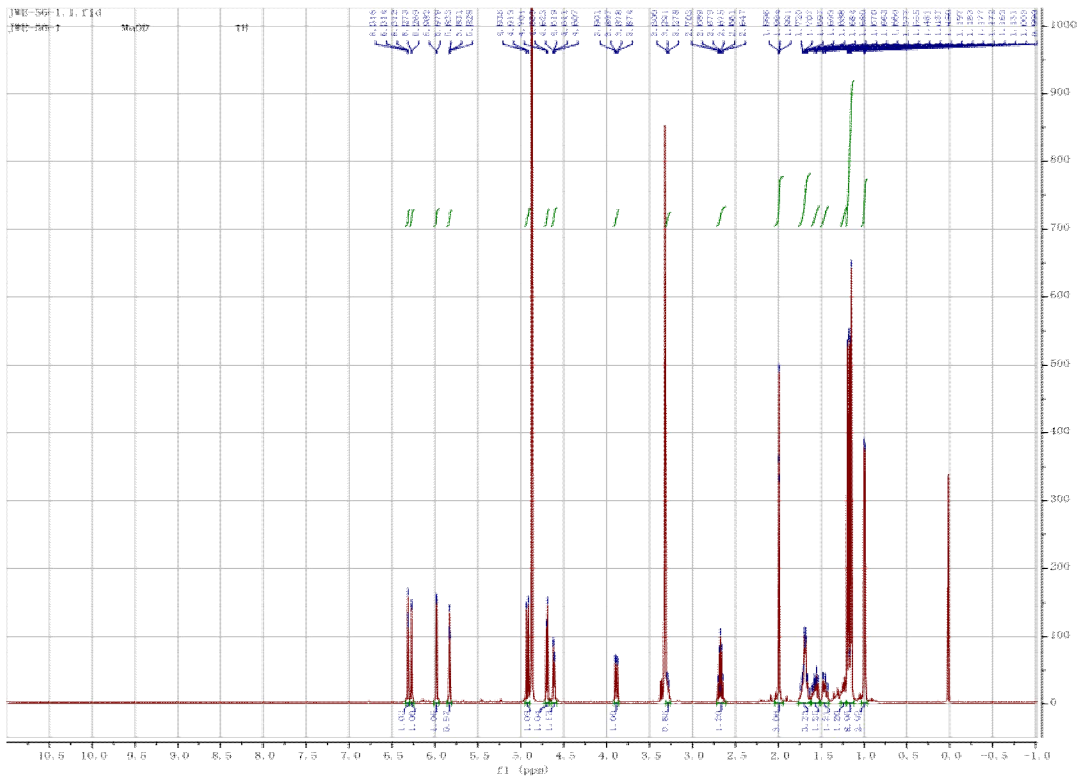

Fig. S4.1 ^{1}H NMR spectrum (500 MHz) of divarolide C (**4**) in CD_3OD

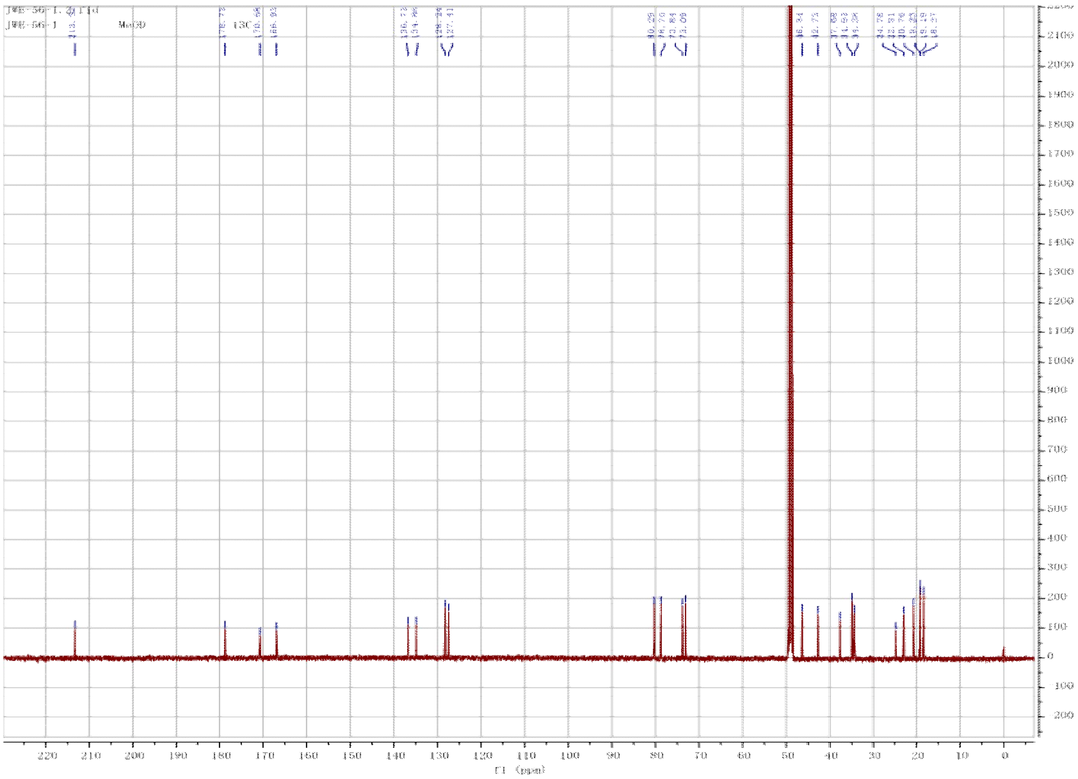

Fig. S4.2 ^{13}C NMR spectrum (500 MHz) of divarolide C (**4**) in CD_3OD

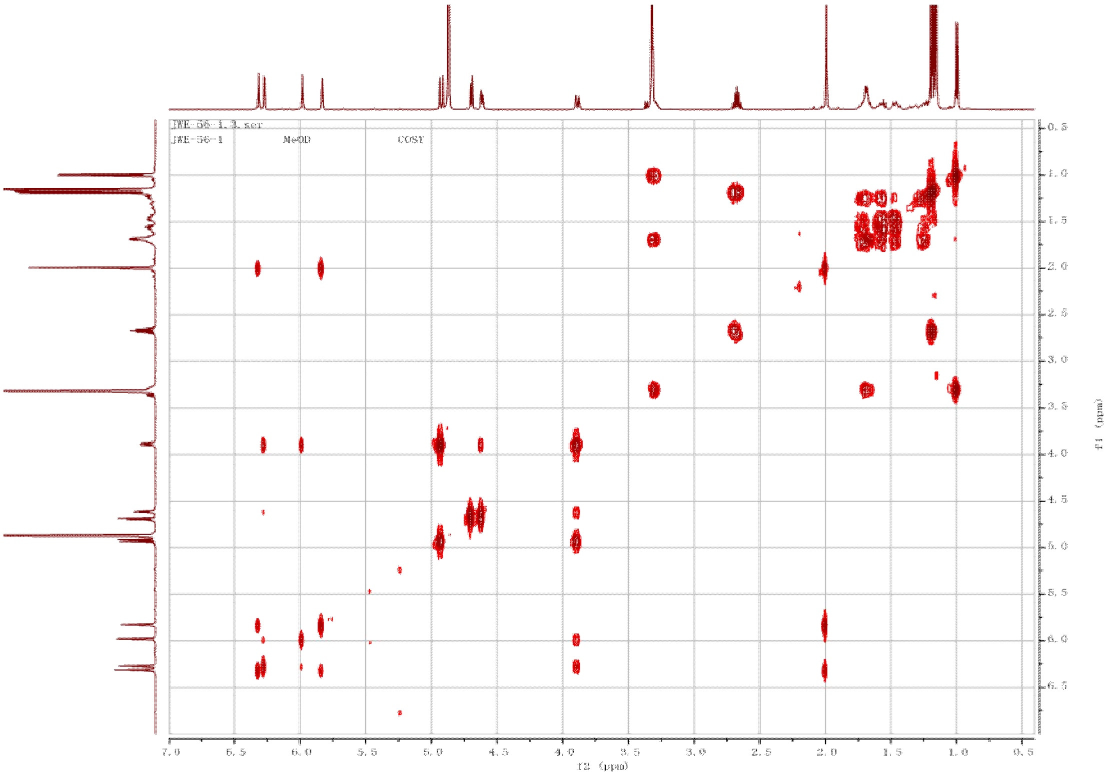

Fig. S4.3 ^{1}H-^{1}H COSY spectrum (500 MHz) of divarolide C (**4**) in CD_3OD

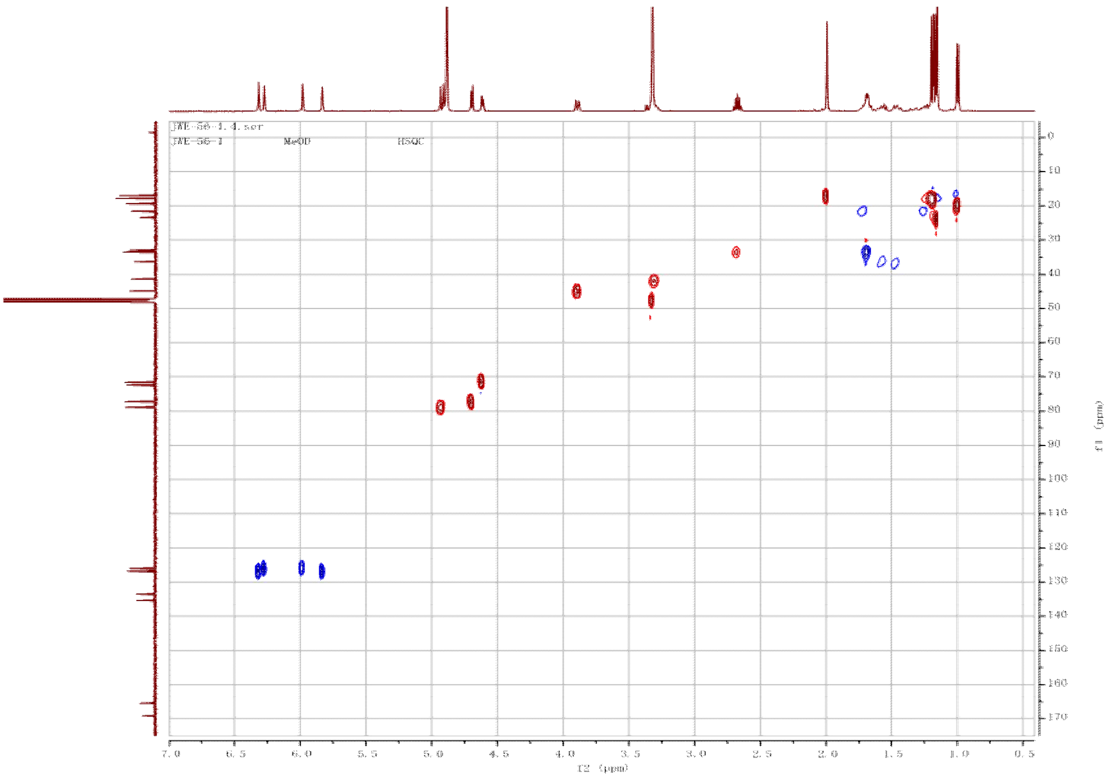

Fig. S4.4 HSQC spectrum (500 MHz) of divarolide C (**4**) in CD_3OD

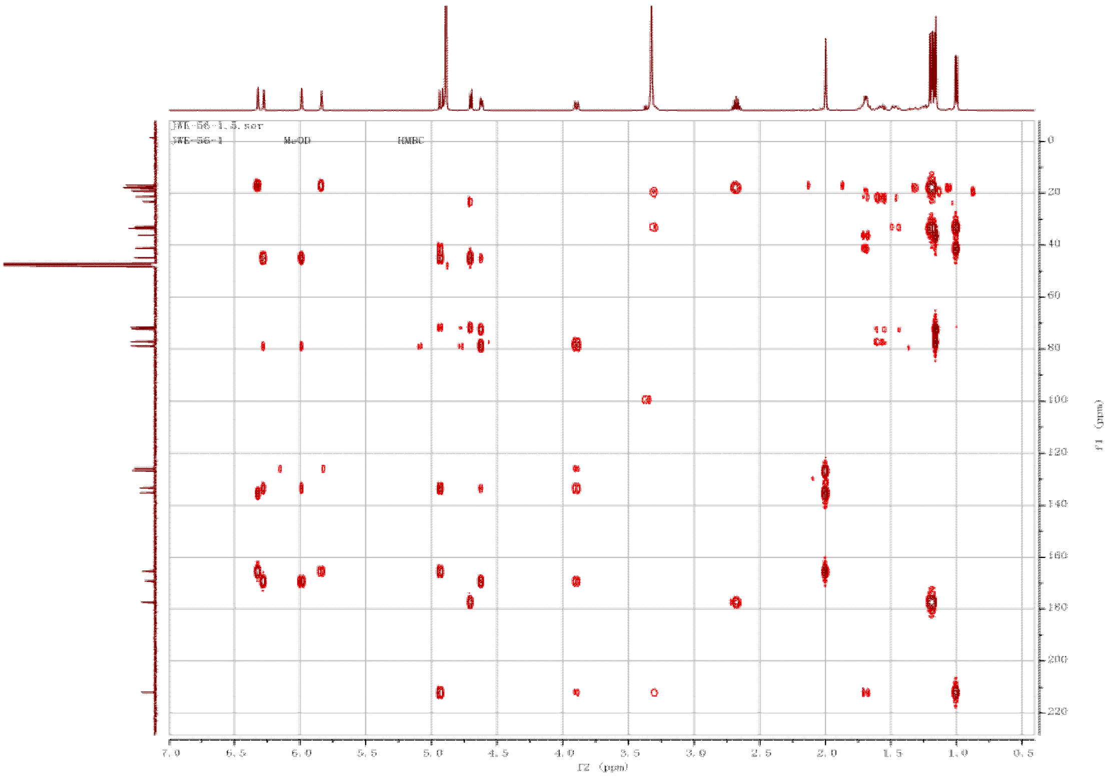

Fig. S4.5 HMBC spectrum (500 MHz) of divarolide C (**4**) in CD_3OD

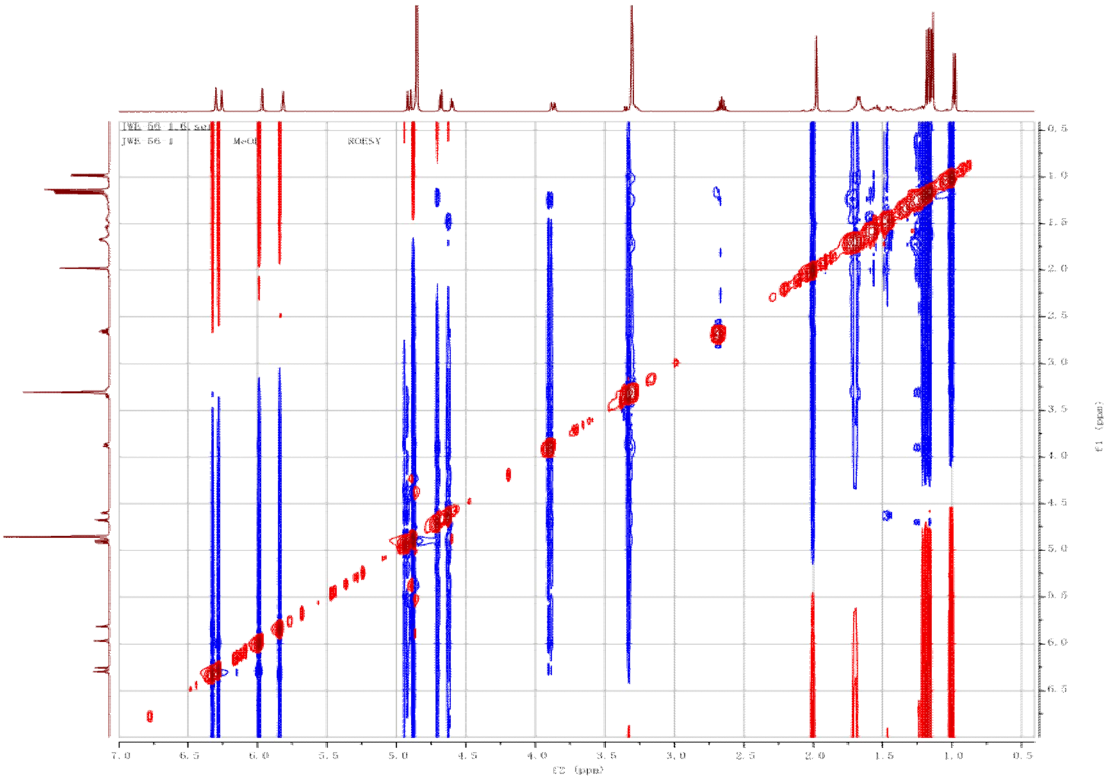

Fig. S4.6 ROESY spectrum (500 MHz) of divarolide C (**4**) in CD_3OD

Auto Print Report 1

2015-12-16 15:04:15

Data Set: Storage 150311 - RawData - D:\2015\JWE-56-1.spc

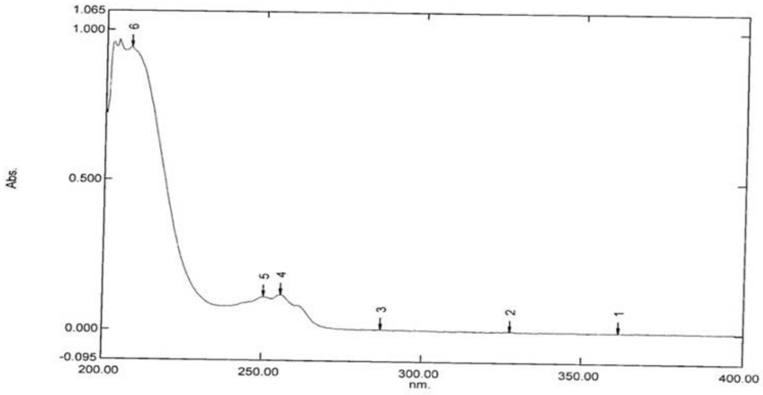

No.	P/V	Wavelength	Abs.	Description
1	↑	361.50	0.004	
2	↑	327.50	0.006	
3	↑	287.00	0.007	
4	↑	255.50	0.119	
5	↑	250.50	0.111	
6	↑	208.00	0.944	

Fig. S4.7 UV spectrum of divarolide C (**4**)

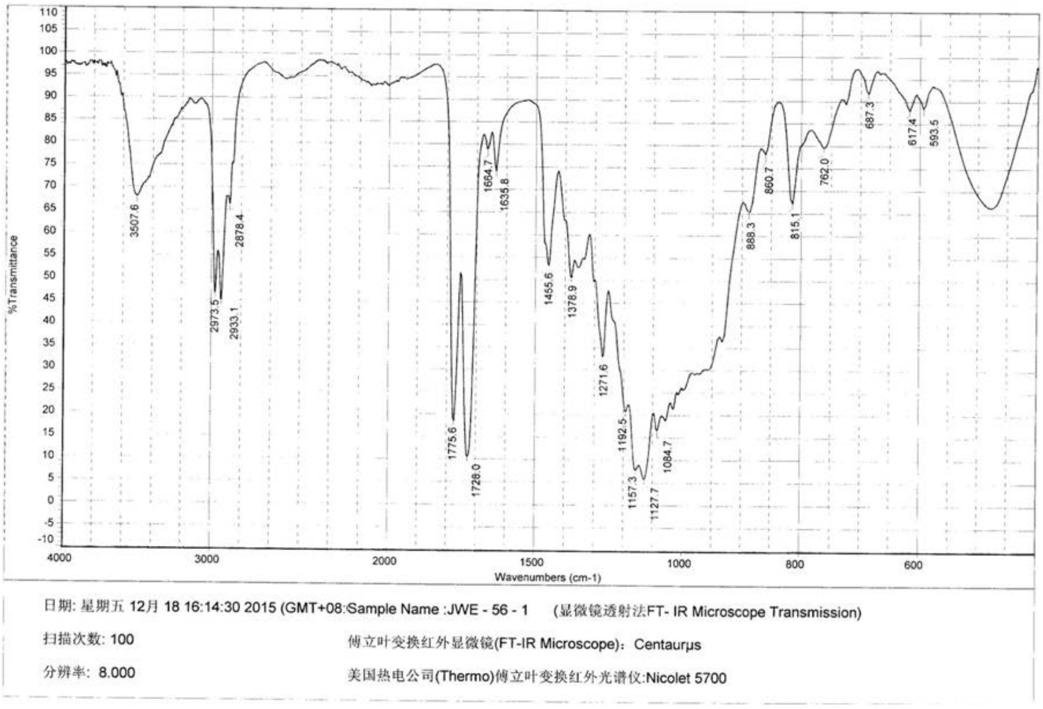

Fig. S4.8 IR spectrum of divarolide C (**4**)

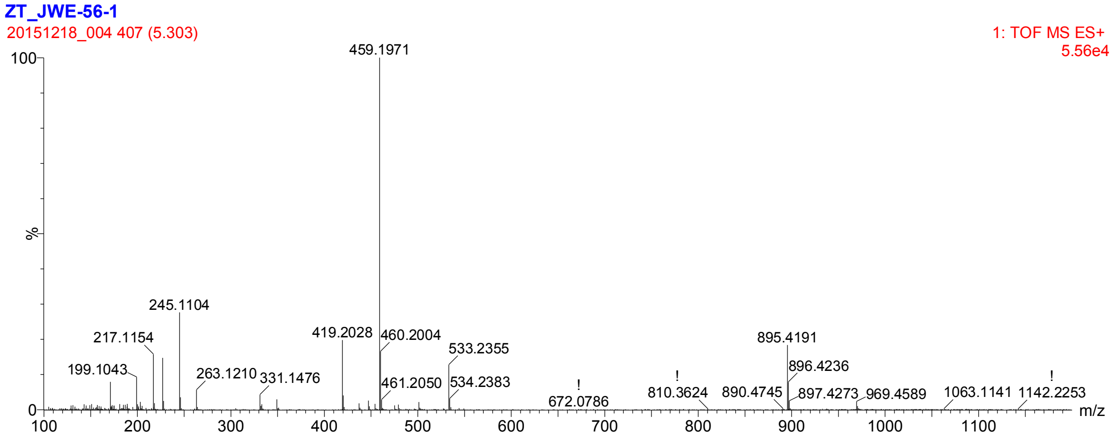

Fig. S4.9 HRESIMS spectrum of divarolide C (**4**)

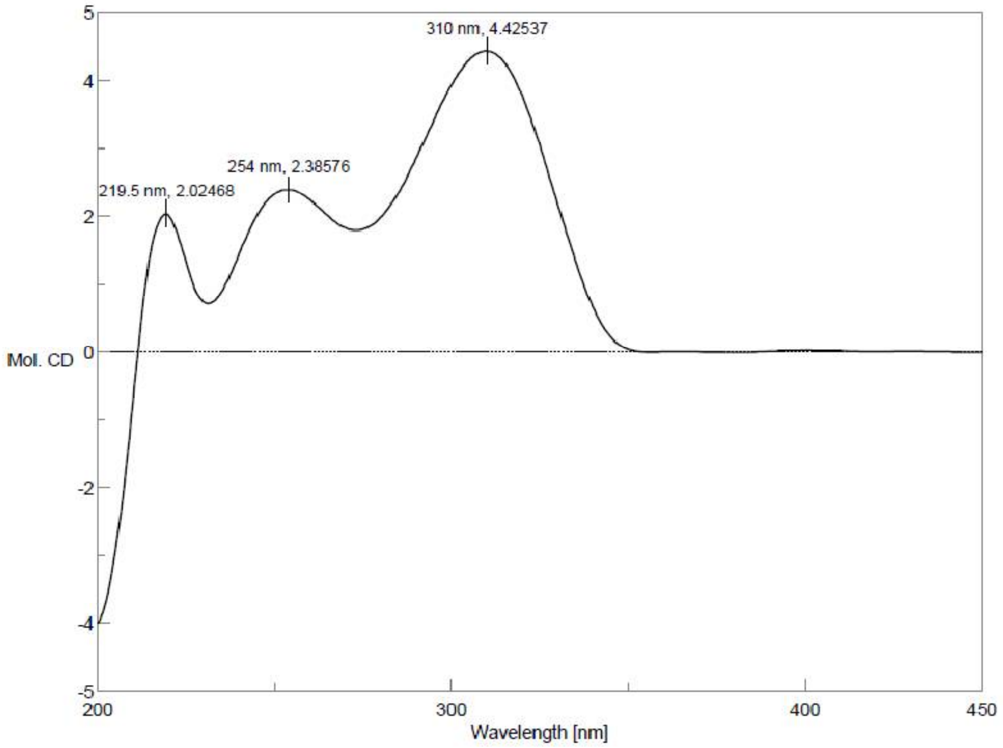

Fig. S4.10 CD spectrum of divarolide C (**4**)

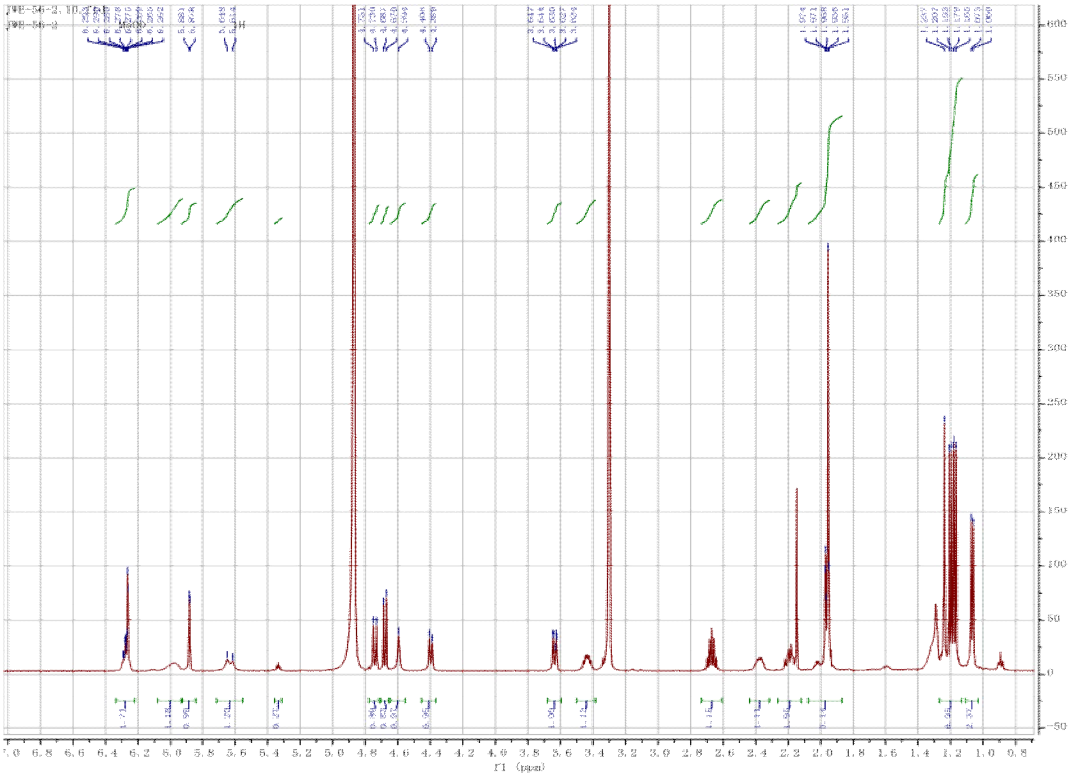

Fig. S5.1 ^{1}H NMR spectrum (500 MHz) of divarolide D (**5**) in CD_3OD

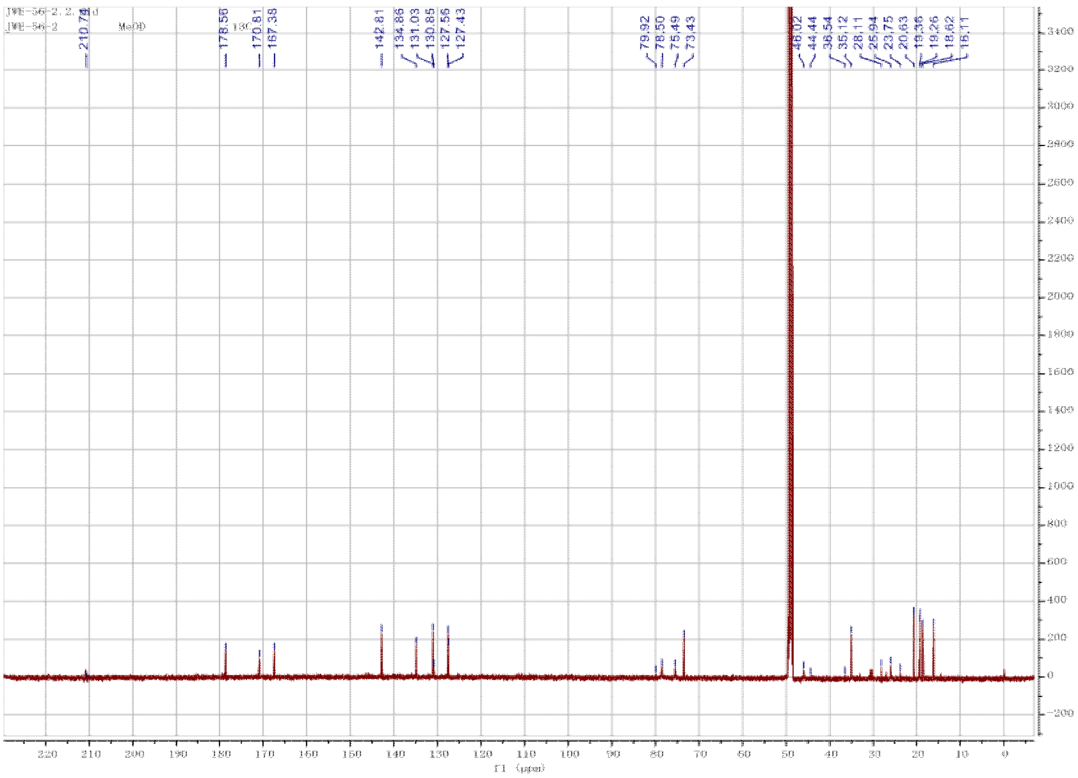

Fig. S5.2 ^{13}C NMR spectrum (500 MHz) of divarolide D (**5**) in CD_3OD

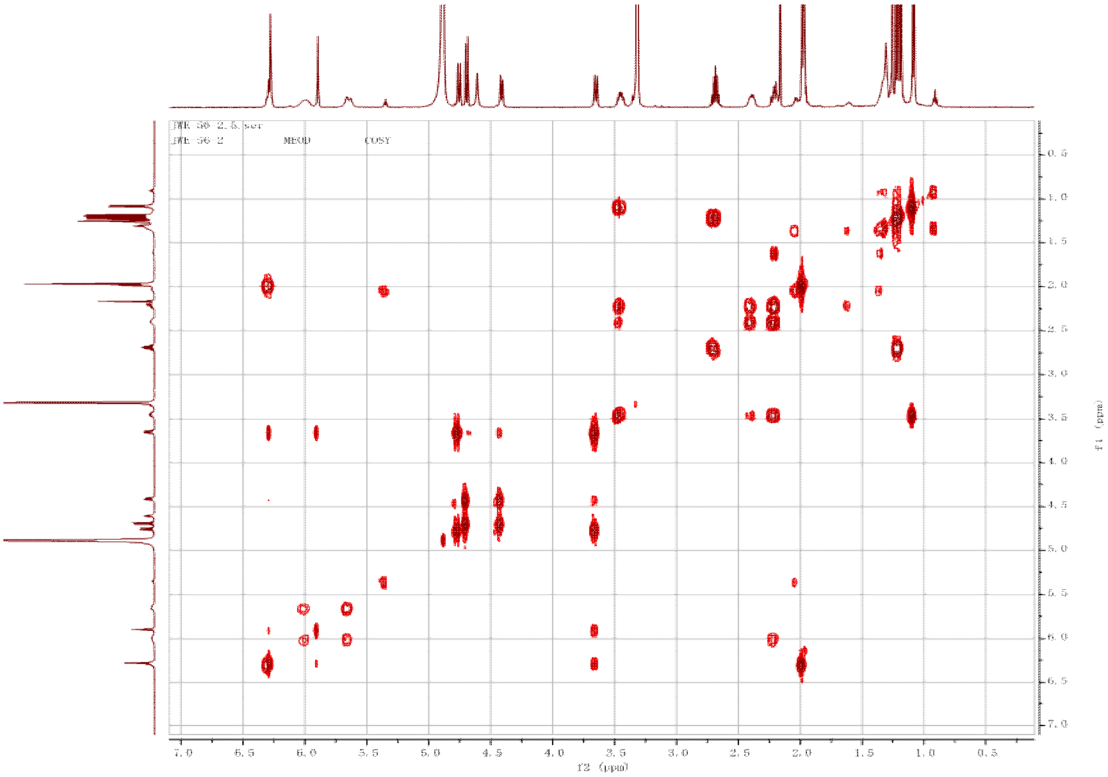

Fig. S5.3 ^{1}H-^{1}H COSY spectrum (500 MHz) of divarolide D (**5**) in CD_3OD

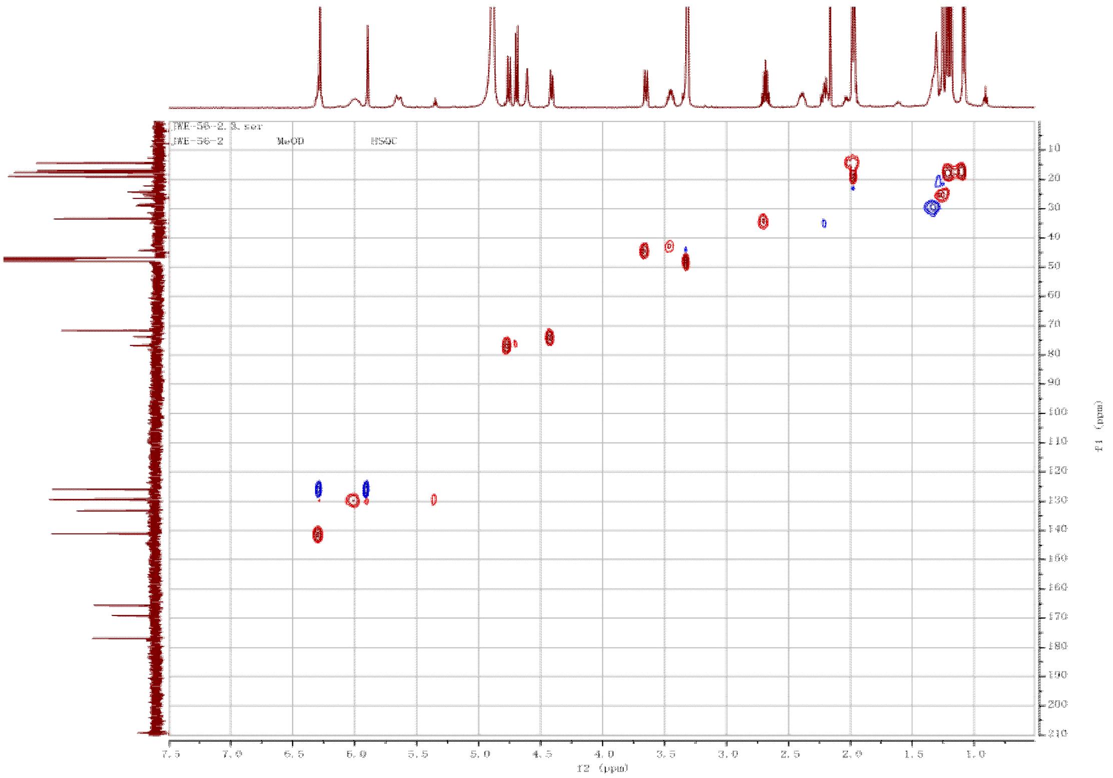

Fig. S5.4 HSQC spectrum (500 MHz) of divarolide D (**5**) in CD_3OD

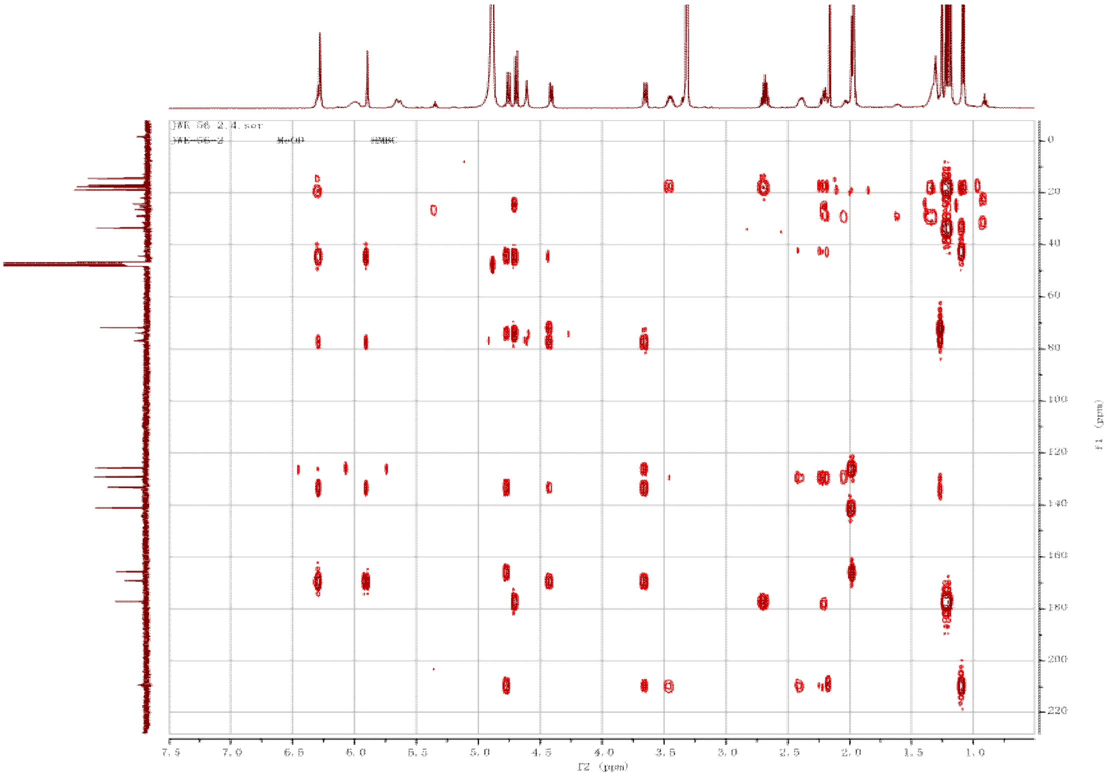

Fig. S5.5 HMBC spectrum (500 MHz) of divarolide D (**5**) in CD_3OD

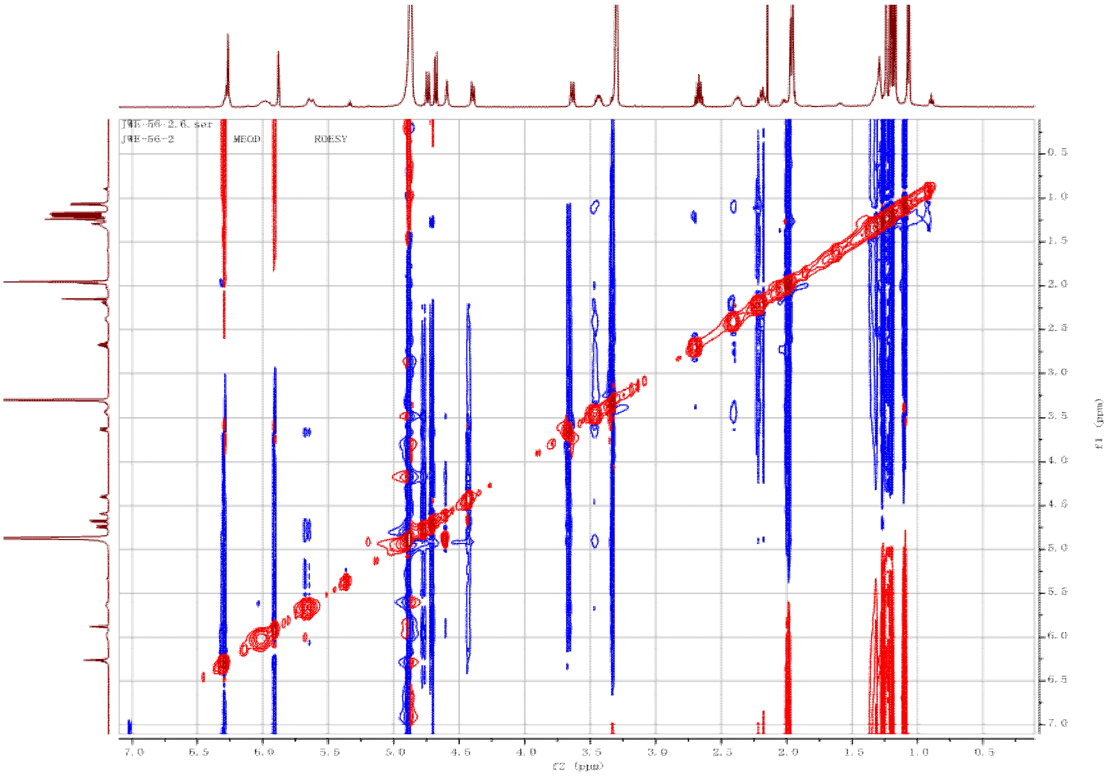

Fig. S5.6 ROESY spectrum (500 MHz) of divarolide D (**5**) in CD_3OD

Auto Print Report 1 2015-12-16 16:09:04

Data Set: Storage 160755 - RawData - D:\2015\JWE-56-2.spc

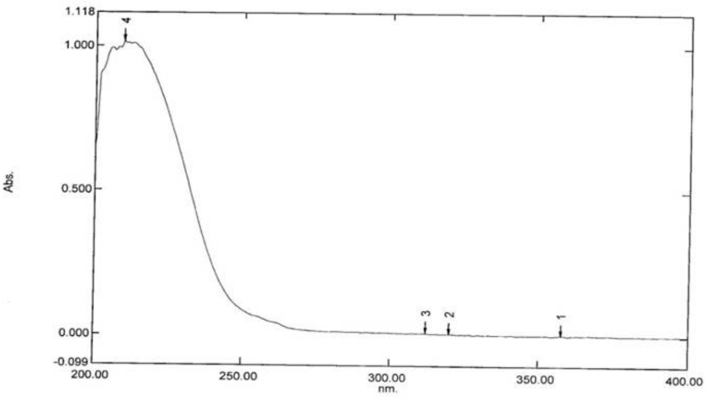

No.	P/V	Wavelength	Abs.	Description
1	⊕	357.50	0.005	
2	⊕	320.00	0.009	
3	⊕	312.00	0.010	
4	⊕	209.50	1.017	

Fig. S5.7 UV spectrum of divarolide D (**5**)

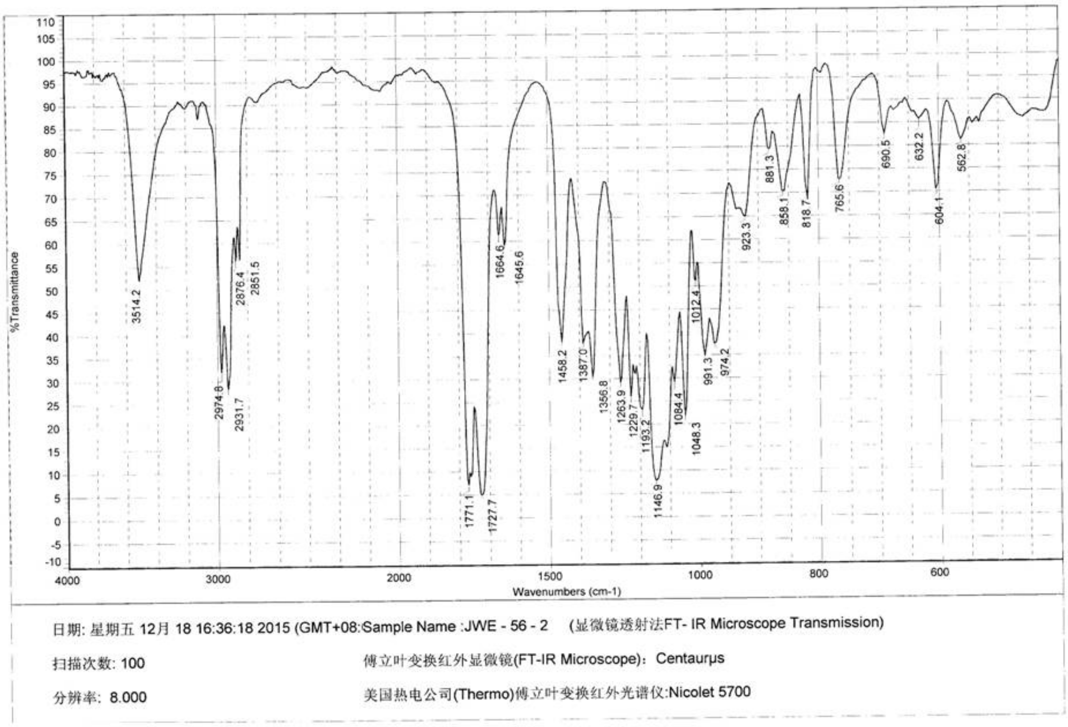

Fig. S5.8 IR spectrum of divarolide D (**5**)

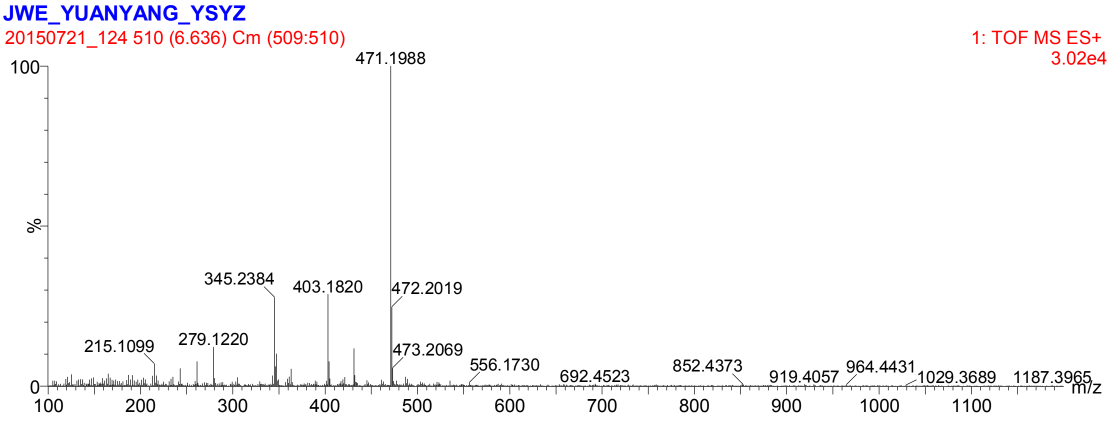

Fig. S5.9 HRESIMS spectrum of divarolide D (**5**)

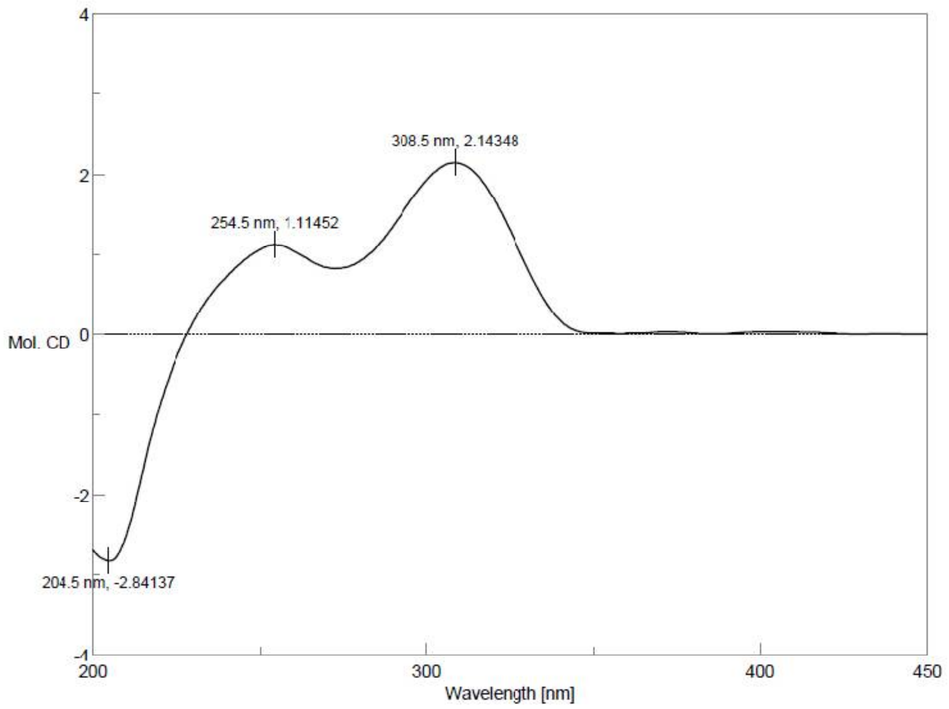

Fig. S5.10 CD spectrum of divarolide D (**5**)

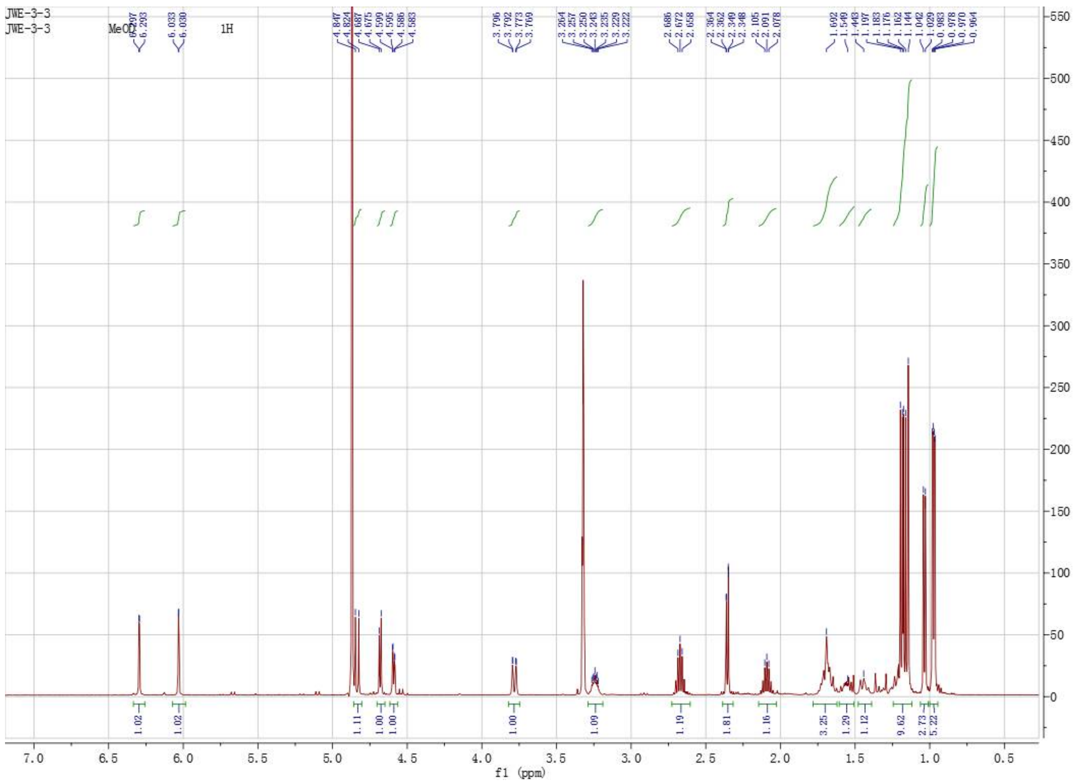

Fig. S6.1 ^{1}H NMR spectrum (500 MHz) of incaspitolide B$_1$ (**6**) in CD_3OD

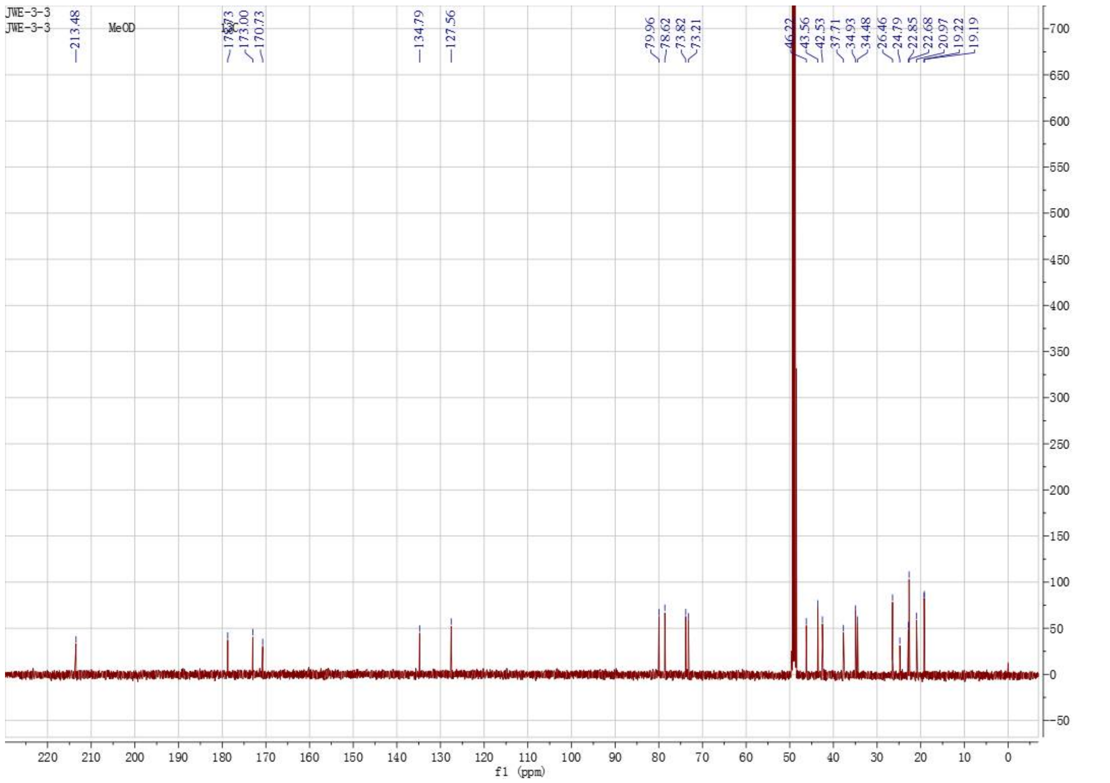

Fig. S6.2 ^{13}C NMR spectrum (500 MHz) of incaspitolide B_1 (**6**) in CD_3OD

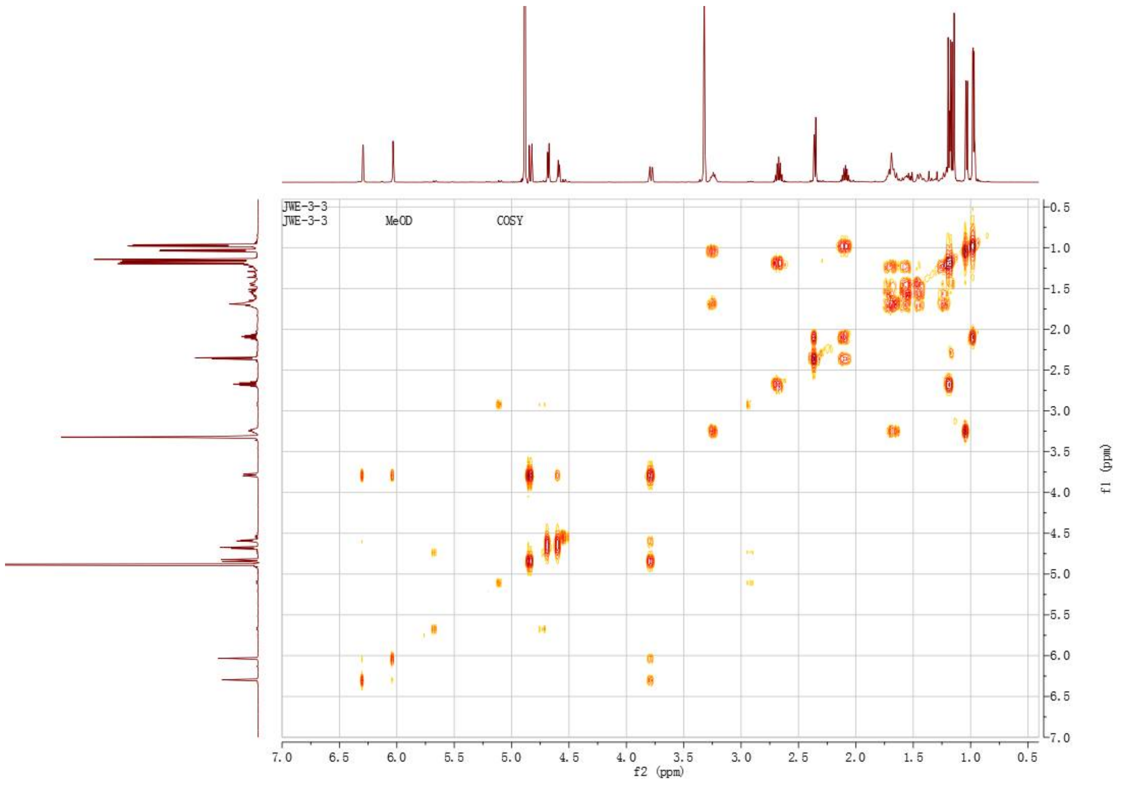

Fig. S6.3 ^{1}H-^{1}H COSY spectrum (500 MHz) of incaspitolide B_1 (**6**) in CD_3OD

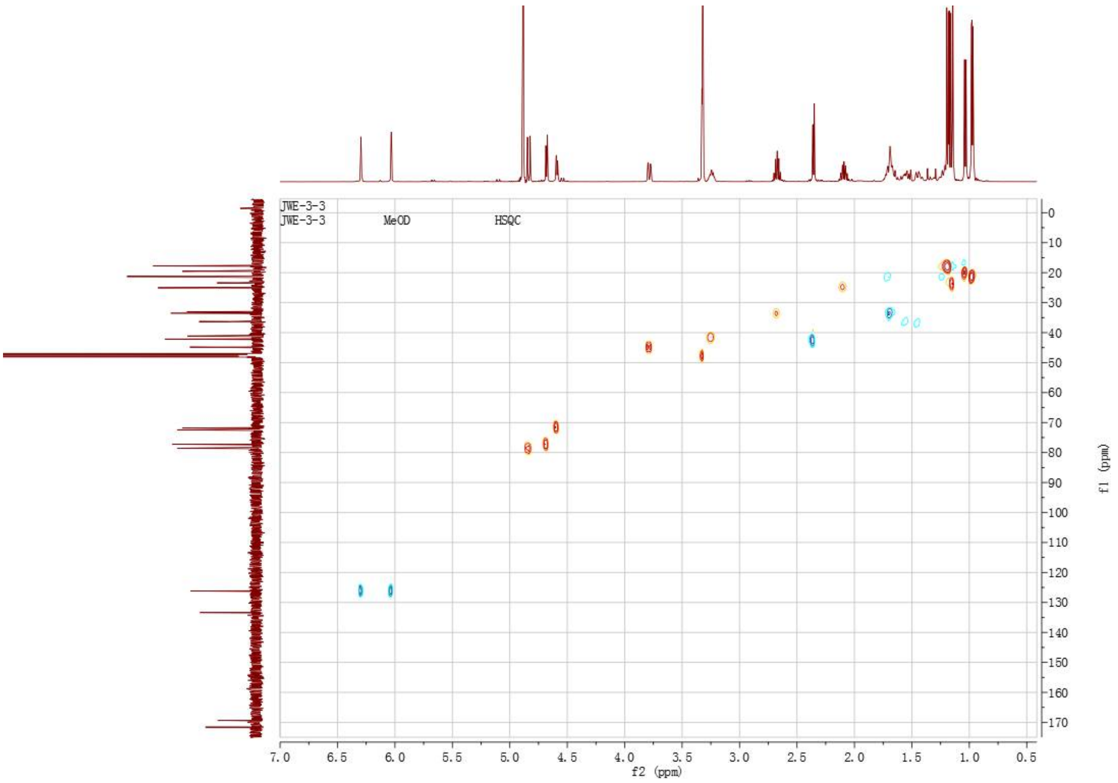

Fig. S6.4 HSQC spectrum (500 MHz) of incaspitolide B_1 (**6**) in CD_3OD

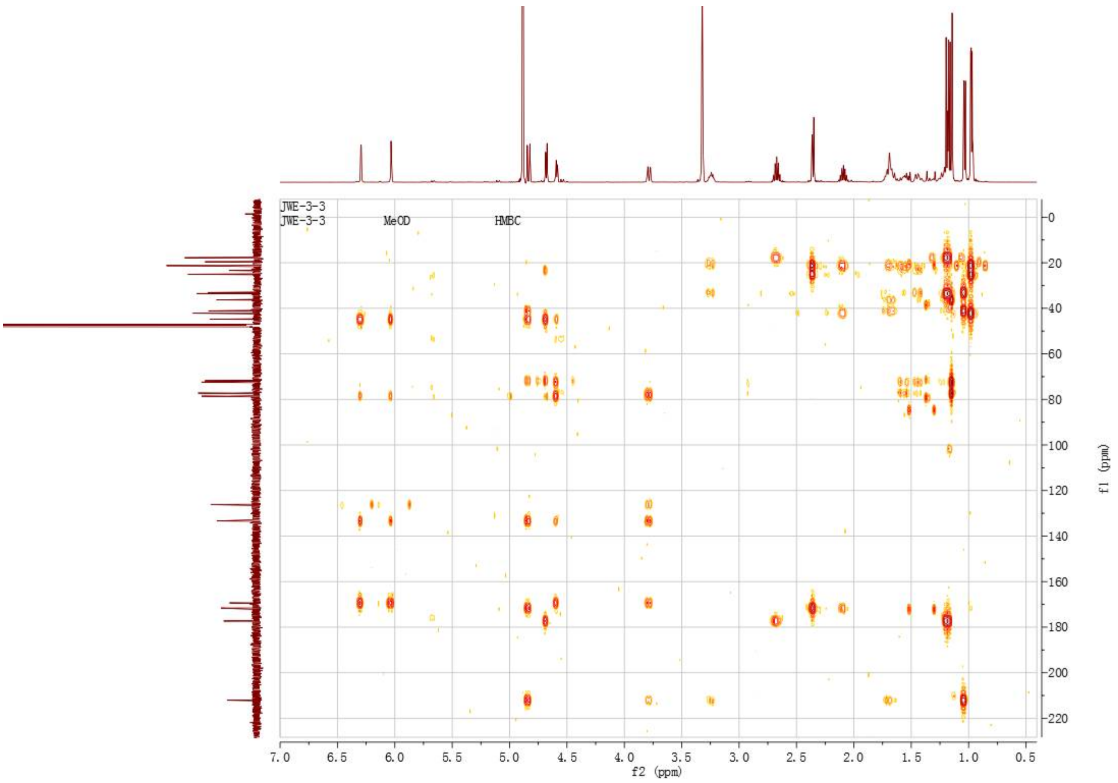

Fig. S6.5 HMBC spectrum (500 MHz) of incaspitolide B_1 (**6**) in CD_3OD

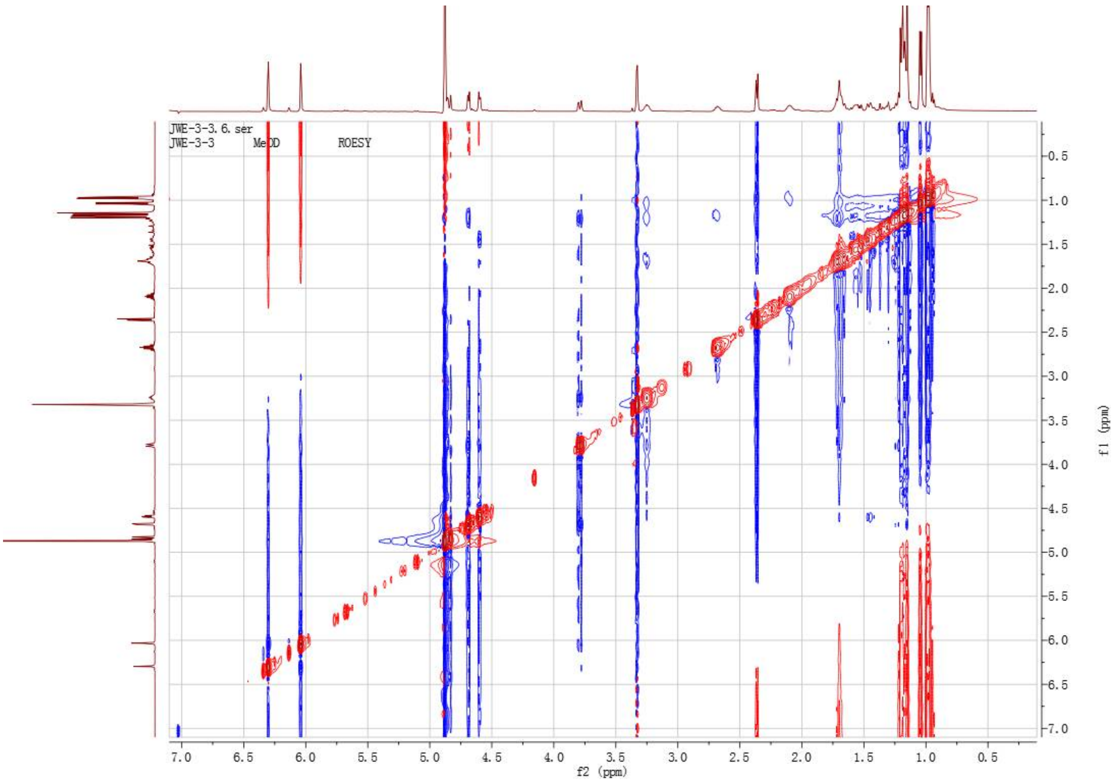

Fig. S6.6 ROESY spectrum (500 MHz) of incaspitolide B_1 (**6**) in CD_3OD

Auto Print Report 1

2015-12-16 16:21:40

Data Set: Storage 162036 - RawData - D:\2015\JWE-3-3.spc

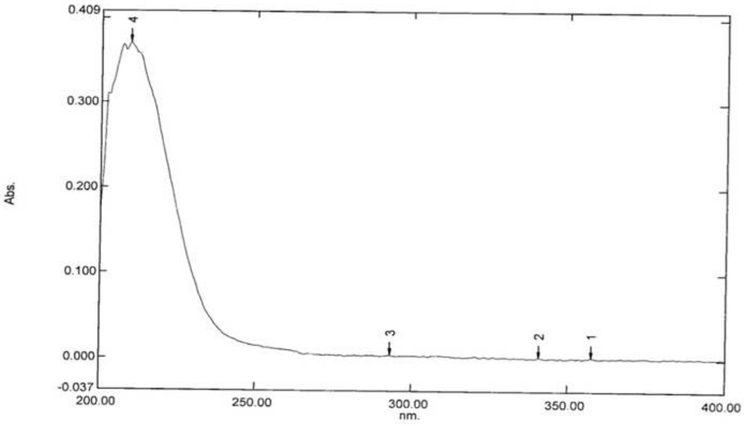

No.	P/V	Wavelength	Abs.	Description
1	⊕	357.50	0.002	
2	⊕	341.00	0.002	
3	⊕	293.00	0.004	
4	⊕	209.50	0.371	

Fig. S6.7 UV spectrum of incaspitolide B_1 (**6**)

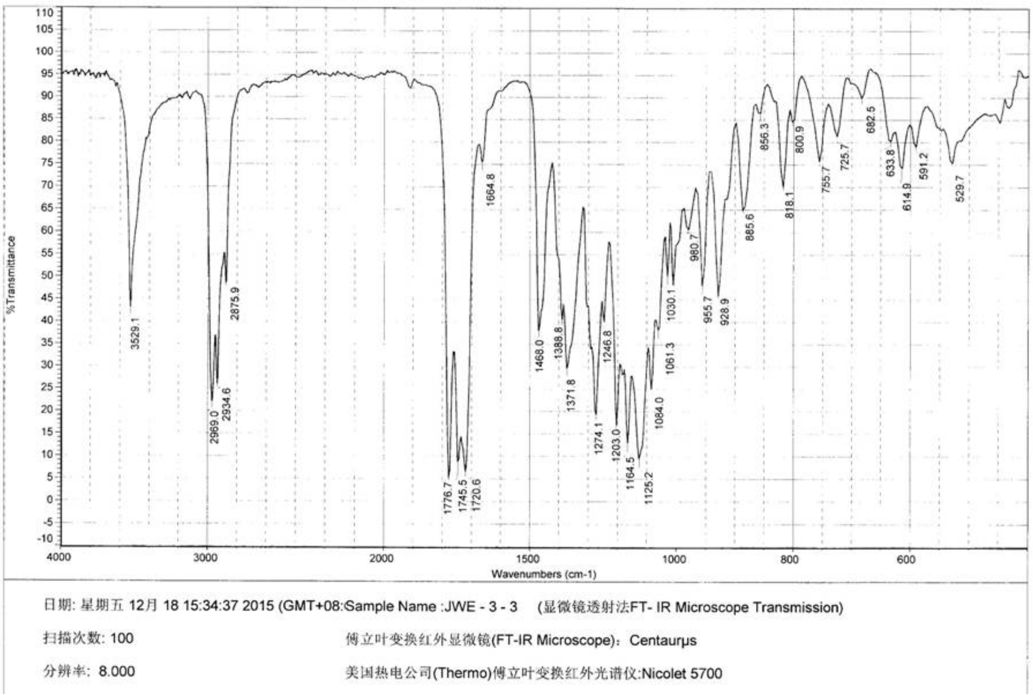

Fig. S6.8 IR spectrum of incaspitolide B_1 (**6**)

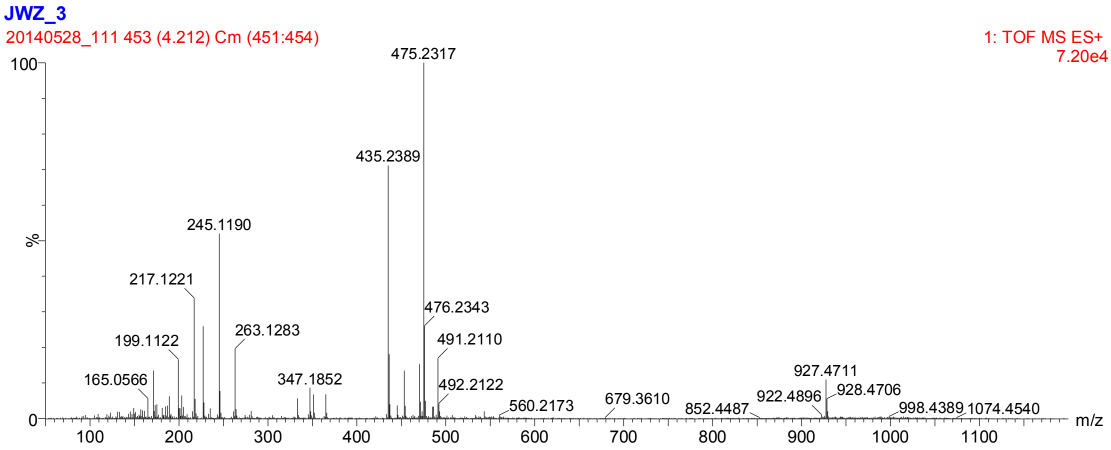

Fig. S6.9 HRESIMS spectrum of incaspitolide B_1 (**6**)

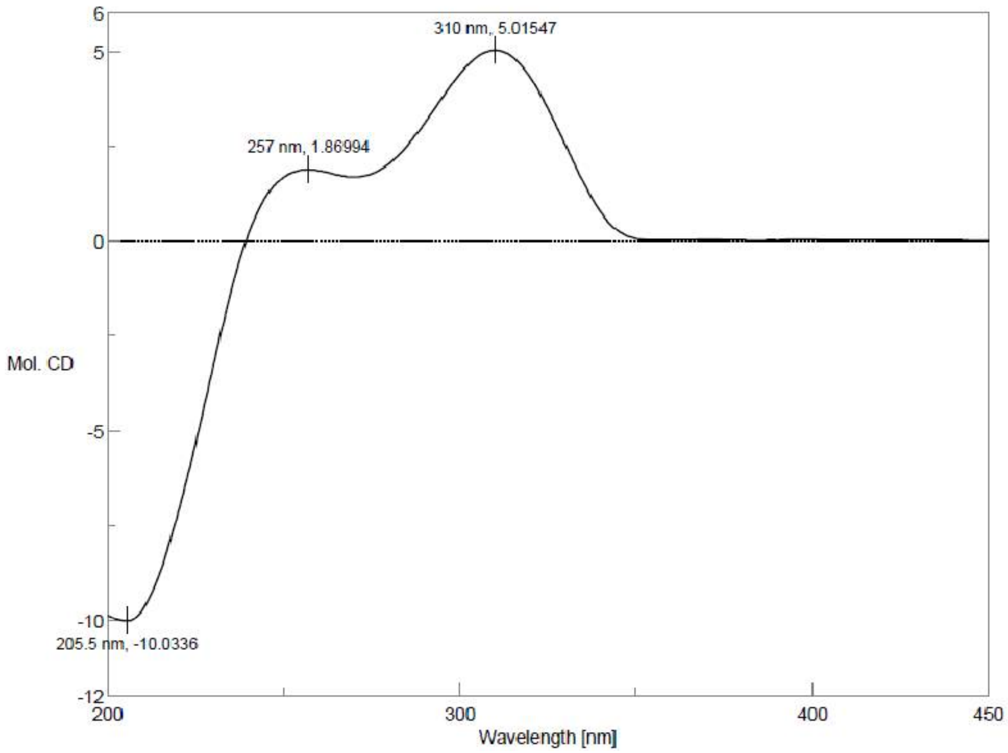

Fig. S6.10 CD spectrum of incaspitolide B_1 (**6**)

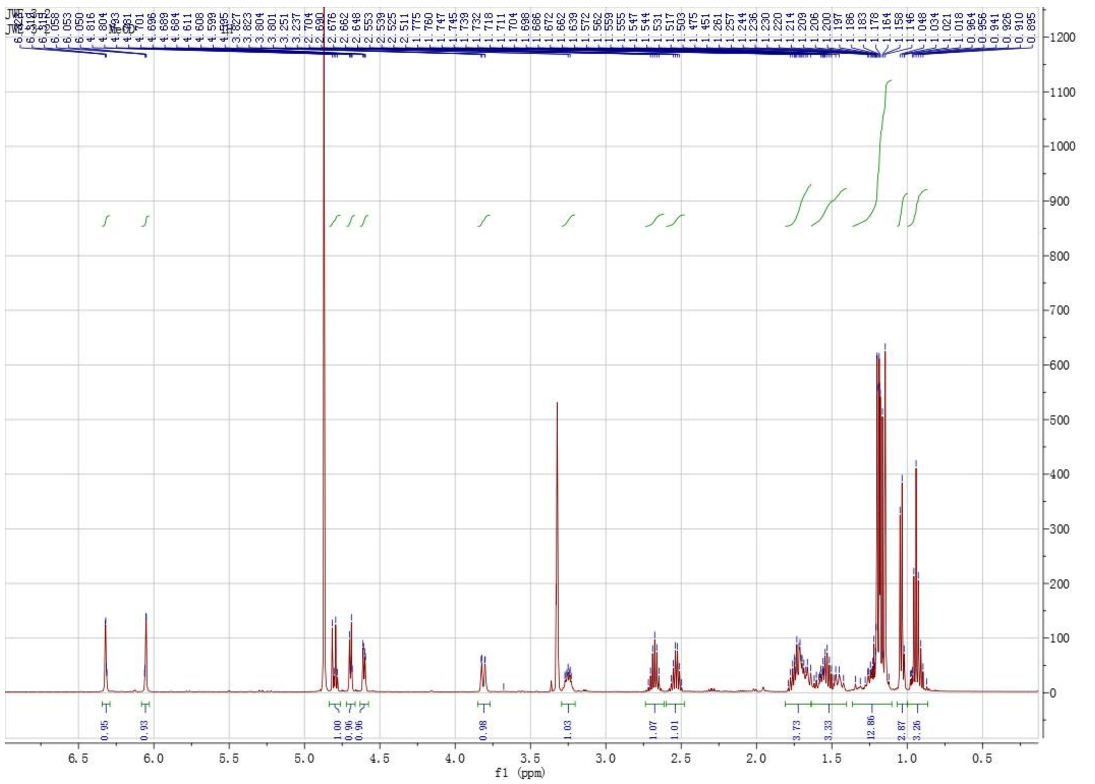

Fig. S7.1 ^{1}H NMR spectrum (500 MHz) of incaspitolide B_2 (**7**) in CD_3OD

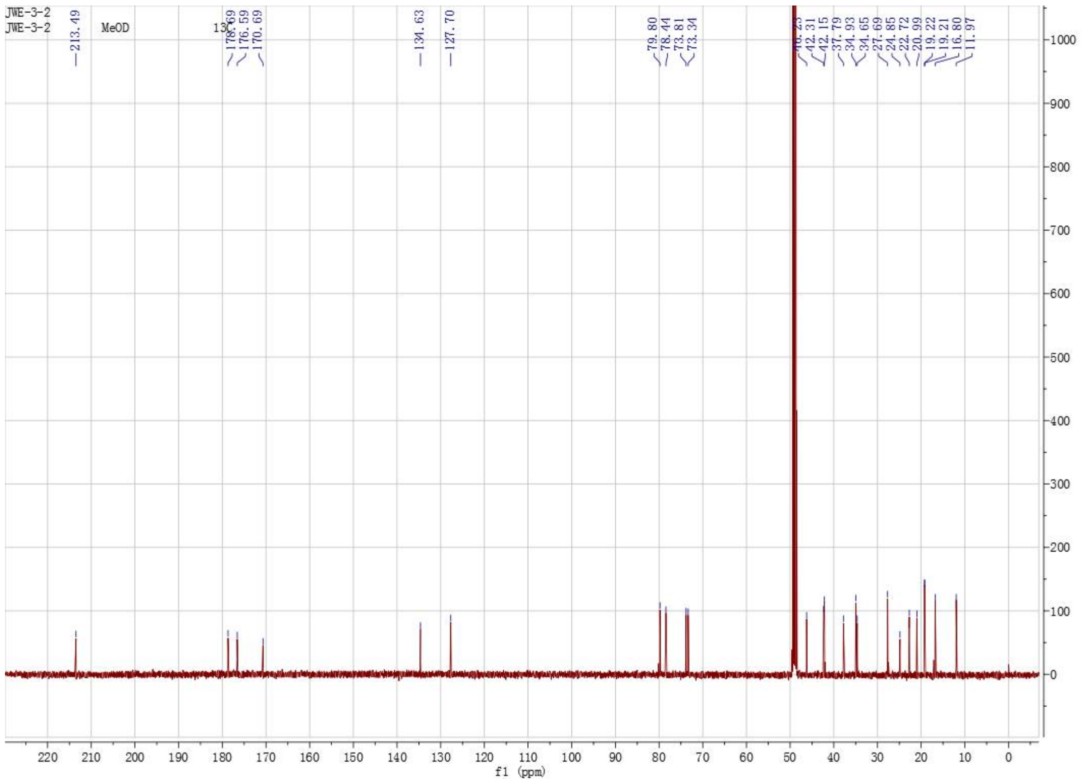

Fig. S7.2 ^{13}C NMR spectrum (500 MHz) of incaspitolide B_2 (**7**) in CD_3OD

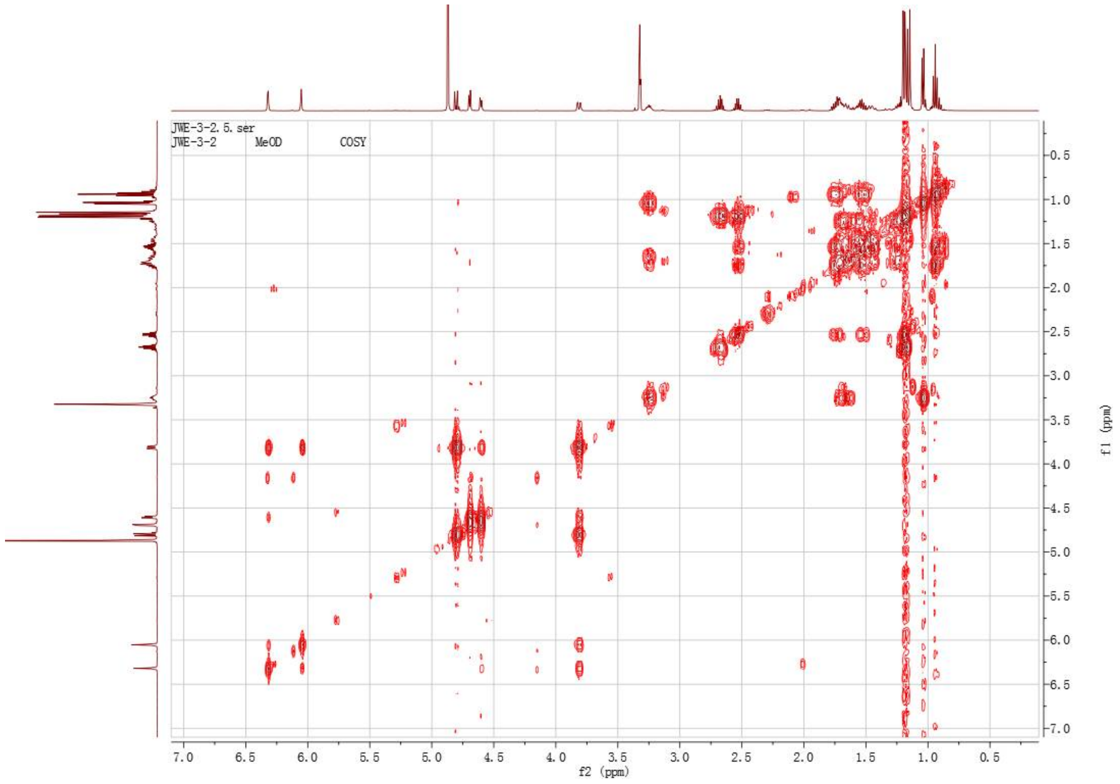

Fig. S7.3 ^{1}H-^{1}H COSY spectrum (500 MHz) of incaspitolide B_2 (**7**) in CD_3OD

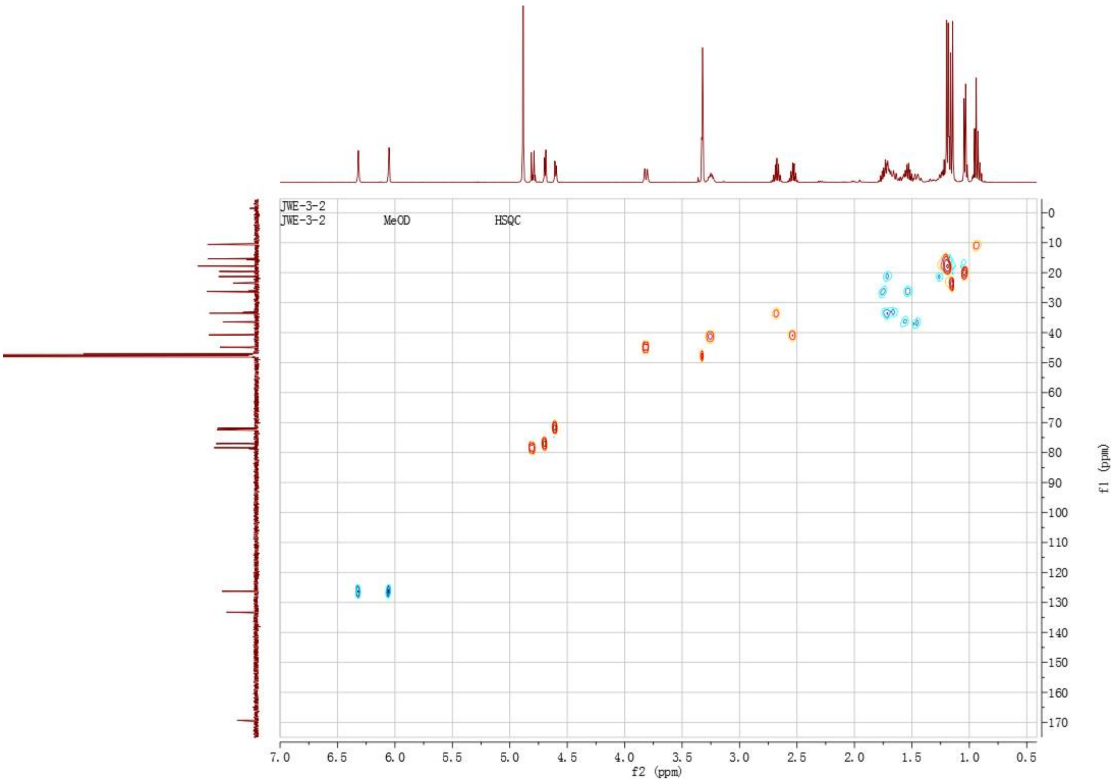

Fig. S7.4 HSQC spectrum (500 MHz) of incaspitolide B_2 (**7**) in CD_3OD

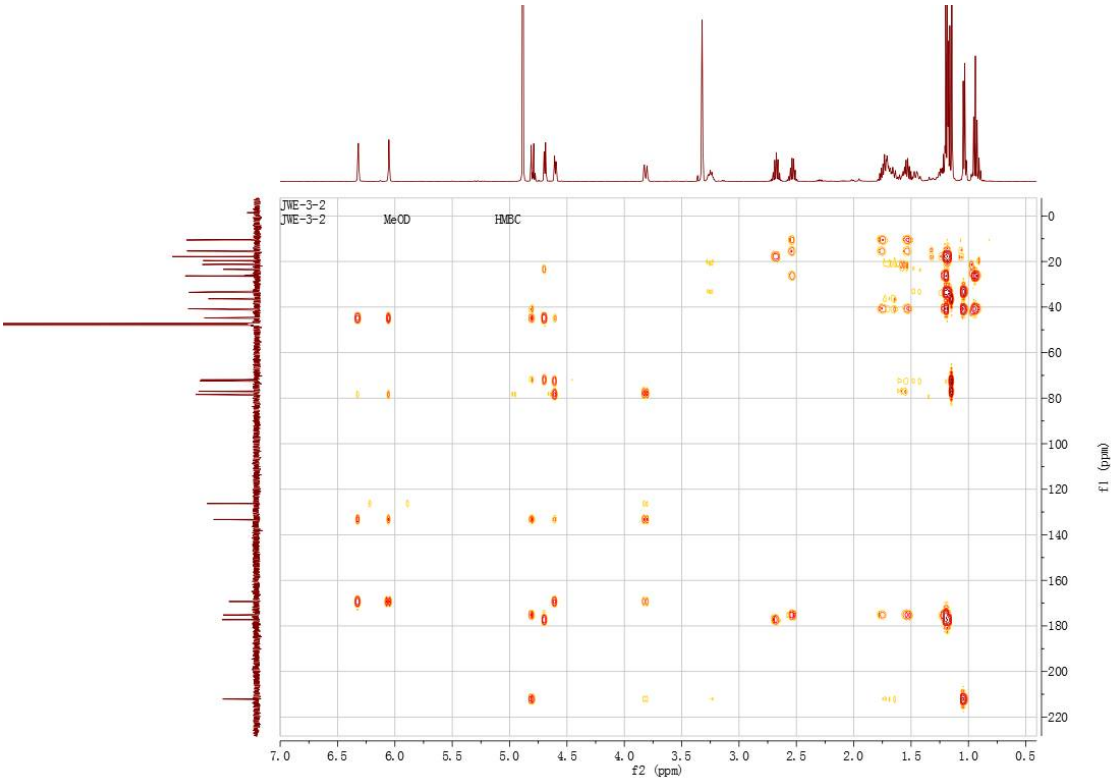

Fig. S7.5 HMBC spectrum (500 MHz) of incaspitolide B_2 (**7**) in CD_3OD

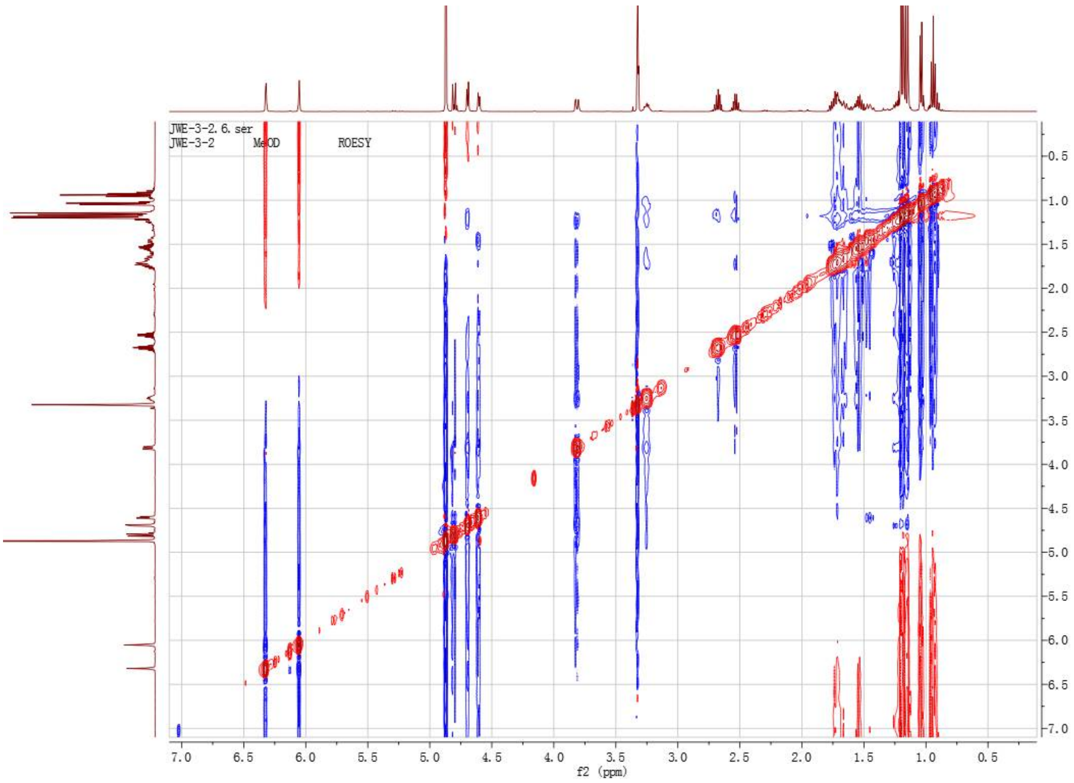

Fig. S7.6 ROESY spectrum (500 MHz) of incaspitolide B_2 (**7**) in CD_3OD

Auto Print Report 1 2015-12-16 14:37:31

Data Set: Storage 143656 - RawData - D:\2015\JWE-3-2.spc

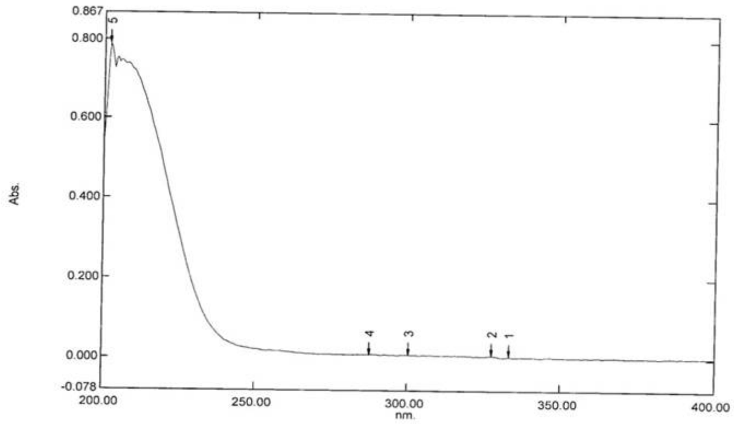

No.	P/V	Wavelength	Abs.	Description
1	⊕	333.50	0.004	
2	⊕	327.50	0.006	
3	⊕	300.50	0.008	
4	⊕	287.50	0.009	
5	⊕	202.00	0.788	

Fig. S7.7 UV spectrum of incaspitolide B_2 (**7**)

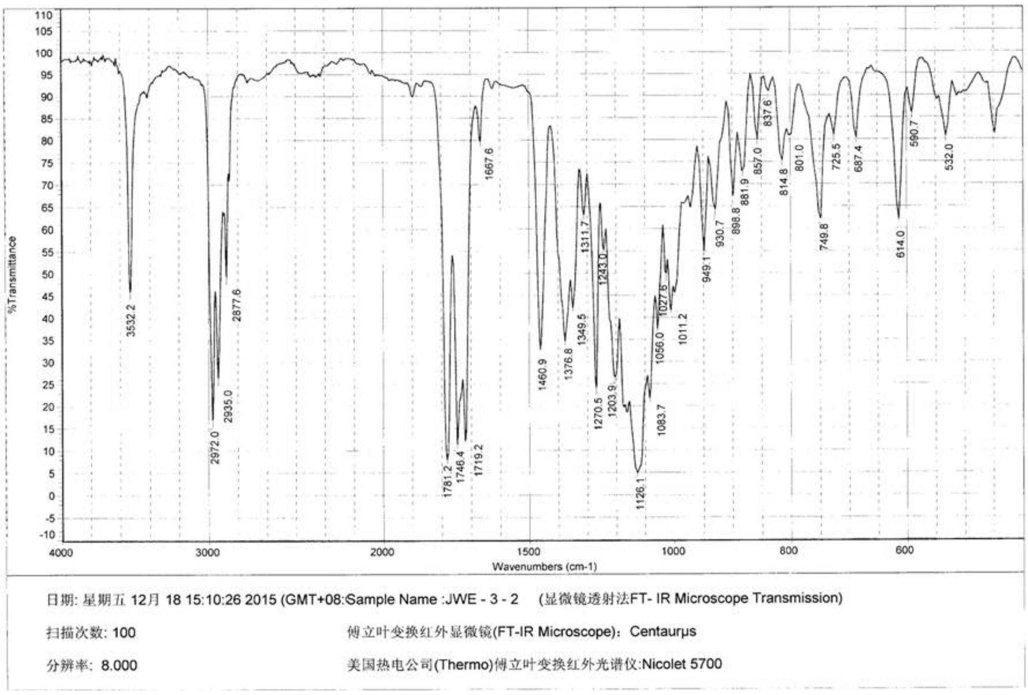

Fig. S7.8 IR spectrum of incaspitolide B_2 (**7**)

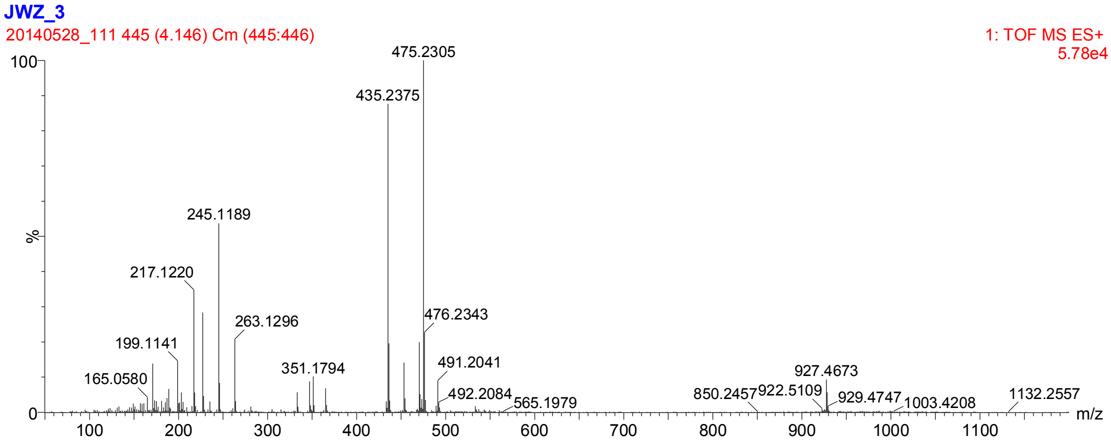

Fig. S7.9 HRESIMS spectrum of incaspitolide B_2 (**7**)

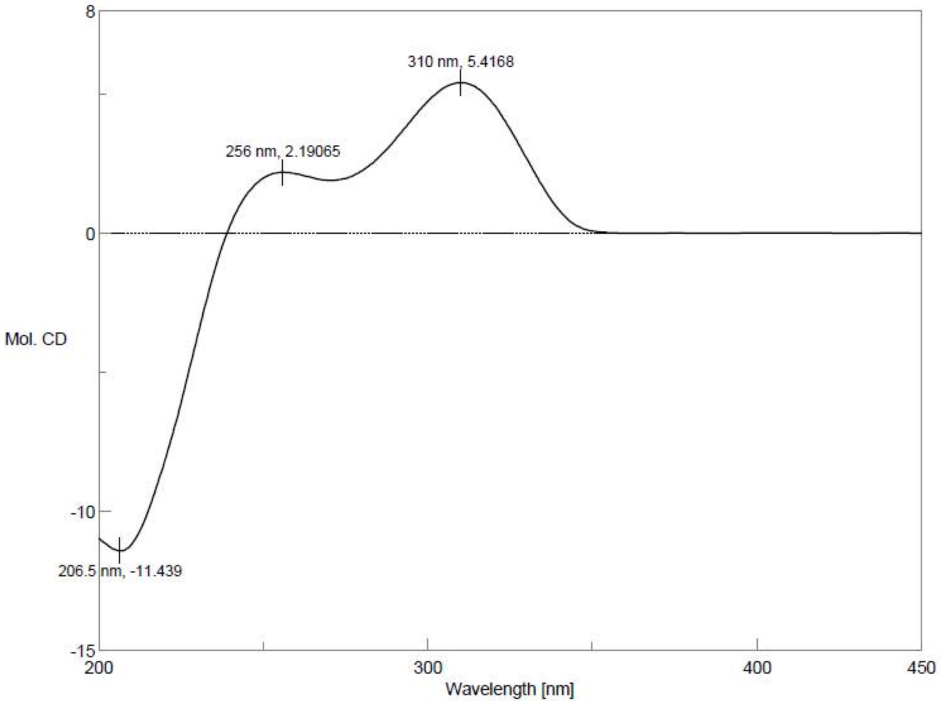

Fig. S7.10 CD spectrum of incaspitolide B_2 (**7**)

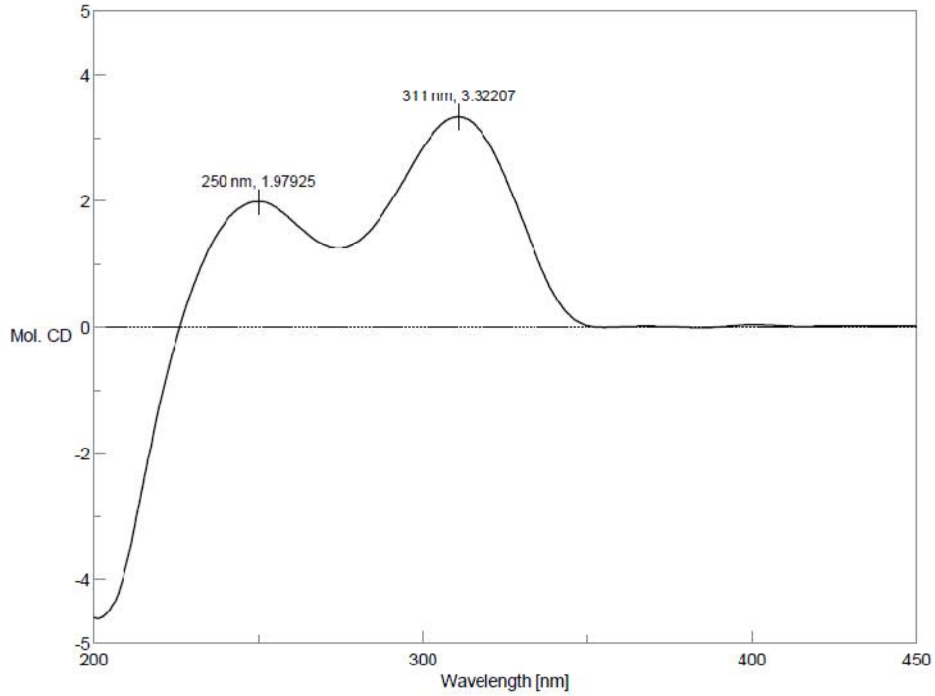

Fig. S8.1 CD spectrum of compound **8**

Table S8.2 ^{1}H and ^{13}C NMR data of compound **8** (δ in CD_3OD; J in Hz).

position	**8**	
	δ_H	δ_C
1	1.68 o, 1.23 m	22.9
2	1.56 m, 1.45 m	37.7
3	1.68 o, 1.68 o	34.5
4		73.8
5	4.68 d (6.0)	78.6
6	4.60 dd (6.0, 1.5)	73.3
7	3.85 dd (11.5, 1.5)	46.3
8	4.92 d (11.5)	79.8
9		213.5
10	3.27 m	42.7
11		134.9
12		170.7
13	6.26 d (1.5), 5.96 d (1.5)	127.4
14	1.00 d (7.0)	20.9
15	1.14 s	24.8
1′		178.7
2′	2.66 m	34.9
3′	1.17 d (7.0)	19.2
4′	1.15 d (7.0)	19.2
1″		167.1
2″		127.4
3″	6.31 q (6.5)	143.1
4″	2.00 br s	20.7
5″	1.99 dq (6.5, 1.5)	16.1

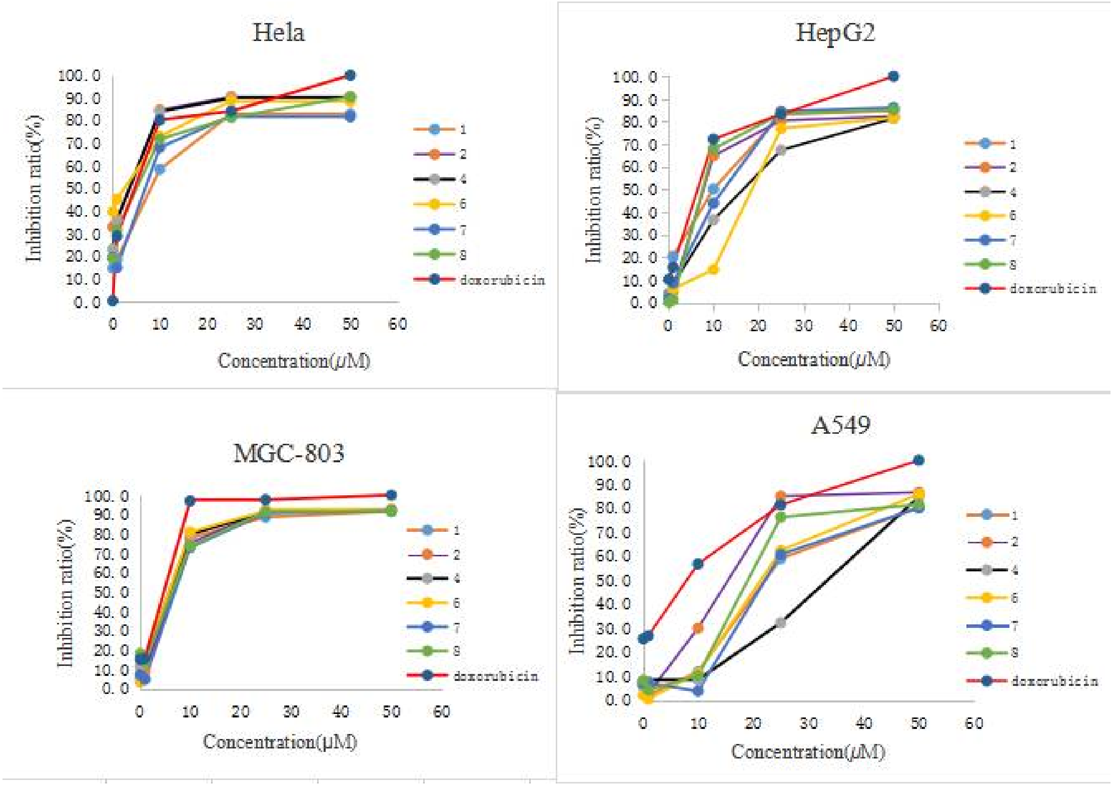

Figure S9 The concentration-response curves of compounds **1**, **2**, **4**, **6**–**8**, and doxorubicin (positive control) for cytotoxicity against four human tumor cell lines.
